# Associations of metabolic changes and polygenic risk scores with cardiovascular outcomes and all-cause mortality across BMI categories: a prospective cohort study

**DOI:** 10.1186/s12933-024-02332-w

**Published:** 2024-07-04

**Authors:** Cancan Li, Xiaoni Meng, Jie Zhang, Haotian Wang, Huimin Lu, Meiling Cao, Shengzhi Sun, Youxin Wang

**Affiliations:** 1 Department of Epidemiology and Health Statistics, School of Public Health, Capital Medical University, No.10 Xitoutiao, Youanmen Street, Fengtai District, Beijing, 100069 China; 2 Beijing Municipal Key Laboratory of Clinical Epidemiology, School of Public Health, Capital Medical University, No.10 Xitoutiao, Youanmen Street, Fengtai District, 100069 Beijing, China; 3 School of Public Health, North China University of Science and Technology, 21 Bohaidadao, Caofeidian, Tangshan, 063210 China; 4 Centre for Precision Medicine, Edith Cowan University, Perth, 6027 Australia

**Keywords:** Cardiovascular disease, All-cause mortality, Metabolic health, Metabolic change, Metabolic syndrome, Polygenic risk scores

## Abstract

**Background:**

Associations between metabolic status and metabolic changes with the risk of cardiovascular outcomes have been reported. However, the role of genetic susceptibility underlying these associations remains unexplored. We aimed to examine how metabolic status, metabolic transitions, and genetic susceptibility collectively impact cardiovascular outcomes and all-cause mortality across diverse body mass index (BMI) categories.

**Methods:**

In our analysis of the UK Biobank, we included a total of 481,576 participants (mean age: 56.55; male: 45.9%) at baseline. Metabolically healthy (MH) status was defined by the presence of < 3 abnormal components (waist circumstance, blood pressure, blood glucose, triglycerides, and high-density lipoprotein cholesterol). Normal weight, overweight, and obesity were defined as 18.5 ≤ BMI < 25 kg/m^2^, 25 ≤ BMI < 30 kg/m^2^, and BMI ≥ 30 kg/m^2^, respectively. Genetic predisposition was estimated using the polygenic risk score (PRS). Cox regressions were performed to evaluate the associations of metabolic status, metabolic transitions, and PRS with cardiovascular outcomes and all-cause mortality across BMI categories.

**Results:**

During a median follow-up of 14.38 years, 31,883 (7.3%) all-cause deaths, 8133 (1.8%) cardiovascular disease (CVD) deaths, and 67,260 (14.8%) CVD cases were documented. Among those with a high PRS, individuals classified as metabolically healthy overweight had the lowest risk of all-cause mortality (hazard ratios [HR] 0.70; 95% confidence interval [CI] 0.65, 0.76) and CVD mortality (HR 0.57; 95% CI 0.50, 0.64) compared to those who were metabolically unhealthy obesity, with the beneficial associations appearing to be greater in the moderate and low PRS groups. Individuals who were metabolically healthy normal weight had the lowest risk of CVD morbidity (HR 0.54; 95% CI 0.51, 0.57). Furthermore, the inverse associations of metabolic status and PRS with cardiovascular outcomes and all-cause mortality across BMI categories were more pronounced among individuals younger than 65 years (*P*_interaction_ < 0.05). Additionally, the combined protective effects of metabolic transitions and PRS on these outcomes among BMI categories were observed.

**Conclusions:**

MH status and a low PRS are associated with a lower risk of adverse cardiovascular outcomes and all-cause mortality across all BMI categories. This protective effect is particularly pronounced in individuals younger than 65 years. Further research is required to confirm these findings in diverse populations and to investigate the underlying mechanisms involved.

**Graphical abstract:**

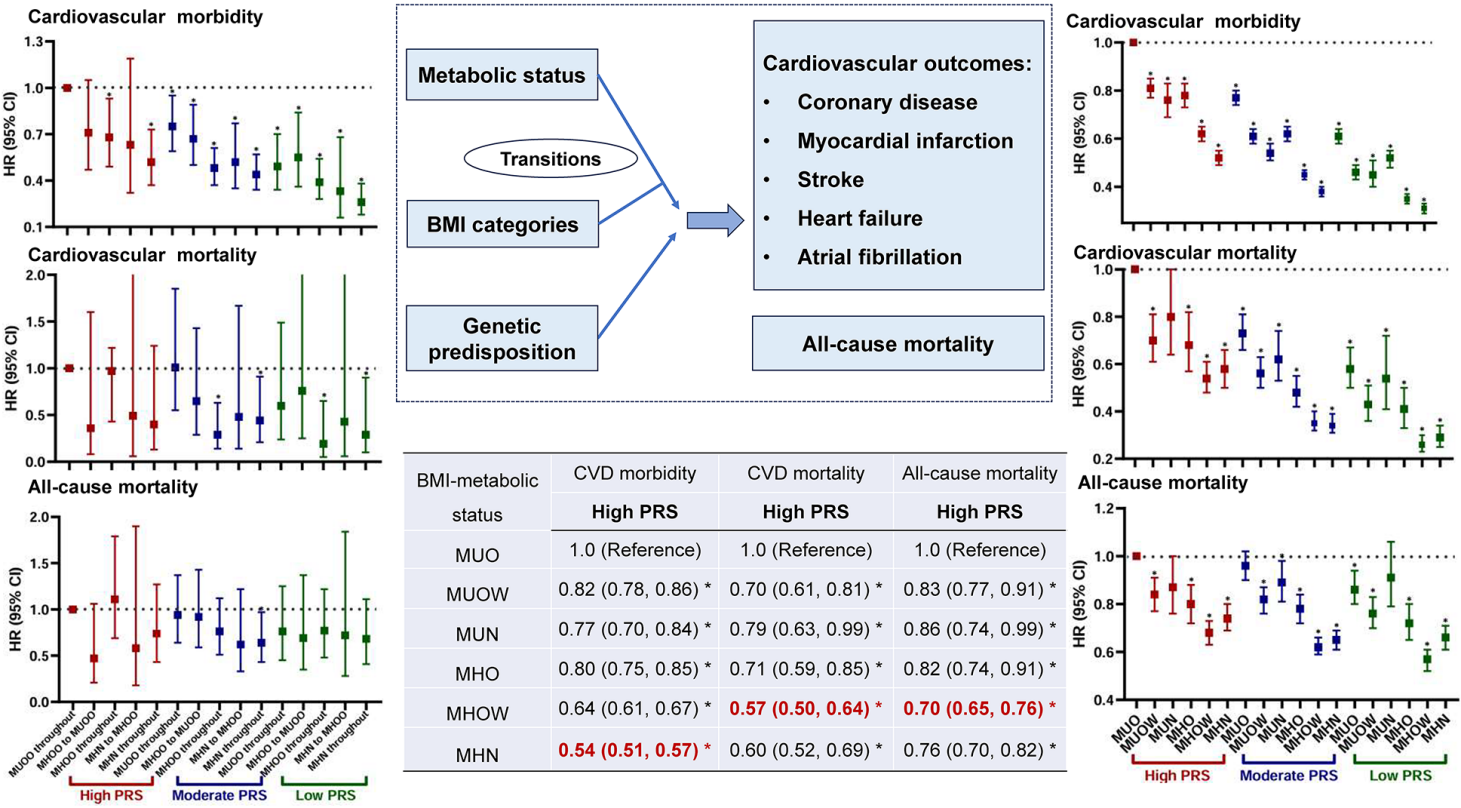

**Supplementary Information:**

The online version contains supplementary material available at 10.1186/s12933-024-02332-w.

## Introduction

Cardiovascular disease (CVD) remain the primary cause of disease burden worldwide, accounting for 18.6 million deaths in 2019 [1]. Metabolic syndrome (MetS), a combination of obesity, hypertension, hyperglycemia, and dyslipidemia, is a well-established risk contributor for CVD [2]. A subset of individuals who are overweight or obesity may not manifest metabolic disorders and can be categorized as metabolically healthy overweight (MHOW) or metabolically healthy obesity (MHO) [3, 4]. Previous studies have confirmed that individuals with the MHO phenotype have a greater CVD risk than those with metabolically healthy normal weight (MHN) phenotype [5, 6]. Given the dynamic nature of metabolic status, research has shown that the metabolic status changes over time across all body mass index (BMI) categories and is associated with cardiovascular risk [6, 7]. However, the role of genetic predisposition on the risk of CVD morbidity and mortality was not considered in the aforementioned observations.

In light of the growing availability of genetic research, accumulating evidence highlights the contribution of genetics to the variation in cardiovascular events [8–10]. Genetic predisposition is commonly assessed through the use of polygenic risk scores (PRSs), providing the potential to identify individuals with increased genetic susceptibility to adverse clinical outcomes [11, 12]. While genetic predisposition is commonly perceived as deterministic, considerable evidence suggests that normal levels of metabolic factors can attenuate the deleterious effects caused by a high genetic risk [13, 14]. This emphasizes the importance of investigating the combined effects of metabolic status and PRS on CVD risk across different BMI categories. Furthermore, it remains unclear how the combined effects of metabolic transitions and PRS influence cardiovascular outcomes and all-cause mortality across BMI groups.

Therefore, in the present study, we aimed to investigate (a) the associations of metabolic status, BMI status, and BMI-metabolic status with cardiovascular outcomes and all-cause mortality stratified by different levels of genetic predisposition, (b) the interaction and joint associations of metabolic status and genetic predisposition with cardiovascular outcomes and all-cause mortality across diverse BMI categories, and (c) the combined effects of metabolic transitions and genetic predisposition on these outcomes according to BMI groups.

## Methods

### Study population

The individual-level data collected from participants enrolled in the UK Biobank (Application Number: 65711) were utilized in this study. The detailed study design and population of the UK Biobank have been previously described [15, 16]. Briefly, the UK Biobank is an ongoing prospective cohort that incorporated data between 2006 and 2010 from 22 assessment centers across the United Kingdom; the participants were aged between 40 and 69 at recruitment. Demographics, healthy lifestyle information, and other potentially health-related information were obtained through touch screen questionnaires, face-to-face interviews, physical examinations, and biological samples.

**Fig. 1:**
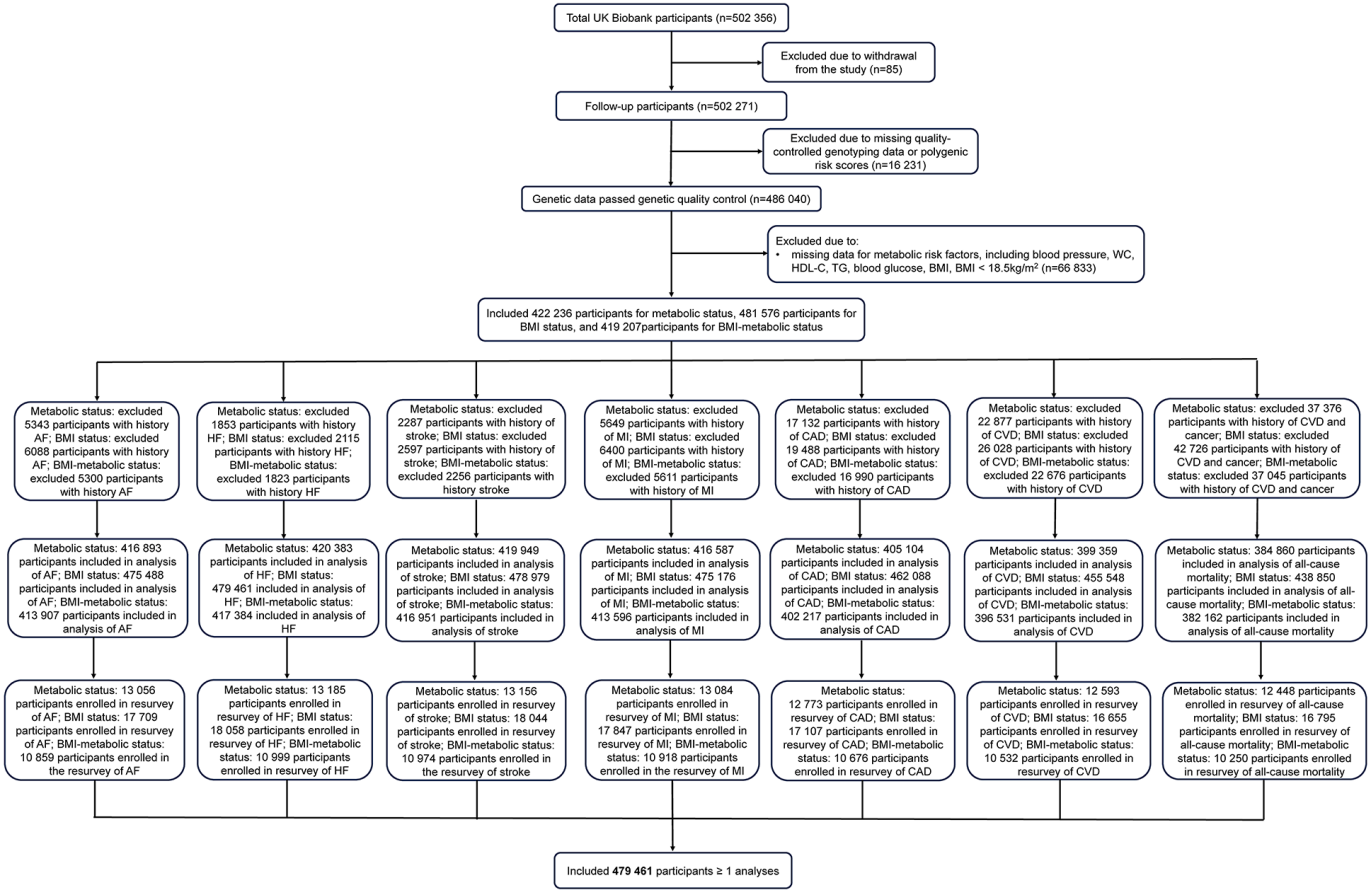
Flow diagram: Selection of participants. *Note* MH status was defined as < 3 abnormal components; WC, waist circumstance; HDL-C, high density lipoprotein-cholesterol; TG, triglycerides; BMI, body mass index; MH, metabolically healthy; MHN, metabolically healthy normal weight; MHOW, metabolically healthy overweight; MHO, metabolically healthy obesity; MUN, metabolically unhealthy normal weight; MUOW, metabolically unhealthy overweight; MUO, metabolically unhealthy obesity; AF, atrial fibrillation; HF, heart failure; MI, myocardial infarction; CAD, coronary disease; CVD, cardiovascular disease

In the present study, among the 502,356 participants, we excluded individuals who withdrew from study (n = 85), had missing quality-controlled genotyping data (n = 16,231), had missing information on metabolic related factors and BMI data, or were underweight (BMI < 18.5 kg/m^2^) (n = 66,833). Furthermore, participants with a prior history of cardiovascular events or cancer were excluded. Finally, 479,461 participants with at least one outcome (cardiovascular outcomes or all-cause mortality) were included. To examine whether transitions in metabolic status, BMI status, and BMI-metabolic status (time window for the transition: from baseline in 2006–2010 to the second survey in 2012–2013) altered the aforementioned outcomes, 18,058 participants were enrolled in the subsequent analysis (Fig. 1).

### Assessment of metabolic health status and BMI categories and their transitions

According to the National Cholesterol Education Program-Adult Treatment Panel III (NCEP-ATP III) criteria [17], MetS was defined as the presence of 3 or more following abnormal components: (1) waist circumference > 102 cm in men and > 88 cm in women; (2) systolic blood pressure (SBP) ≥ 130 mmHg or diastolic blood pressure (DBP) ≥ 85 mmHg or antihypertensive agents; (3) serum glucose ≥ 6.1 mmol/L or antidiabetic agents; (4) serum triglyceride (TG) ≥ 1.7 mmol/L or antihyperlipemic agents; (5) high-density lipoprotein cholesterol (HDL-C) < 1.0 mmol/L in men and < 1.3 mmol/L in women or antihyperlipemic agents. Hence, metabolically healthy (MH) status was defined as < 3 abnormal components and metabolically unhealthy (MU) status was defined as ≥ 3 abnormal components [18]. BMI categories were classified into three categories based on WHO guideline [19]: normal weight (18.5 ≤ BMI < 25 kg/m^2^), overweight (25 ≤ BMI < 30 kg/m^2^), and obesity (BMI ≥ 30 kg/m^2^). According to the combination of metabolic status and BMI categories, we classified participants into 6 groups: MHN, MHOW, MHO, metabolically unhealthy normal weight (MUN), metabolically unhealthy overweight (MUOW), and metabolically unhealthy obesity (MUO).

Furthermore, transitions in metabolic status (MH throughout, MH to MU, and MU throughout), BMI status (normal weight throughout, normal weight to overweight, overweight throughout, overweight to obesity, and obesity throughout), and BMI-metabolic status (MHN throughout, MHN to metabolically healthy overweight or obesity [MHOO], MHOO throughout, MHOO to metabolically unhealthy overweight or obesity [MUOO], and MUOO throughout) were characterized from baseline to the second survey.

### Definition of genetic predisposition

To evaluate genetic predisposition, PRSs for CVD, coronary disease (CAD), myocardial infarction (MI), stroke, heart failure (HF), and atrial fibrillation (AF) were constructed for each participant. In brief, the PRSs of CVD, CAD, and AF were extracted from ‘Standard PRS (Category 301)’ provided by the UK Biobank PRS Release. Furthermore, 31 single SNPs related to MI, 32 SNPs related to stroke, and 12 SNPs related to HF were employed to determine the PRSs for MI, stroke, and HF, respectively, as reported in published genome-wide association studies (Tables S1–S3) [8–10]. To mitigate the impact of SNP deletions, we utilized the following formula to calculate the PRSs for each individual [20]:

1 $$ {\text{PRS}}_{{\text{j}}} = \mathop \sum \limits_{{\text{j}}} \frac{{{\text{S}}_{{\text{i}}} \times {\text{G}}_{{{\text{ij}}}} }}{{M_{{\text{j}}} }} $$

In this formula, ‘S’ presents the effect value (beta/odds ratio), ‘G’ symbolizes the allele dose (with each SNP being recoded as 0, 1, or 2, according to the number of risk alleles), and ‘M’ presents the total number of SNPs. The subscript ‘i’ denotes the sequence number of the SNP, whereas the subscript ‘j’ pertains to the sequence number of the individual. Additionally, we categorized individuals into three distinct groups in line with their PRSs: low (quintile 1), intermediate (quintiles 2–4), and high (quintile 5), as detailed previously.

### Follow-up and outcome ascertainment

Participants without CVD or cancer were followed up from the date of baseline examination until the first occurrence of current study outcomes, loss to follow-up, or the censoring date (October 12, 2023, defined as the end date of disease and mortality data collection), whichever came first.

The primary outcomes included all-cause mortality, nonfatal CVD morbidity, and nonfatal CVD mortality. Nonfatal CVD consists of nonfatal CAD (I20–I25), MI (I21–I23, I24.1, and I25.2), stroke (I60-I64), HF (I11.0, I13.0, I13.2, I50.X), and AF (I48), which were identified according to International Classification of Diseases, Tenth Revision (ICD-10) codes. The records on the incidence of CVD, CAD, MI, stroke, HF, and AF were obtained by linking with the primary care system, hospital inpatient records, and the death registry. Mortality information was determined by matching with the death registries of the National Health Service Information Centre [15].

### Assessment of covariates

A series of covariates in the present study were obtained through touch-screen questionnaires or face-to-face interviews, including age, sex, race (white, mixed, Asian or Asian British, black or black British, Chinese, and other), Townsend Deprivation Index (with higher values representing lower socioeconomic status), annual household income (£; < 18,000, 18,000–30,999, 31,000–51,999, 52,000–100,000, > 100,000), educational attainment, sleep duration, healthy diet (yes or no), physical activity (low, middle, or high), smoking status (never, previous, or current), and alcohol intake frequency (never, special occasion only, one to three times a month, once or twice a week, three or four times a week, and daily or almost daily). Levels of educational attainment were classified into 6 levels: (1) no qualifications, (2) Certificate of Secondary Education or Ordinary Levels/General Certificate of Secondary Education or equivalent, (3) Advanced Levels/Advanced Subsidiary Levels or equivalent, (4) other professional qualification, (5) National Vocational Qualification or Higher National Certificate or equivalent, and (6) college or university degree [21]. A healthy diet was based on eating at least 5 portions of a variety of fruits and vegetables every day, following the NHS guidelines [22]. The self-reported physical activity level was assessed using the well-validated International Physical Activity Questionnaire-Short Form [23]. We addressed missing covariates by employing a missing indicator category for categorical variables and substituting mean values for continuous variables.

### Statistical analysis

The participants’ baseline characteristics, encompassing sociodemographic characteristics, socioeconomic status factors, and metabolic risk factors, are presented as mean ± standard deviation (SD) for continuous variables and as percentages for categorical variables. The chi-square (χ^2^) test was used for comparing categorical variables, while analysis of variance or Student’s t test was performed for continuous variables. Cox proportional hazard models, with duration of follow-up as the time scale, were utilized to evaluate the associations of exposures (metabolic status, BMI status, BMI-metabolic status, and their transitions) and PRS with cardiovascular outcomes and all-cause mortality across BMI categories. The proportional hazard assumption was examined using Schoenfeld residuals. Two Cox proportional hazard models were fitted. Model 1 was adjusted for age, sex, race, Townsend Deprivation Index, annual household income, educational attainment, 22 assessment centers, and the first 5 principal components of ancestry. Model 2 was further adjusted for family history of diabetes, family history of high blood pressure, and lifestyle factors including sleep duration, healthy diet, physical activity, smoking status, and alcohol intake frequency, based on Model 1. Furthermore, the subgroup analysis of females was additionally adjusted for pregnancy history and menopausal status.

We analysed the effect of metabolic status, BMI status, and BMI-metabolic status on all-cause mortality, CVD morbidity, and CVD mortality stratified by different levels of PRS. To investigate the joint association between exposures (metabolic status, BMI status, BMI-metabolic status, and their transitions) and PRS, we established the following new product terms: six categories for metabolic status and PRS (2 × 3), nine categories for BMI status and PRS (3 × 3), eighteen categories for BMI-metabolic status and PRS (6 × 3), nine categories for transitions in metabolic status and PRS (3 × 3), fifteen categories for transitions in BMI status and PRS (5 × 3), and fifteen categories for transitions in BMI-metabolic status and PRS (5 × 3). Hazard ratios (HRs) and their 95% confidence intervals (CIs) for each outcome across these groups were calculated. Likelihood ratio tests were used to evaluate the significance of the multiplicative interaction term by comparing models with and without this term.

Several subgroup analyses were conducted to examine the stability and possible variations of the primary results, stratified by age (< 65, ≥ 65 years), sex (male, female), and ethnicity (White, non-White), with corresponding tests for interaction. Furthermore, we performed several sensitivity analyses: using different definitions of MH status (< 2 or < 1 abnormal components); excluding individuals whose CVD cases or deaths occurred in the first 2 years of follow-up; and analysing the interplay between genetic risk and lifestyle factors. In addition, we conducted Wald tests comparing the coefficients of baseline exposures to address the survival bias introduced by including only participants who survived until the second survey, with a detailed description provided in the Supplementary Method. R software 4.1.1 (R Development Core Team, Vienna, Austria) was used for the analyses, and a two-tailed *P* value less than 0.05 was considered to indicate statistically significant.

## Results

### Baseline characteristics of participants according to metabolic status and BMI-metabolic status

As shown in Table 1, of the 481,576 participants (mean age 56.55 years, 45.9% male, 94.3% White), 422,236 participants were in metabolic status (MH: 70.2%; MU: 29.8%) and 419,207 participants in BMI-metabolic status (MHN, 30.0%; MHOW: 31.0%; MHO: 9.1%; MUN: 2.6%; MUOW: 11.9%; MUO: 15.4%). Baseline characteristics showed that participants with MH or MHN status tended to be younger, female, more educated, more physically active, smoke less, and have higher income (*P* < 0.001). A lower BMI, WC, SBP, DBP, TG, and serum glucose, along with a higher HDL-C, were also observed in participants with MH or MHN status (*P* < 0.001).

**Table 1:** Baseline characteristics of participants according to metabolic status and BMI-metabolic status

Characteristics	Total (n = 481,576)	Metabolic status (n = 422,236)		BMI-metabolic status (n = 419,207)						*P*^a^	*P*^b^
		MH (n = 296,495, 70.2%)	MU (n = 125,741, 29.8%)	MHN (n = 125,887, 30.0%)	MHOW (n = 129,751, 31.0%)	MHO (n = 38,279, 9.1%)	MUN (n = 10,757, 2.6%)	MUOW (n = 49,970, 11.9%)	MUO (n = 64,563, 15.4%)		
Age, year, mean (SD)	56.55 (8.09)	56.65 (8.14)	58.71 (7.55)	55.31 (8.19)	56.00 (8.11)	55.56 (7.99)	60.52 (6.97)	59.67 (7.26)	57.61 (7.71)	< 0.001	< 0.001
Male, no (%)	220,940 (45.9)	120,109 (40.5)	75,188 (59.8)	41,025 (32.6)	63,987 (49.3)	14,452 (37.8)	7328 (68.1)	32,409 (64.9)	35,139 (54.4)	< 0.001	< 0.001
Townsend Deprivation Index, mean (SD)	− 1.32 (3.08)	− 1.43 (3.02)	− 1.05 (3.50)	− 1.53 (2.98)	− 1.50 (2.98)	− 0.93 (3.22)	− 1.28 (3.16)	− 1.33 (3.08)	− 0.80 (3.26)	< 0.001	< 0.001
Ethnicity, no (%)										< 0.001	< 0.001
White	454,309 (94.3)	280,062 (94.5)	118,234 (94.0)	119,762 (95.1)	122,748 (94.6)	35,206 (91.2)	9801 (91.1)	46,960 (94.0)	61,118 (94.7)		
Mixed	2798 (0.6)	1898 (0.6)	562 (0.5)	825 (0.7)	741 (0.6)	303 (0.8)	44 (0.4)	204 (0.4)	313 (0.5)		
Asian or Asian British	9140 (1.9)	4708 (1.6)	3360 (2.7)	1999 (1.6)	2106 (1.6)	517 (1.4)	577 (5.4)	1545 (3.1)	1170 (1.8)		
Black	7503 (1.6)	4921 (1.7)	1602 (1.3)	1140 (0.9)	2199 (1.7)	1554 (4.1)	96 (0.9)	478 (1.0)	1020 (1.6)		
Chinese	1461 (0.3)	1053 (0.4)	238 (0.2)	716 (0.6)	285 (0.2)	27 (0.1)	85 (0.8)	113 (0.2)	39 (0.1)		
Other	4241 (0.9)	2594 (0.9)	1123 (0.9)	982 (0.8)	1104 (0.9)	468 (1.2)	110 (1.0)	446 (0.9)	558 (0.9)		
Unknown	2124 (0.4)	1259 (0.4)	622 (0.5)	463 (0.4)	568 (0.4)	204 (0.5)	44 (0.4)	224 (0.5)	345 (0.5)		
Education, no (%)										< 0.001	< 0.001
No qualification	82,041 (17.0)	42,831 (14.5)	29,332 (23.3)	14,947 (11.9)	20,192(156)	7278 (19.0)	2288 (21.3)	11,412 (22.8)	15,489 (24.0)		
CSE or ordinary levels/GCSE or equivalent	80,374 (16.7)	50,113 (16.9)	20,437(16.3)	20,728 (16.5)	21,967(16.9)	7024 (18.4)	1620 (15.1)	7914 (15.8)	10,830 (16.8)		
Advanced levels/advanced subsidiary levels or equivalent	26,296 (5.5)	16,862 (5.7)	6193(4.9)	7574 (6.0)	7141(5.5)	1992 (5.2)	516 (4.8)	2486 (5.0)	3174 (4.9)		
Other professional qualification	57,310 (11.9)	35,258 (11.9)	15,057 (12.0)	14,926 (11.9)	15,489 (11.9)	4566 (11.9)	1294 (12.0)	6013 (12.0)	7712 (11.9)		
NVQ or HNC or equivalent	73,873 (15.3)	43,965 (14.8)	20,793 (16.5)	16,055 (12.8)	20,889 (16.1)	6765 (17.7)	1478 (13.7)	7807 (15.6)	11,437 (17.7)		
College or university degree	155,593 (32.3)	104,358 (35.2)	32,021 (25.5)	50,528 (40.1)	42,639 (32.9)	10,156 (26.5)	3406 (31.7)	13,632 (27.3)	14,891 (23.1)		
Unknown	6089 (1.3)	3108 (1.1)	1908 (1.5)	1129 (0.9)	1434 (1.1)	498 (1.3)	155 (1.4)	706 (1.4)	1030 (1.6)		
Annual household income, £, no (%)										< 0.001	< 0.001
< 18,000	93,007 (19.3)	50,611 (17.1)	31,370 (25.0)	20,242 (16.1)	21,746 (16.8)	7918 (20.7)	2731 (25.4)	11,948 (23.9)	16,505 (25.6)		
18,000–30,999	104,667 (21.7)	62,350 (21.0)	29,267 (23.3)	26,059 (20.7)	27,623 (21.3)	8168 (21.3)	2717 (25.3)	11,907 (23.8)	14,558 (22.6)		
31,000–51,999	107,431 (22.3)	68,849 (23.2)	25,280 (20.1)	29,170 (23.2)	30,696 (23.7)	8516 (22.3)	1962 (18.2)	10,251 (20.5)	13,020 (20.2)		
52,000–100,000	83,878 (17.4)	56,904 (19.2)	16,644 (13.2)	25,155 (20.0)	25,165 (19.4)	6258 (16.4)	1320 (12.3)	6726 (13.5)	8576 (13.3)		
> 100,000	22,293 (4.6)	15,836 (5.3)	3685 (2.9)	7585 (6.0)	6712 (5.2)	1425 (3.7)	327 (3.0)	1608 (3.2)	1744 (2.7)		
Unknown	70,300 (14.6)	41,945 (14.2)	19,495 (15.5)	17,676 (14.0)	17,809 (13.7)	5994 (15.7)	1700 (15.8)	7530 (15.1)	10,160 (15.7)		
Sleep duration, mean (SD)	7.15 (1.10)	7.14 (1.05)	7.18 (1.22)	7.17 (1.01)	7.14 (1.05)	7.06 (1.16)	7.26 (1.12)	7.23 (1.16)	7.14 (1.27)	< 0.001	< 0.001
Healthy diet, no (%)	334,819 (79.3)	237,607 (80.1)	97,212 (77.3)	102,660 (81.6)	103,151 (79.5)	29,881 (78.1)	8253 (76.7)	38,839 (77.7)	49,796 (77.1)	< 0.001	< 0.001
Physical activity, no (%)										< 0.001	< 0.001
Low	68,756 (14.3)	37,999 (12.82)	22,483 (17.9)	14,150 (11.2)	16,871 (13.0)	6568 (17.2)	1393 (13.0)	7885 (15.8)	13,063 (20.2)		
Middle	151,484 (31.5)	93,945 (31.7)	38,712 (30.8)	40,667 (32.3)	41,311 (31.8)	11,191 (29.2)	3503 (32.6)	16,101 (32.2)	19,020 (29.5)		
High	152,329 (31.6)	100,740 (34.0)	32,834(26.1)	45,418 (36.1)	43,966 (33.9)	10,621 (27.8)	3408 (31.7)	14,292 (28.6)	15,063 (23.3)		
Unknown	109,007 (22.6)	63,811 (21.5)	31,712(25.2)	25,652 (20.4)	27,603 (21.3)	9899 (25.9)	2453 (22.8)	11,692 (23.4)	17,417 (27.0)		
Smoking status, no (%)										< 0.001	< 0.001
Never	262,305 (54.4)	169,873 (57.3)	59,679 (47.5)	462 (0.4)	611 (0.5)	213 (0.6)	60 (0.6)	293 (0.6)	444 (0.7)		
Previous	166,735 (34.6)	95,209 (32.1)	50,860 (40.5)	74,960 (59.6)	72,166 (55.6)	21,315 (55.7)	5401 (50.2)	23,331 (46.7)	30,730 (47.6)		
Current	50,129 (10.5)	30,099 (10.2)	143,98 (11.5)	36,662 (29.1)	44,464 (34.3)	13,511 (35.3)	3643 (33.9)	20,432 (40.9)	26,640 (41.3)		
Unknown	2407 (0.5)	1314 (0.4)	804 (0.6)	13,803 (11.0)	12,510 (9.6)	3240 (8.5)	1653 (15.4)	5914 (11.8)	6749 (10.5)		
Alcohol intake frequency, no (%)										< 0.001	< 0.001
Never	38,189 (7.9)	20,939 (7.1)	12,759 (10.2)	8703 (6.9)	8414 (6.5)	3417 (8.9)	1135 (10.6)	4523 (9.1)	6994 (10.8)		
Special occasion only	55,072 (11.4)	30,774 (10.4)	17,512 (13.9)	12,231 (9.7)	12,556 (9.7)	5594 (14.6)	1299 (12.1)	5717 (11.4)	10,429 (16.2)		
One to three times a month	53,558 (11.1)	31,844 (10.7)	15,012 (11.9)	13,041 (10.4)	13,591 (10.5)	4967 (13.0)	1015 (9.4)	5179 (10.4)	8767 (13.6)		
Once or twice a week	124,319 (25.8)	77,061 (26.0)	31,731 (25.2)	31,890 (25.3)	34,305 (26.4)	10,378 (27.1)	2520 (23.4)	12,593 (25.2)	16,527 (25.6)		
Three or four times a week	111,408 (23.1)	72,376 (24.4)	25,192 (20.0)	31,340 (24.9)	32,747 (25.2)	7829 (20.5)	2250 (20.9)	11,043 (22.1)	11,829 (18.3)		
Daily or almost daily	98,019 (20.4)	62,906 (21.2)	23,232 (18.5)	28,463 (22.6)	27,879 (21.5)	5999 (15.7)	2510 (23.3)	10,823 (21.7)	9842 (15.2)		
Unknown	1011 (0.2)	595 (0.2)	303 (0.2)	219 (0.2)	259 (0.2)	95 (0.3)	28 (0.3)	92 (0.2)	175 (0.3)		
Metabolic risk factors											
BMI, kg/m^2^, mean (SD)	27.47 (4.75)	26.00 (3.92)	30.79 (4.91)	22.81 (1.54)	27.12 (1.37)	33.18 (3.38)	23.52 (1.24)	27.77 (1.38)	34.35 (4.04)	< 0.001	< 0.001
WC, cm, mean (SD)	90.39 (13.37)	85.78 (11.36)	101.13 (11.76)	78.13 (7.92)	89.20 (8.02)	100.39 (10.27)	85.69 (7.09)	95.87 (7.29)	107.77 (10.53)	< 0.001	< 0.001
SBP, mmHg, mean (SD)	137.93 (18.61)	135.13 (18.63)	144.57 (18.63)	132.15 (18.57)	137.33 (18.23)	137.92 (18.58)	144.42 (16.91)	144.89 (16.59)	144.35 (16.90)	< 0.001	< 0.001
DBP, mmHg, mean (SD)	82.30 (10.12)	80.96 (10.01)	85.50 (9.71)	78.52 (9.69)	82.36 (9.74)	84.54 (9.85)	82.45 (9.53)	84.65 (9.50)	86.67 (9.73)	< 0.001	< 0.001
HDL-C, mmoL/L, mean (SD)	1.45 (0.38)	1.55 (0.37)	1.20 (0.33)	1.65 (0.39)	1.48 (0.34)	1.46 (0.31)	1.26 (0.42)	1.21 (0.34)	1.17 (0.31)	< 0.001	< 0.001
TG, mmoL/L, mean (SD)	1.75 (1.03)	1.46 (0.78)	2.43 (1.20)	1.29 (0.66)	1.61 (0.85)	1.51 (0.76)	2.06 (1.11)	2.40 (1.20)	2.51 (1.20)	< 0.001	< 0.001
Serum glucose, mmoL/L, mean (SD)	5.11 (1.18)	2.92 (0.72)	5.58 (1.93)	4.89 (0.76)	4.94 (0.70)	4.97 (0.61)	5.55 (1.99)	5.48 (1.75)	5.66 (2.04)	< 0.001	< 0.001

MH status was defined as < 3 abnormal components

MH, metabolically healthy; MU, metabolically unhealthy; BMI, body mass index; MHN, metabolically healthy normal weight; MHOW, metabolically healthy overweight; MHO, metabolically healthy obesity; MUN, metabolically unhealthy normal weight; MUOW, metabolically unhealthy overweight; MUO, metabolically unhealthy obesity; SD, standard deviation

*P*^a^values: differences between groups MH and MU

*P*^b^values: differences among groups MHN, MHOW, MHO, MUN, MUOW, and MUO

### Combined effects of metabolic status, BMI status, and PRSs on the risk of cardiovascular outcomes and all-cause mortality

**Fig. 2:**
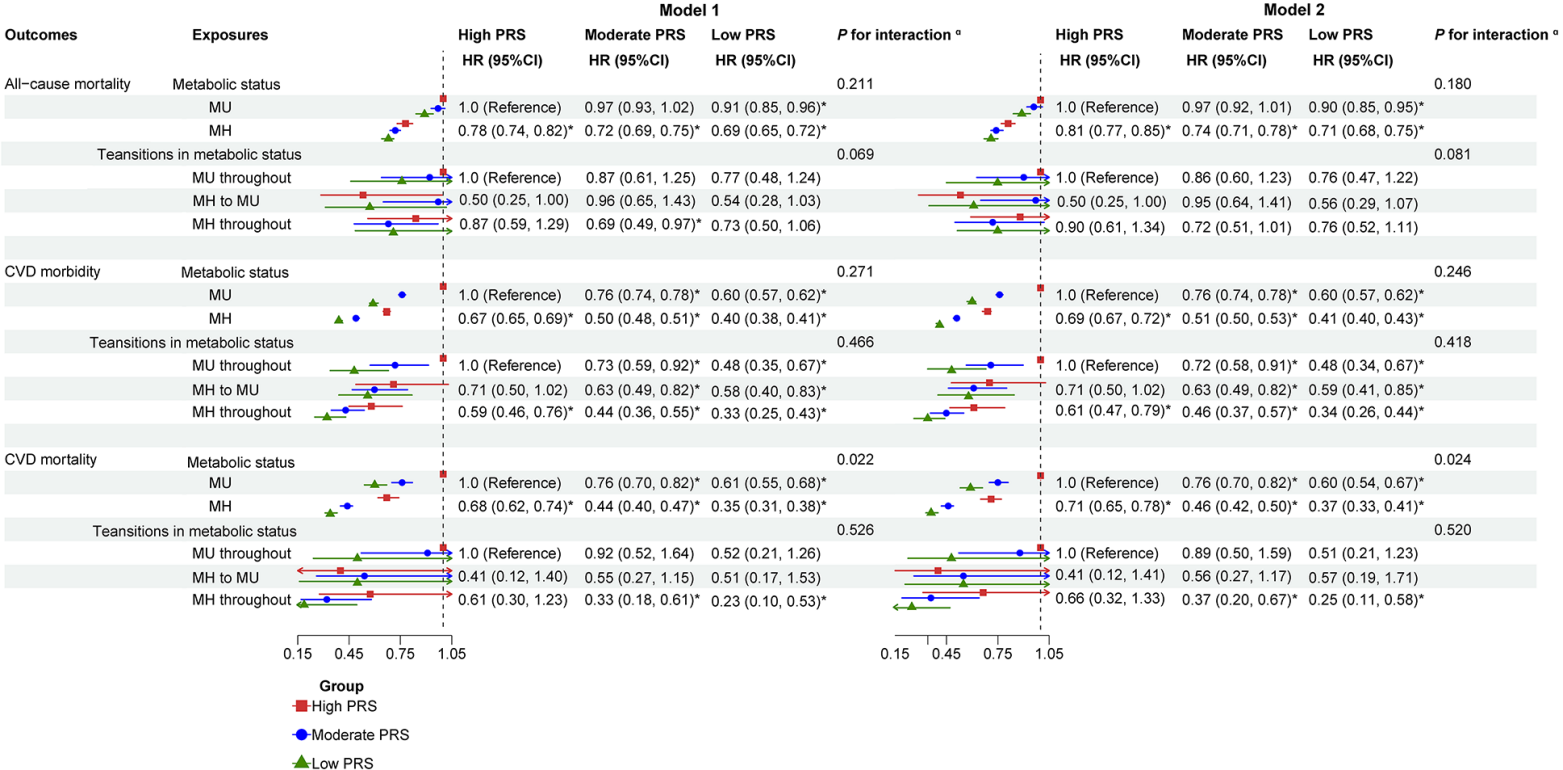
Combined effects of metabolic status, metabolic transitions, and PRSs on the risk of cardiovascular outcomes and all-cause mortality. *Note* MH status was defined as < 3 abnormal components; MH, metabolically healthy; MU, metabolically unhealthy; PRS, polygenic risk score; the PRSs presented are specifically used based on corresponding outcomes; Model 1: adjusted for age, sex, race, Townsend Deprivation Index, annual household income, educational attainment, 22 assessment centers, and the first 5 principal components of ancestry; Model 2: further adjusted for family history of diabetes, family history of high blood pressure, and lifestyle factors including sleep duration, healthy diet, physical activity, smoking status, and alcohol intake frequency based on Model 1; ^a^likelihood tests were applied to test the significance of the interaction term by comparing the model with and without the interaction term; *P < 0.05

**Fig. 3:**
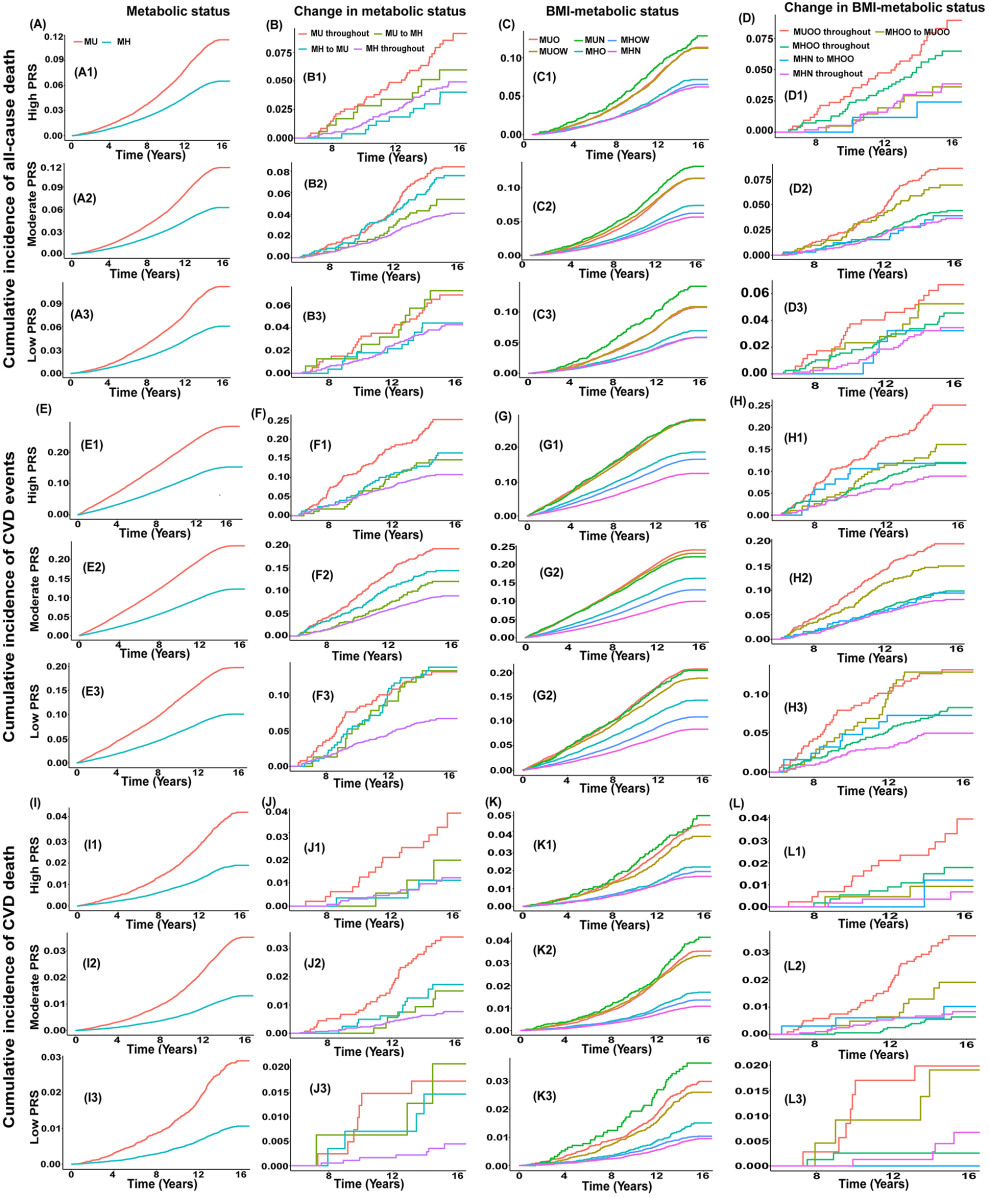
The cumulative incidence of all-cause death, CVD events, and CVD death according to metabolically related status (metabolic status, BMI-metabolic status, and their transitions) and PRSs. *Note* MH was defined as < 3 abnormal components; PRS, polygenic risk score; BMI, body mass index; CVD, cardiovascular disease; MH, metabolically healthy; MU, metabolically unhealthy; MHN, metabolically healthy normal weight; MHOW, metabolically healthy overweight; MHO, metabolically healthy obesity; MUN, metabolically unhealthy normal weight; MUOW, metabolically unhealthy overweight; MUO, metabolically unhealthy obesity

During a median follow-up of 14.38 years (interquartile range, 1.76), all-cause mortality occurred in 31,883 of 438,850 participants (7.3%), CVD mortality in 8133 of 455,548 (1.8%), and CVD morbidity in 67,260 of 455,548 (14.8%) (Table 2). In high PRS group, participants with MH status had a lower risk of all-cause mortality and all cardiovascular outcomes than those with MU status (Tables 2 and S4). Compared with those had a MU status and a high PRS, participants with a MH status and a low PRS had the lowest risk of the aforementioned outcomes (Fig. 2 and Table S5). Furthermore, significant interactions of metabolic status with PRSs on CVD mortality, CAD morbidity, and AF morbidity were identified (*P*_interactions_ < 0.05) (Fig. 2, Tables S4 and S5). In addition, compared to those with an obesity status and a high PRS, individuals with a normal weight status and a low PRS had the lowest risk of cardiovascular morbidity; individuals with an overweight status and a low PRS had the lowest risk of mortality from all-cause, CVD, and HF (Table S6). After excluding one or two abnormal components from current definition of MH status (< 3 abnormal components), the associations of metabolic status (MH status: < 1 and < 2 abnormal components) and PRS with all-cause mortality and all cardiovascular outcomes were not materially altered, but with increased magnitudes (Tables S7 and S8). The cumulative incidence of all-cause death, CVD events, and CVD death for participants with MU status was higher than those with MH status among all PRS groups (*P* < 0.05) (Fig. 3).

**Table 2:** Associations of metabolic status, BMI status, and BMI-metabolic status with the risk of cardiovascular outcomes and all-cause mortality stratified by the levels of PRS

Outcomes	Exposures	No	Case (%)	High PRS	Moderate PRS	Low PRS	*P* for interaction^a^
All-cause mortality	Metabolic status	384,860	28,347 (7.4)				
	MU	105,422	11,616 (11.0)	1.0 (Reference)	1.0 (Reference)	1.0 (Reference)	0.180
	MH	279,438	16,731 (6.0)	0.81 (0.77, 0.86)*	0.77 (0.75, 0.80)*	0.78 (0.74, 0.83)*	
	* P* for trend			< 0.001	< 0.001	< 0.001	
	BMI status	438,850	31,883 (7.3)				
	Obesity	103,689	9573 (9.2)	1.0 (Reference)	1.0 (Reference)	1.0 (Reference)	0.025
	Overweight	187,721	13,439 (7.2)	0.80 (0.75, 0.84)*	0.78 (0.75, 0.80)*	0.78 (0.74, 0.83)*	
	Normal weight	147,440	8871 (6.0)	0.83 (0.78, 0.89)*	0.79 (0.76, 0.82)*	0.87 (0.81, 0.93)*	
	Per 1-point increase			0.91 (0.88, 0.94)*	0.89 (0.87, 0.90)*	0.93 (0.90, 0.97)*	0.010
	* P* for trend			< 0.001	< 0.001	< 0.001	
	BMI-metabolic status	382,162	27,916 (7.3)				
	MUO	54,974	5945 (10.8)	1.0 (Reference)	1.0 (Reference)	1.0 (Reference)	0.042
	MUOW	41,532	4504 (10.8)	0.83 (0.77, 0.91)*	0.85 (0.81, 0.90)*	0.89 (0.81, 0.98)*	
	MUN	8599	1082 (12.6)	0.86 (0.74, 0.99)*	0.92 (0.85, 1.00)	1.08 (0.93, 1.26)	
	MHO	35,596	2474 (7.0)	0.82 (0.74, 0.91)*	0.84 (0.79, 0.89)*	0.85 (0.76, 0.95)*	
	MHOW	122,250	7300 (6.0)	0.70 (0.65, 0.76)*	0.68 (0.65, 0.71)*	0.69 (0.63, 0.75)*	
	MHN	119,211	6611 (5.5)	0.76 (0.70, 0.82)*	0.71 (0.68, 0.74)*	0.80 (0.73, 0.87)*	
	Per 1-point increase			0.944 (0.930, 0.957)*	0.929 (0.922, 0.937)*	0.943 (0.929, 0.957)*	0.024
	* P* for trend			< 0.001	< 0.001	< 0.001	
CVD morbidity	Metabolic status	399,359	59,229 (14.8)				
	MU	110,151	25,123 (22.8)	1.0 (Reference)	1.0 (Reference)	1.0 (Reference)	0.246
	MH	289,208	34,106 (11.8)	0.69 (0.67, 0.72)*	0.67 (0.66, 0.69)	0.70 (0.67, 0.73)	
	* P* for trend			< 0.001	< 0.001	< 0.001	
	BMI status	455,548	67,260 (14.8)				
	Obesity	107,938	21,785 (20.2)	1.0 (Reference)	1.0 (Reference)	1.0 (Reference)	< 0.001
	Overweight	194,735	29,452 (15.1)	0.75 (0.73, 0.78)*	0.70 (0.69, 0.72)*	0.67 (0.64, 0.70)*	
	Normal weight	152,875	16,023 (10.5)	0.62 (0.59, 0.64)*	0.58 (0.57, 0.60)*	0.59 (0.56, 0.62)*	
	Per 1-point increase			0.78 (0.77, 0.80)*	0.76 (0.75, 0.77)*	0.76 (0.75, 0.78)*	0.077
	* P* for trend			< 0.001	< 0.001	< 0.001	
	BMI-metabolic status	396,531	58,817 (14.8)				
	MUO	57,333	13,331 (23.3)	1.0 (Reference)	1.0 (Reference)	1.0 (Reference)	0.035
	MUOW	43,477	9705 (22.3)	0.82 (0.78, 0.86)*	0.80 (0.77, 0.83)*	0.76 (0.71, 0.82)*	
	MUN	9007	1986 (22.0)	0.77 (0.70, 0.84)*	0.71 (0.67, 0.76)*	0.75 (0.67, 0.85)*	
	MHO	36,873	5759 (15.6)	0.80 (0.75, 0.85)*	0.82 (0.79, 0.85)*	0.87 (0.81, 0.94)*	
	MHOW	126,370	16,121 (12.8)	0.64 (0.61, 0.67)*	0.60 (0.58, 0.62)*	0.60 (0.57, 0.64)*	
	MHN	123,471	11,915 (9.7)	0.54 (0.51, 0.57)*	0.52 (0.50, 0.54)*	0.54 (0.51, 0.58)*	
	Per 1-point increase			0.894 (0.887, 0.902)*	0.886 (0.881, 0.891)*	0.893 (0.884, 0.903)*	0.168
	* P* for trend			< 0.001	< 0.001	< 0.001	
CVD mortality	Metabolic status	399,359	7220 (1.8)				
	MU	110,151	3580 (3.3)	1.0 (Reference)	1.0 (Reference)	1.0 (Reference)	0.012
	MH	289,208	3640 (1.3)	0.71 (0.64, 0.78)*	0.61 (0.57, 0.65)*	0.61 (0.54, 0.68)*	
	* P* for trend			< 0.001	< 0.001	< 0.001	
	BMI status	455,548	8133 (1.8)				
	Obesity	107,938	2836 (2.6)	1.0 (Reference)	1.0 (Reference)	1.0 (Reference)	0.352
	Overweight	194,735	3368 (1.7)	0.67 (0.60, 0.73)*	0.68 (0.63, 0.72)*	0.63 (0.55, 0.72)*	
	Normal weight	152,875	1929 (1.3)	0.68 (0.61, 0.77)*	0.63 (0.58, 0.68)*	0.65 (0.56, 0.76)*	
	Per 1-point increase			0.81 (0.76, 0.86)*	0.78 (0.75, 0.81)*	0.80 (0.74, 0.86)*	0.527
	* P* for trend			< 0.001	< 0.001	< 0.001	
	BMI-metabolic status	396,531	7122 (1.8)				0.123
	MUO	57,333	1901 (3.3)	1.0 (Reference)	1.0 (Reference)	1.0 (Reference)	
	MUOW	43,477	1322 (3.0)	0.70 (0.61, 0.81)*	0.78 (0.71, 0.85)*	0.76 (0.63, 0.92)*	
	MUN	9007	337 (3.7)	0.79 (0.63, 0.99)*	0.86 (0.74, 1.00)	0.94 (0.70, 1.27)	
	MHO	36,873	591 (1.6)	0.71 (0.59, 0.85)*	0.68 (0.60, 0.77)*	0.73 (0.58, 0.92)*	
	MHOW	126,370	1643 (1.3)	0.57 (0.50, 0.64)*	0.52 (0.47, 0.56)*	0.49 (0.41, 0.58)*	
	MHN	123,471	1328 (1.1)	0.60 (0.52, 0.69)*	0.50 (0.46, 0.55)*	0.54 (0.45, 0.64)*	
	Per 1-point increase			0.90 (0.88, 0.93)*	0.87 (0.85, 0.88)*	0.87 (0.85, 0.90)*	0.015
	* P* for trend			< 0.001	< 0.001	< 0.001	

BMI, body mass index; MH status was defined as < 3 abnormal components; MH, metabolically healthy; MU, metabolically unhealthy; MHN, metabolically healthy normal weight; MHOW, metabolically healthy overweight; MHO, metabolically healthy obesity; MUN, metabolically unhealthy normal weight; MUOW, metabolically unhealthy overweight; MUO, metabolically unhealthy obesity; PRS, polygenic risk score; the PRSs presented are specifically used based on corresponding outcomes; each model was adjusted for age, sex, race, Townsend Deprivation Index, annual household income, education attainment, 22 assessment centers, the first 5 principal components of ancestry, family history of diabetes, family history of high blood pressure, and lifestyle factors including sleep duration, healthy diet, physical activity, smoking status, and alcohol intake frequency

^a^Likelihood tests were applied to test the significance of the interaction term by comparing the model with and without the interaction term

*P < 0.05

### Combined effects of transitions in metabolic status, BMI status, and PRSs on the risk of cardiovascular outcomes and all-cause mortality

In the second survey (2012–2013), we recorded the metabolic transitions of 13,216 participants and BMI transitions of 19,922 participants. Among participants with MH status at baseline, 85.0% maintained their original status, while 15.0% converted to MU status (Table S9). For participants with normal weight, 84.7% maintained their original status, while 15.2% converted to overweight (Table S10). The median follow-up period for outcomes beginning with the second survey was 10.65 years (interquartile range: 0.48 years).

Compared with those had a consistently MU status and a high PRS, participants with a consistently MH status and a low PRS experienced the lowest risk of CVD morbidity (Model 2: HR 0.34; 95% CI 0.26, 0.44), CVD mortality (Model 2: HR 0.25; 95% CI 0.11, 0.58) (Fig. 2), and the risk of partial specific cardiovascular outcomes, including the morbidity of CAD, MI, stroke, and AF (Table S11). Moreover, the significant interactions between metabolic transitions and PRSs on HF mortality and AF mortality were identified (Model 2: *P*_interaction_ < 0.05) (Table S11). For the transitions in BMI status, compared with those with an obesity throughout status and a high PRS, individuals with a normal weight throughout status and a low PRS had the lowest risk of CVD morbidity (Table S12).

As depicted in Fig. 3, in the high and moderate PRS groups, the cumulative incidence of all-cause death, CVD events, and CVD death was highest among participants with stable MU status (*P* < 0.05).

### Combined effects of BMI-metabolic status and PRSs on risk of cardiovascular outcomes and all-cause mortality

**Fig. 4:**
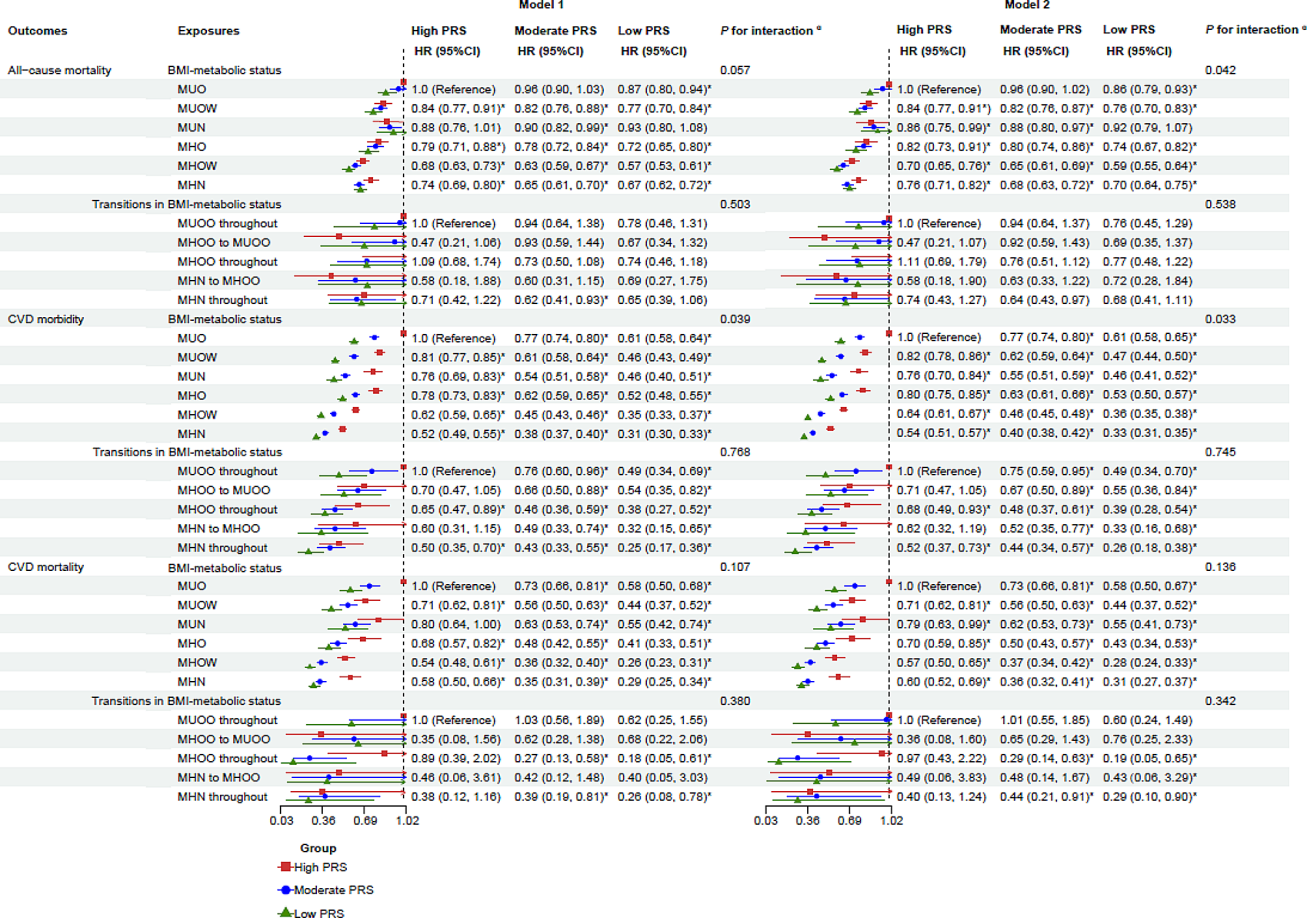
Combined effects of BMI-metabolic status, transitions in BMI-metabolic status, and PRSs on the risk of cardiovascular outcomes and all-cause mortality. *Note* Metabolically healthy status was defined as < 3 abnormal components; BMI, body mass index; MHN, metabolically healthy normal weight; MHOW, metabolically healthy overweight; MHO, metabolically healthy obesity; MUN, metabolically unhealthy normal weight; MUOW, metabolically unhealthy overweight; MUO, metabolically unhealthy obesity; PRS, polygenic risk score; the PRSs presented are specifically used based on corresponding outcomes; Model 1: adjusted for age, sex, race, Townsend Deprivation Index, annual household income, education attainment, 22 assessment centers, and the first 5 principal components of ancestry; Model 2: further adjusted for family history of diabetes, family history of high blood pressure, and lifestyle factors including sleep duration, healthy diet, physical activity, smoking status, and alcohol intake frequency based on Model 1; ^a^likelihood tests were applied to test the significance of interaction term by comparing the model with and without the interaction term; *P < 0.05

The joint associations of BMI-metabolic status and PRSs with each outcome were illustrated in Fig. 4 and Table S13. Compared to MUO status and high PRSs, participants with a MHN status and a low PRS exhibited the lowest risk of CVD morbidity (Model 2: HR 0.33; 95% CI 0.31, 0.35) and specific cardiovascular outcomes, including morbidity from CAD, MI, stroke, and HF, as well as mortality from MI and AF. Individuals with a MHOW status and a low PRS experienced the lowest risk of mortality from all-cause (Model 2: HR 0.59; 95% CI 0.55, 0.64), CVD (Model 2: HR 0.28; 95% CI 0.24, 0.33), CAD, and HF.

Among those with a high PRS, participants with a MHOW status had the lowest risk of mortality from all-cause (HR 0.70; 95% CI 0.65, 0.76), CVD (HR 0.57; 95% CI 0.50, 0.64), HF, and AF; participants with a MHN status had the lowest risk of CVD morbidity (HR 0.54; 95% CI 0.51, 0.57), CAD morbidity, CAD mortality, MI morbidity, MI mortality, HF morbidity, and AF morbidity (Tables 2 and S4).

The inverse associations of BMI-metabolic status with CVD mortality (HRs [95% CI] in ascending PRS levels: 0.87 [0.85, 0.90], and 0.87 [0.85, 0.88], 0.90 [0.88, 0.93], respectively), CAD morbidity, and AF morbidity appeared to be greater among individuals with moderate and low PRS levels (Tables 2 and S4). The significant interactions of BMI-metabolic status with PRSs on the all-cause mortality, CVD morbidity, CVD mortality, CAD morbidity, and AF morbidity were identified (*P*_interactions_ < 0.05) (Fig. 4, Tables 2, S4, and S13). Additionally, the combined effects of BMI-metabolic status (MH status: < 1 and < 2 abnormal components) and PRS on all-cause mortality and risk of all cardiovascular outcomes showed no substantial modification (Tables S14 and S15).

In terms of the cumulative incidence of all-cause death, participants with MUO, MUOW, and MUN phenotypes exhibited higher rates than those with MHO, MHOW, and MHN phenotypes across all PRS groups (*P* < 0.05). Similar trends were observed in the cumulative incidence of CVD events and CVD death (Fig. 3).

### Combined effects of transitions in BMI-metabolic status and PRS on the risk of cardiovascular outcomes and all-cause mortality

Furthermore, information regarding the transitions of BMI-metabolic status was examined for 13,093 participants during the follow-up period (2012–2013). At the second survey, the majority of individuals with MHN status (80.4%) maintained their original status. For participants with MHOO status, 68.2% did not undergo conversion, while 21.4% transitioned to MUOO in the resurvey. The proportions of participants with MUN status transitioning to MHN, MHOO, and MUOO were 28.9%, 6.6%, and 17.6%, respectively. Among participants with MUOO status at baseline, the proportions who transitioned to MHN, MHOO, and MUN were 2.4%, 22.7%, and 2.4%, respectively (Table S16).

As presented in Fig. 4 and Table S17, compared to those with a stable MUO status and a high PRS, participants with a stable MHN status and a low PRS manifested the lowest risk of CVD morbidity (Model 2: HR 0.26; 95% CI 0.18, 0.38) and specific cardiovascular outcomes, including CAD morbidity, MI morbidity, and AF morbidity. Participants with stable MHOO status and low PRS had the lowest risk of CVD mortality (Model 2: HR 0.19; 95% CI 0.05, 0.65) and stroke mortality. Furthermore, participants with stable MUOO status had the highest cumulative incidence of all-cause deaths, CVD events, and CVD deaths among all PRS groups (*P* < 0.05, Fig. 3).

### Sensitivity analysis

As shown in Tables S18–S20, excluding CVD cases or deaths that occurred within the first two years of follow-up did not materially alter the results for all-cause mortality, CVD morbidity, and CVD mortality. Due to a limited number of events, individuals of non-White ethnicity were not included in the subgroup analyses. Nonetheless, the main findings for the White ethnic group remained largely unchanged. Furthermore, the beneficial associations of metabolic status, BMI status, BMI-metabolic status, and PRSs with all-cause mortality, CVD morbidity, and CVD mortality appeared to be greater among younger individuals (< 65 years, *P*_interaction_ < 0.001) (Table 3). In terms of sex-stratified analysis, significant differences were observed in the impact of metabolic status and PRSs on all-cause mortality and CVD morbidity (*P*_interaction_ < 0.001) (Tables S18 and S19). The Wald test results suggested that the potential survival bias introduced by including only participants who survived to the second survey does not significantly influence our primary conclusions (Table S21). In addition, we found a significant interaction between lifestyle factors and genetic risk for CAD and stroke (Table S22). Given that no substantial difference was found between Model 1 (unadjusted lifestyle factors) and Model 2 (adjusted lifestyle factors), the complex interplay of genetic risk and lifestyle factors might not substantially affect our primary results.

**Table 3:** Associations of metabolic status, BMI status, BMI-metabolic status, and PRS with the risk of cardiovascular outcomes and all-cause mortality stratified by age group

Subgroup	Exposures	No	Case (%)	High PRS	Moderate PRS	Low PRS	*P* for interaction ^a^
All-cause mortality	Metabolic status						< 0.001
≥ 65 Years	MU	25,137	5096 (20.3)	1.0 (Reference)	0.97 (0.91, 1.04)	0.87 (0.79, 0.95)*	
	MH	42,410	6133 (14.5)	0.85 (0.78, 0.93)*	0.77 (0.72, 0.82)*	0.74 (0.68, 0.80)*	
< 65 Years	MU	80,285	6520 (8.1)	1.0 (Reference)	0.99 (0.93, 1.05)	0.97 (0.90, 1.05)	
	MH	237,028	10,598 (4.5)	0.67 (0.62, 0.71)*	0.63 (0.60, 0.67)*	0.61 (0.57, 0.66)*	
	BMI status						< 0.001
≥ 65 Years	Obesity	18,000	3627 (20.2)	1.0 (Reference)	0.90 (0.83, 0.98)*	0.79 (0.72, 0.88)*	
	Overweight	35,809	5643 (15.8)	0.77 (0.70, 0.84)*	0.73 (0.67, 0.79)*	0.66 (0.60, 0.72)*	
	Normal weight	22,999	3404 (14.8)	0.81 (0.73, 0.90)*	0.73 (0.67, 0.80)*	0.75 (0.68, 0.83)*	
< 65 Years	Obesity	85,689	5946 (6.9)	1.0 (Reference)	1.01 (0.95, 1.08)	0.96 (0.88, 1.04)	
	Overweight	151,912	7796 (5.1)	0.81 (0.75, 0.87)*	0.77 (0.72, 0.82)*	0.71 (0.66, 0.77)*	
	Normal weight	124,441	5467 (4.4)	0.79 (0.73, 0.86)*	0.73 (0.68, 0.78)*	0.73 (0.68, 0.79)*	
	BMI-metabolic status						< 0.001
≥ 65 Years	MUO	10,784	2347 (21.8)	1.0 (Reference)	0.94 (0.85, 1.05)	0.81 (0.71, 0.92)	
	MUOW	11,507	2175 (18.9)	0.84 (0.73, 0.95)*	0.81 (0.73, 0.90)*	0.74 (0.65, 0.85)*	
	MUN	2779	549 (19.8)	0.78 (0.63, 0.97)*	0.87 (0.76, 1.00)	0.85 (0.69, 1.05)	
	MHO	5025	865 (17.2)	0.96 (0.81, 1.14)	0.79 (0.70, 0.90)*	0.76 (0.63, 0.90)*	
	MHOW	19,777	2754 (13.9)	0.70 (0.61, 0.79)*	0.65 (0.59, 0.72)*	0.61 (0.54, 0.68)*	
	MHN	17,230	2396 (13.9)	0.79 (0.69, 0.89)*	0.69 (0.62, 0.76)*	0.71 (0.63, 0.80)*	
< 65 Years	MUO	44,190	3598 (8.1)	1.0 (Reference)	1.00 (0.92, 1.08)	0.95 (0.85, 1.06)	
	MUOW	30,025	2329 (7.8)	0.94 (0.84, 1.05)	0.94 (0.86, 1.03)	0.92 (0.81, 1.03)	
	MUN	5820	533 (9.2)	1.12 (0.93, 1.35)	1.09 (0.96, 1.25)	1.24 (1.00, 1.53)	
	MHO	30,571	1609 (5.3)	0.68 (0.60, 0.78)*	0.74 (0.68, 0.81)*	0.70 (0.61, 0.79)*	
	MHOW	102,473	4546 (4.4)	0.64 (0.58, 0.71)*	0.60 (0.56, 0.65)*	0.55 (0.50, 0.61)*	
	MHN	101,981	4215 (4.1)	0.66 (0.60, 0.73)*	0.60 (0.55, 0.65)*	0.62 (0.56, 0.68)*	
CVD morbidity	Metabolic status						< 0.001
≥ 65 Years	MU	26,878	9332 (34.7)	1.0 (Reference)	0.80 (0.76, 0.84)*	0.65 (0.61, 0.70)*	
	MH	45,096	10,805 (24.0)	0.75 (0.70, 0.79)*	0.58 (0.55, 0.61)*	0.48 (0.45, 0.51)*	
< 65 Years	MU	83,273	15,791 (19.0)	1.0 (Reference)	0.77 (0.74, 0.80)*	0.60 (0.57, 0.63)*	
	MH	244,112	23,301 (9.5)	0.57 (0.55, 0.60)*	0.43 (0.41, 0.44)*	0.35 (0.33, 0.36)*	
	BMI status						< 0.001
≥ 65 Years	Obesity	19,239	6837 (35.5)	1.0 (Reference)	0.80 (0.76, 0.85)*	0.67 (0.62, 0.72)*	
	Overweight	38,113	10,577 (27.8)	0.77 (0.72, 0.82)*	0.59 (0.56, 0.63)*	0.47 (0.44, 0.50)*	
	Normal weight	24,485	5459 (22.3)	0.65 (0.60, 0.70)*	0.50 (0.47, 0.53)*	0.40 (0.37, 0.44)*	
< 65 Years	Obesity	88,699	14,948 (16.9)	1.0 (Reference)	0.79 (0.76, 0.82)*	0.64 (0.61, 0.68)*	
	Overweight	156,622	18,875 (12.1)	0.75 (0.72, 0.79)*	0.55 (0.53, 0.57)*	0.41 (0.39, 0.43)*	
	Normal weight	128,390	10,564 (8.2)	0.58 (0.55, 0.61)*	0.42 (0.41, 0.44)*	0.35 (0.33, 0.37)*	
	BMI-metabolic status						< 0.001
≥ 65 Years	MUO	11,553	4362 (37.8)	1.0 (Reference)	0.80 (0.74, 0.86)*	0.67 (0.61, 0.73)*	
	MUOW	12,297	4043 (32.9)	0.82 (0.75, 0.90)*	0.65 (0.61, 0.70)*	0.52 (0.47, 0.57)*	
	MUN	2954	894 (30.3)	0.71 (0.61, 0.82)*	0.59 (0.53, 0.66)*	0.46 (0.39, 0.55)*	
	MHO	5329	1660 (31.2)	0.87 (0.77, 0.99)*	0.69 (0.63, 0.75)*	0.60 (0.53, 0.68)*	
	MHOW	20,998	5177 (24.7)	0.65 (0.59, 0.71)*	0.51 (0.47, 0.55)*	0.42 (0.38, 0.45)*	
	MHN	18,361	3870 (21.1)	0.60 (0.54, 0.65)*	0.46 (0.42, 0.49)*	0.37 (0.33, 0.41)*	
< 65 Years	MUO	45,780	8969 (19.6)	1.0 (Reference)	0.79 (0.75, 0.83)*	0.63 (0.59, 0.67)*	
	MUOW	31,180	5662 (18.2)	0.92 (0.86, 0.98)*	0.70 (0.66, 0.73)*	0.51 (0.47, 0.56)*	
	MUN	6053	1092 (18.0)	0.98 (0.87, 1.09)	0.66 (0.60, 0.72)*	0.60 (0.51, 0.70)*	
	MHO	31,544	4099 (13.0)	0.70 (0.65, 0.75)*	0.57 (0.54, 0.60)*	0.48 (0.44, 0.52)*	
	MHOW	105,372	10,944 (10.4)	0.58 (0.55, 0.62)*	0.42 (0.40, 0.44)*	0.33 (0.31, 0.35)*	
	MHN	105,110	8045 (7.7)	0.47 (0.44, 0.50)*	0.35 (0.33, 0.37)*	0.29 (0.27, 0.31)*	
CVD mortality	Metabolic status						< 0.001
≥ 65 Years	MU	26,878	1677 (6.2)	1.0 (Reference)	0.78 (0.69, 0.87)*	0.56 (0.48, 0.65)*	
	MH	45,096	1571 (3.5)	0.78 (0.68, 0.90)*	0.49 (0.43, 0.55)*	0.42 (0.36, 0.49)*	
< 65 Years	MU	83,273	1903 (2.3)	1.0 (Reference)	0.77 (0.69, 0.85)*	0.69 (0.60, 0.80)*	
	MH	244,112	2069 (0.8)	0.54 (0.48, 0.61)*	0.37 (0.33, 0.41)*	0.28 (0.24, 0.32)*	
	BMI status						0.376
≥ 65 Years	Obesity	19,239	1208 (6.3)	1.0 (Reference)	0.71 (0.62, 0.82)*	0.54 (0.45, 0.65)*	
	Overweight	38,113	1619 (4.2)	0.71 (0.61, 0.82)*	0.50 (0.44, 0.57)*	0.37 (0.31, 0.44)*	
	Normal weight	24,485	843 (3.4)	0.69 (0.58, 0.82)*	0.44 (0.38, 0.51)*	0.38 (0.31, 0.47)*	
< 65 Years	Obesity	88,699	1628 (1.8)	1.0 (Reference)	0.72 (0.64, 0.80)*	0.60 (0.51, 0.70)*	
	Overweight	156,622	1749 (1.1)	0.64 (0.56, 0.73)*	0.47 (0.42, 0.53)*	0.34 (0.29, 0.39)*	
	Normal weight	128,390	1086 (0.8)	0.63 (0.55, 0.73)*	0.42 (0.37, 0.47)*	0.34 (0.28, 0.40)*	
	BMI-metabolic status						< 0.001
≥ 65 Years	MUO	11,553	824 (7.1)	1.0 (Reference)	0.80 (0.74, 0.86)*	0.67 (0.61, 0.73)*	
	MUOW	12,297	691 (5.6)	0.82 (0.75, 0.90)*	0.65 (0.61, 0.70)*	0.52 (0.47, 0.57)*	
	MUN	2954	155 (5.2)	0.71 (0.61, 0.82)*	0.59 (0.53, 0.66)*	0.46 (0.39, 0.55)*	
	MHO	5329	242 (4.5)	0.87 (0.77, 0.99)*	0.69 (0.63, 0.75)*	0.60 (0.53, 0.68)*	
	MHOW	20,998	716 (3.4)	0.65 (0.59, 0.71)*	0.51 (0.47, 0.55)*	0.42 (0.38, 0.45)*	
	MHN	18,361	576 (3.1)	0.60 (0.54, 0.65)*	0.46 (0.42, 0.49)*	0.37 (0.33, 0.41)*	
< 65 Years	MUO	45,780	1077 (2.4)	1.0 (Reference)	0.73 (0.64, 0.84)*	0.68 (0.56, 0.82)*	
	MUOW	31,180	631 (2.0)	0.76 (0.63, 0.92)*	0.65 (0.56, 0.76)*	0.53 (0.42, 0.68)*	
	MUN	6053	182 (3.0)	1.22 (0.92, 1.63)	0.90 (0.72, 1.13)	0.87 (0.59, 1.29)	
	MHO	31,544	349 (1.1)	0.58 (0.46, 0.74)*	0.44 (0.37, 0.53)*	0.33 (0.25, 0.45)*	
	MHOW	105,372	927 (0.9)	0.49 (0.41, 0.58)*	0.33 (0.29, 0.38)*	0.24 (0.19, 0.29)*	
	MHN	105,110	752 (0.7)	0.47 (0.39, 0.57)*	0.31 (0.27, 0.36)*	0.26 (0.21, 0.32)*	

BMI, body mass index; MH status was defined as < 3 abnormal components; MH, metabolically healthy; MU, metabolically unhealthy; MHN, metabolically healthy normal weight; MHOW, metabolically healthy overweight; MHO, metabolically healthy obesity; MUN, metabolically unhealthy normal weight; MUOW, metabolically unhealthy overweight; MUO, metabolically unhealthy obesity; PRS, polygenic risk score; the PRSs presented are specifically used based on corresponding outcomes; each model was adjusted for age, sex, race, Townsend Deprivation Index, annual household income, education attainment, 22 assessment centers, the first 5 principal components of ancestry, family history of diabetes, family history of high blood pressure, and lifestyle factors including sleep duration, healthy diet, physical activity, smoking status, and alcohol intake frequency

^a^Likelihood tests were applied to test the significance of the interaction term by comparing the model with and without the interaction term

*P < 0.05

## Discussion

In a prospective cohort study during a median follow-up of 14.38 years, we revealed that participants with a MH status and a low PRS were associated with a reduced risk of all-cause mortality and adverse cardiovascular outcomes across all BMI categories. These protective associations were more pronounced among younger participants (< 65 years). Additionally, compared to those with a stable MU status and a high PRS, individuals with a stable MH status and a low PRS exhibited the lowest risk for these outcomes, which remained consistent across BMI categories. These findings emphasize the importance of improving metabolic heath across all BMI categories and PRS levels, especially for individuals younger than 65 years.

MetS is not a singular disease but rather a constellation of CVD risk factors, including obesity, hypertension, hyperglycemia, and dyslipidemia [24]. The association between MetS and CVD has been well described in previous studies, consistently reporting that a higher number of metabolic risk factors is associated with an increased risk of cardiovascular events [25–28]. Recently, numerous cohort studies published in 2023 reported that the associations of ideal cardiovascular health factors (i.e., BMI, blood lipids, blood glucose, and blood pressure) and low PRS with reduced risk of cardiovascular morbidity and mortality [13, 14]. Corroborating these observations, our study yielded similar results. However, MetS is not a stable state, emphasizing the importance of analyzing the combined effects of transitions in metabolic status and PRSs on clinical outcomes. In a prospective cohort study, evidence indicated that improving cardiovascular health status plays a role in reducing cardiovascular risk [29]. To our knowledge, this study is the first to report that individuals with a consistently MH status and a low PRS experienced the lowest risk of cardiovascular outcomes, including CVD mortality and morbidity from CVD, CAD, MI, stroke, and AF.

With regard to BMI-metabolic status, an accumulating body of research aims to investigate its association with CVD-related morbidity and mortality [19, 30–33]. One cohort study investigating the relationship between BMI-metabolic status and the incidence of cardiovascular events revealed that MHN individuals had a lower risk of CVD, CAD, and MI than MUO individuals [19]. Two systematic review and meta-analyses involving 22 prospective studies published in 2016 indicated that, compared with MHN individuals, MHO individuals had greater risk of CVD incidence but not all-cause mortality [30, 31]. Subsequently, several meta-analyses including more than 43 original studies reported that MHO individuals experienced an elevated risk of cardiovascular events and all-cause mortality compared with individuals in the MHN reference group [32, 33]. In the aforementioned findings, the role of genetic stratification has not been considered. Our present observations provide evidence that participants with a MHN status and a low PRS had the lowest risk of cardiovascular outcomes, encompassing morbidity from CVD, CAD, MI, stroke, and HF, along with mortality from MI and AF. Further analysis identified that those with a MHOW status and a low PRS had the lowest risk of all-cause mortality, as well as mortality specifically from CVD, CAD, and HF. Recent studies investigating the associations of cardiovascular health factors and PRS with CVD risk have shown comparable results [13, 14]. Additionally, our results indicated a significant interaction between BMI-metabolic status and genetic susceptibility to CVD. The observed beneficial effects of BMI-metabolic status on all-cause mortality and CVD mortality were more pronounced among individuals with moderate or low PRSs than among those with high PRSs. This finding aligns with prior research that revealed a significant interaction between Life’s Essential 8 and genetic susceptibility to CAD, showing stronger protective associations among individuals with a lower genetic risk of CAD [14]. These findings suggest that genetic risk data for CVD can be utilized to optimize healthcare resource allocation and to formulate precise public health strategies and personalized interventions. For example, more medical resources can be directed towards the management of individuals at high genetic risk for CVD. For those with moderate or low genetic risk, the focus should be on maintaining and improving metabolic health across BMI categories and implementing appropriate prevention measures.

Proof confirmed that MHO is transient, underscoring the necessity of investigating how its transitional patterns influence the incidence of cardiovascular events and all-cause mortality [6, 34]. Consistent with our findings, the MESA and ATTICA studies reported that the shift from MHO to MUO is associated with a heightened risk of CVD in comparison to a sustained MHN reference group [34, 35]. Additionally, one large prospective cohort study with a median follow-up of 24 years, conducted among Western female nurses, showed that individuals with consistently MU had an increased risk of CVD compared to those with a stable MHN across all BMI categories [6]. However, the associations of the transitions in BMI-metabolic phenotypes and a PRS with the risk of CVD and all-cause mortality remain unclear. In the present findings, we concluded that, compared to those with the combined phenotypes of MUO throughout and high PRS, individuals with a stable MHN status and a low PRS had the lowest risk of morbidity from CVD, CAD, MI, and AF. Moreover, individuals with a stable MHOO status and a low PRS exhibited the lowest risk of CVD mortality and stroke mortality. Maintaining MH is challenging for individuals who are obese or overweight, yet it is essential for preventing CVD-related mortality. These findings can assist health policymakers in planning evidence-based interventions for CVD by maintaining MH across all BMI categories and PRS levels.

Sex differences in CVD and all-cause mortality have been extensively documented [36, 37]. It has been reported that men exhibited higher all-cause mortality (12.4%) compared to women (7.7%) (*P* = 0.005) [36]. One cohort study, with a median follow-up of 16.69 years and involving 21 countries, identified that women exhibited a significantly lower cardiovascular risk than men, particularly in the younger population [37]. Remarkably, we found that females at a high PRS displayed stronger protective associations between metabolic status and all-cause mortality. Regarding age, the Women’s Health Study reported that the association between most risk factors and CAD attenuated as the age at onset increases [38]. Similarly, several prior cohort studies have demonstrated that the inverse associations of ideal cardiovascular health factors with all-cause mortality and CVD morbidity were stronger in younger individuals [14, 39]. Reflecting these findings, our results showed that the inverse associations of metabolic status, BMI status, BMI-metabolic status, and PRS with cardiovascular outcomes and all-cause mortality were more prominent among younger individuals, underscoring the importance of improving metabolic factors earlier in life. This evidence highlights the need for recommendations to enhance the identification and management of established or emerging metabolic risk factors in young individuals across all BMI categories and PRS levels.

## Limitation

There are several limitations that need to be considered. First, given the observational nature of this study, establishing a causal association is not feasible. Second, data on covariates, including physical activity, smoking status, sleep duration, and dietary habits, were self-reported, which may introduce measurement bias. Third, the limited sample size in certain categories during the second survey could potentially impact the stability of our results. Fourth, despite adjusting for known confounders and omitting individuals with a history of CVD and cancer at baseline, the presence of unmeasured confounding variables and the possibility of reverse causality cannot be ruled out. Fifth, while our study has identified significant associations between metabolic changes and CVD outcomes, further exploration of metabolic transitions over an extended duration may provide deeper insights. Sixth, differences in genetic ancestry among study participants can lead to population stratification, potentially biasing the associations between PRSs and corresponding outcomes [40]. Although we adjusted for principal components of ancestry in our analysis, residual confounding may still influence our results. Finally, given that the majority of participants in our study, including those for whom the PRSs were calculated, were of white British ethnicity, extending the generalizability of our findings to other racial or ethnic groups remains uncertain.

## Conclusion

In summary, our findings indicated that MH status and low PRS were linked to a reduced risk of adverse cardiovascular outcomes and all-cause mortality across all BMI categories, with individuals younger than 65 years experiencing a greater protective association. These findings provide directions for clinical practice guidelines in formulating precise public health strategies and personalized interventions. Further research is required to validate these observations in diverse populations and to explore the underlying mechanisms involved.

### Electronic supplementary material

Below is the link to the electronic supplementary material.

Supplementary Material 1.

## Data Availability

The dataset supporting the conclusions of this article is available in the public UK Biobank Resource (www.ukbiobank.ac.uk/).

## Abbreviations

AF: Atrial fibrillation
BMI: Body mass index
CAD: Coronary disease
CIs: Confidence intervals
CVD: Cardiovascular disease
DBP: Diastolic blood pressure
HDL-C: High-density lipoprotein cholesterol
HF: Heart failure
HRs: Hazard ratios
MetS: Metabolic syndrome
MH: Metabolically healthy
MHO: Metabolically healthy obesity
MHOW: Metabolically healthy overweight
MI: Myocardial infarction
MU: Metabolically unhealthy
MUN: Metabolically unhealthy normal weight
MUO: Metabolically unhealthy obesity
MUOW: Metabolically unhealthy overweight
PRSs: Polygenic risk scores
SBP: Systolic blood pressure
SD: Standard deviation
TG: Triglyceride
