# Supplementary material for: Associations of metabolic changes and polygenic risk scores with cardiovascular outcomes and all-cause mortality across BMI categories: a prospective cohort study
Source: Cardiovasc Diabetol. 2024 Jul 4;23:231. doi: 10.1186/s12933-024-02332-w (PMC11225301; doi:10.1186/s12933-024-02332-w)
Supplement: Supplementary file 1 — Supplementary Material 1. [file 12933_2024_2332_MOESM1_ESM.docx]

**CONTENT**

Supplementary Method………………………………………………………………………………………………………………………………………………………………………………………………………………………………………...…………….1

Table S1 31 Single nucleotide polymorphisms associated with myocardial infarction………………………………………………………………………………………………………………………………………………………………….………………….2

Table S2 32 Single nucleotide polymorphisms associated with stroke…………………………………………………………………………………………………………………………………………………………………………………………...…………….3

Table S3 12 Single nucleotide polymorphisms associated with heart failure…………………………………………………………………………………………………………………………………………………………………………………………………...4

Table S4 Associations of metabolic status, BMI status, and BMI-metabolic status with the risk of specific cardiovascular outcomes stratified by the levels of PRSs…………………………………………………………...…………………...…………………................5

Table S5 Combined effects of metabolic status (MH status: < 3 abnormal components) and PRSs on the risk of the specific cardiovascular outcomes………………………………………………………………………………………...…………………….................11

Table S6 Combined effects of BMI status and PRSs on the risk of the cardiovascular outcomes and all-cause mortality………………………………………………………………………...………………………………………………………………………………12

Table S7 Combined effects of metabolic status (MH status: < 2 abnormal components) and PRSs on the risk of cardiovascular outcomes and all-cause mortality………………………………………………………………………...……………………………………14

Table S8 Combined effects of metabolic status (MH status: < 1 abnormal components) and PRSs on the risk of cardiovascular outcomes and all-cause mortality……………………………………………………………………………………………………………...16

Table S9 Transitions of metabolic status (MH status: < 3 abnormal components) from baseline to the second resurvey…………………………………...…………………………………………………………………………………………………………...............18

Table S10 Transitions of BMI status from baseline to the second resurvey…………………………………...…………………………………………………………………………………………………………............... …………………………………….........19

Table S11 Combined effects of transitions in metabolic status (MH status: < 3 abnormal components) and PRSs on the risk of specific cardiovascular outcomes……………………………………………………………………………………………………………...20

Table S12 Combined effects of transitions in BMI status and PRSs on the risk of cardiovascular outcomes and all-cause mortality……………………………………………………………………………………………………………. ……………. .………………22

Table S13 Combined effects of BMI-metabolic status (MH status: < 3 abnormal components) and PRSs on the risk of the specific cardiovascular outcomes…………………………………………………………………...…………………………………………...…25

Table S14 Combined effects of BMI-metabolic status (MH status: < 2 abnormal components) and PRSs on the risk of cardiovascular outcomes and all-cause mortality……………………………………………………...………………………………………....…....28

Table S15 Combined effects of BMI-metabolic status (MH status: < 1 abnormal component) and PRSs on the risk of cardiovascular outcomes and all-cause mortality…………………………………………….………...…………………………………….………....31

Table S16 Transitions of BMI-metabolic status (MH status: < 3 abnormal components) from baseline to the second resurvey…………………………...……………………………………………………………………………………………………..…………….34

Table S17 Combined effects of transitions in BMI-metabolic status (MH status: < 3 abnormal components) and PRSs on the risk of specific cardiovascular outcomes…………………………………………………………………………………………………...……35

Table S18 Associations of metabolic status, BMI status, BMI-metabolic status, and PRSs with the risk of all-cause mortality stratified by potential risk factors………………………………………………………………………………………………………………..37

Table S19 Associations of metabolic status, BMI status, BMI-metabolic status, and PRSs with the risk of CVD morbidity stratified by potential risk factors…………………………………………………………………………………………………………………..39

Table S20 Associations of metabolic status, BMI status, BMI-metabolic status, and PRSs with the risk of CVD mortality stratified by potential risk factors…………………………………………………………………………………………………………………....41

Table S21 Associations of baseline exposures (metabolic status, BMI status, BMI-metabolic status), PRSs and CVD morbidity, including participants who participated in the second survey and who died between baseline and the second survey………..…43

Table S22 Associations of lifestyles and PRSs with all-cause mortality and cardiovascular outcomes…………………………………………………………………………………………………………………... …...…………………………………………45

**Supplementary Method**

To address the survival bias introduced by including only participants who survived the second survey in our study, we conducted Wald tests comparing the coefficients of baseline exposures between two Cox proportional hazards models: Model A and Model B. Both Model A and Model B analysed the impact of baseline exposures, including metabolic status, BMI status, and BMI-metabolic status, on CVD morbidity. Model A included participants who participated in the second survey, while Model B included participants who participated in the second survey and who died between baseline and the second survey. Furthermore, the interaction and joint associations of lifestyle factors and genetic predisposition with cardiovascular outcomes and all-cause mortality were investigated. Overall lifestyle was subsequently categorized into ideal (having at least 3 ideal life style factors), poor (having at least 3 poor lifestyle factors), or intermediate (all other combinations) ^[1]^. Lifestyle factors included smoking (Ideal: never, previous; Poor: current), body mass index (BMI) (Ideal: normal weight, 18.5 ≤ BMI < 25 kg/m^2^, overweight, 25 ≤ BMI < 30 kg/m^2^; Poor: obesity, BMI ≥ 30 kg/m^2^), physical activity (Ideal: moderate, high; Poor: low), and diet (Ideal: healthy; Poor: unhealthy) ^[1]^. The self-reported physical activity level was assessed using the well-validated International Physical Activity Questionnaire-Short Form ^[2]^. Healthy diet was based on eating at least 5 portions of a variety of fruits and vegetables every day, following the NHS guidelines ^[3]^.

1. Said MA, Verweij N, van der Harst P. Associations of Combined Genetic and Lifestyle Risks with Incident Cardiovascular Disease and Diabetes in the UK Biobank Study. JAMA Cardiology. 2018 Aug;3(8):693-702.

2. Cleland, C., et al., Validity of the International Physical Activity Questionnaire (IPAQ) for assessing moderate-to-vigorous physical activity and sedentary behaviour of older adults in the United Kingdom. BMC Med Res Methodol, 2018. 18(1): p. 176.

3. Peng, H., et al., Lifestyle Factors, Genetic Risk, and Cardiovascular Disease Risk among Breast Cancer Survivors: A Prospective Cohort Study in UK Biobank. Nutrients, 2023. 15(4).

**Table S1 31 Single nucleotide polymorphisms associated with myocardial infarction**

|  | **SNP** | **CHR** | **A1** | **A2** | **OR** | **P** | **SE** | **BP** |
| --- | --- | --- | --- | --- | --- | --- | --- | --- |
| 1 | rs11591147 | 1 | G | T | 1.31 | 4.3E-11 | 0.0177863 | 55505647 |
| 2 | rs12740374 | 1 | G | T | 1.11 | 1.6E-17 | 0.0053198 | 109817590 |
| 3 | rs4846384 | 1 | G | C | 1.09 | 3.9E-15 | 0.0047628 | 222797614 |
| 4 | rs2886722 | 2 | G | A | 1.07 | 1.6E-09 | 0.0048697 | 85742297 |
| 5 | rs150289443 | 2 | TTA | T | 1.11 | 1.3E-10 | 0.0070518 | 203829582 |
| 6 | rs112995129 | 3 | AAAAAC | A | 1.08 | 2.5E-10 | 0.0052827 | 136134167 |
| 7 | rs9349379 | 6 | G | A | 1.12 | 7.8E-28 | 0.0045008 | 12903957 |
| 8 | rs1966248 | 6 | A | T | 1.09 | 2.8E-14 | 0.0049198 | 134159622 |
| 9 | rs10455872 | 6 | G | A | 1.37 | 3.4E-58 | 0.0085014 | 161010118 |
| 10 | rs3918226 | 7 | T | C | 1.12 | 1.2E-08 | 0.0086352 | 150690176 |
| 11 | rs7011846 | 8 | A | G | 1.21 | 1.7E-09 | 0.0137422 | 19785656 |
| 12 | rs2954021 | 8 | A | G | 1.06 | 1.2E-08 | 0.0044398 | 126482077 |
| 13 | rs2891168 | 9 | G | A | 1.2 | 8.1E-69 | 0.0045163 | 22098619 |
| 14 | rs376993806 | 9 | G | A | 1.07 | 5.1E-08 | 0.0053937 | 139246588 |
| 15 | rs1704221 | 10 | G | A | 1.11 | 1.9E-10 | 0.0071159 | 44710930 |
| 16 | rs1412444 | 10 | T | C | 1.07 | 3.6E-09 | 0.004979 | 91002927 |
| 17 | rs1964600 | 11 | A | G | 1.07 | 1.9E-08 | 0.0052276 | 1680664 |
| 18 | rs1384705 | 11 | C | T | 1.08 | 2.7E-11 | 0.005017 | 103696851 |
| 19 | rs10841443 | 12 | G | C | 1.06 | 2.4E-08 | 0.0045348 | 20220033 |
| 20 | rs7137258 | 12 | A | C | 1.13 | 3.6E-08 | 0.0096341 | 54512164 |
| 21 | rs35350651 | 12 | A | AC | 1.08 | 1.6E-14 | 0.0043525 | 111907431 |
| 22 | rs7485656 | 12 | G | A | 1.09 | 2.2E-08 | 0.0066887 | 125315647 |
| 23 | rs11617955 | 13 | T | A | 1.1 | 3.9E-09 | 0.0070296 | 110818102 |
| 24 | rs11632963 | 15 | A | G | 1.06 | 3.6E-09 | 0.004288 | 79132644 |
| 25 | rs764429222 | 15 | C | CT | 1.09 | 6.5E-15 | 0.0048021 | 91428521 |
| 26 | rs77870048 | 16 | C | T | 1.14 | 2.4E-08 | 0.0101973 | 69965021 |
| 27 | rs34338189 | 17 | T | TC | 1.06 | 4E-08 | 0.0046087 | 47340660 |
| 28 | rs368803408 | 19 | AT | A | 1.11 | 4.2E-11 | 0.0068704 | 11196651 |
| 29 | rs12983897 | 19 | G | C | 1.06 | 1.5E-08 | 0.0044698 | 17858564 |
| 30 | rs1065853 | 19 | G | T | 1.17 | 1.7E-16 | 0.0082734 | 45413233 |
| 31 | rs28451064 | 21 | A | G | 1.12 | 1.9E-12 | 0.0069896 | 35593827 |

Note, SNP, Single nucleotide polymorphisms; CHR, chromosome; A1, effect allele; A2, other allele; OR, odds ratio; SE, standard error;

**Table S2 32 Single nucleotide polymorphisms associated with stroke**

|  | **SNP** | **CHR** | **A1** | **A2** | **OR** | **P** | **SE** | **BP** |
| --- | --- | --- | --- | --- | --- | --- | --- | --- |
| 1 | rs1052053 | 1 | G | A | 1.06 | 2.7E-14 | 0.003324 | 156202173 |
| 2 | rs12037987 | 1 | C | T | 1.07 | 2.73E-08 | 0.005287 | 113042822 |
| 3 | rs12124533 | 1 | T | C | 1.17 | 1.22E-08 | 0.011969 | 115657799 |
| 4 | rs146390073 | 1 | T | C | 1.95 | 2.2E-08 | 0.051834 | 241306248 |
| 5 | rs880315 | 1 | C | T | 1.05 | 3.62E-10 | 0.00338 | 10796866 |
| 6 | rs12476527 | 2 | G | T | 1.05 | 6.44E-08 | 0.003919 | 26915624 |
| 7 | rs7610618 | 3 | T | C | 2.33 | 1.44E-08 | 0.064806 | 149157706 |
| 8 | rs13143308 | 4 | T | G | 1.32 | 1.86E-47 | 0.008332 | 111714419 |
| 9 | rs17612742 | 4 | C | T | 1.19 | 1.46E-11 | 0.011189 | 148414651 |
| 10 | rs34311906 | 4 | C | T | 1.07 | 1.07E-08 | 0.005138 | 113732090 |
| 11 | rs6825454 | 4 | C | T | 1.06 | 7.43E-10 | 0.00411 | 155501188 |
| 12 | rs11957829 | 5 | A | G | 1.07 | 7.51E-09 | 0.005084 | 121515195 |
| 13 | rs6891174 | 5 | A | G | 1.11 | 5.82E-09 | 0.007785 | 172640590 |
| 14 | rs16896398 | 6 | T | A | 1.05 | 1.3E-08 | 0.003727 | 43262704 |
| 15 | rs4959130 | 6 | A | G | 1.08 | 1.42E-09 | 0.005522 | 1356916 |
| 16 | rs2107595 | 7 | A | G | 1.21 | 3.65E-15 | 0.010524 | 19049388 |
| 17 | rs42039 | 7 | C | T | 1.07 | 6.55E-09 | 0.005064 | 92244422 |
| 18 | rs10820405 | 9 | G | A | 1.2 | 4.51E-08 | 0.014477 | 106010237 |
| 19 | rs635634 | 9 | T | C | 1.08 | 9.18E-09 | 0.005818 | 136155000 |
| 20 | rs7859727 | 9 | T | C | 1.05 | 4.22E-10 | 0.003393 | 22102165 |
| 21 | rs2295786 | 10 | A | T | 1.05 | 1.8E-10 | 0.003323 | 105616482 |
| 22 | rs2005108 | 11 | T | C | 1.08 | 3.33E-08 | 0.006052 | 102770353 |
| 23 | rs3184504 | 12 | T | C | 1.08 | 2.17E-14 | 0.004375 | 111884608 |
| 24 | rs35436 | 12 | C | T | 1.05 | 2.87E-08 | 0.003818 | 115554523 |
| 25 | rs7304841 | 12 | A | C | 1.05 | 4.93E-08 | 0.003885 | 20577593 |
| 26 | rs9526212 | 13 | G | A | 1.06 | 5.03E-10 | 0.00407 | 47225745 |
| 27 | rs4932370 | 15 | A | G | 1.05 | 2.88E-08 | 0.003819 | 91404705 |
| 28 | rs12445022 | 16 | A | G | 1.06 | 1.05E-10 | 0.003918 | 87575332 |
| 29 | rs12932445 | 16 | C | T | 1.2 | 6.86E-18 | 0.009189 | 73069888 |
| 30 | rs11867415 | 17 | G | A | 1.09 | 4.81E-08 | 0.006857 | 1571818 |
| 31 | rs2229383 | 19 | T | G | 1.05 | 4.72E-08 | 0.00388 | 10794630 |
| 32 | rs8103309 | 19 | T | C | 1.05 | 3.4E-08 | 0.003839 | 11174935 |

Note, SNP, Single nucleotide polymorphisms; CHR, chromosome; A1, effect allele; A2, other allele; OR, odds ratio; SE, standard error;

**Table S3 12 Single nucleotide polymorphisms associated with heart failure**

|  | SNP | CHR | A1 | A2 | OR | P | SE | BP |
| --- | --- | --- | --- | --- | --- | --- | --- | --- |
| 1 | rs660240 | 1 | C | T | 1.06 | 3.25E-10 | 0.004025521 | 109817838 |
| 2 | rs17042102 | 4 | A | G | 1.12 | 5.71E-20 | 0.005379204 | 111668626 |
| 3 | rs11745324 | 5 | G | A | 1.05 | 2.35E-08 | 0.003794621 | 137012171 |
| 4 | rs4135240 | 6 | T | C | 1.05 | 6.84E-09 | 0.003656602 | 36647680 |
| 5 | rs55730499 | 6 | T | C | 1.11 | 1.83E-11 | 0.006745514 | 161005610 |
| 6 | rs140570886 | 6 | C | T | 1.24 | 7.69E-11 | 0.014358114 | 161013013 |
| 7 | rs1556516 | 9 | C | G | 1.06 | 1.57E-15 | 0.003174618 | 22100176 |
| 8 | rs600038 | 9 | C | T | 1.06 | 3.68E-09 | 0.004290606 | 136151806 |
| 9 | rs4746140 | 10 | G | C | 1.07 | 1.1E-09 | 0.004821613 | 75417249 |
| 10 | rs17617337 | 10 | C | T | 1.06 | 3.65E-09 | 0.004289623 | 121426884 |
| 11 | rs4766578 | 12 | T | A | 1.04 | 4.9E-08 | 0.003122575 | 111904371 |
| 12 | rs56094641 | 16 | G | A | 1.05 | 1.21E-08 | 0.003718523 | 53806453 |

Note, SNP, Single nucleotide polymorphisms; CHR, chromosome; A1, effect allele; A2, other allele; OR, odds ratio; SE, standard error;

**Table S4 Associations of metabolic status, BMI status, and BMI-metabolic status with the risk of specific cardiovascular outcomes stratified by the levels of PRS**

| **Outcomes** | **Exposures** | **Case (%)** | **High PRS** | **Moderate PRS** | **Low PRS** | ***P* for interaction ^a^** |
| --- | --- | --- | --- | --- | --- | --- |
| **Coronary disease** | **Metabolic status** |  |  |  |  |  |
|  | MU | 16 307 (14.4) | 1.0 (Reference) | 1.0 (Reference) | 1.0 (Reference) | 0.003 |
|  | MH | 18 417 (6.3) | 0.63 (0.60, 0.65) * | 0.59 (0.57, 0.61) * | 0.60 (0.57, 0.64) * |  |
|  | *P* for trend |  | < 0.001 | < 0.001 | < 0.001 |  |
|  | **BMI status** |  |  |  |  |  |
|  | Obesity | 13204 (12.0) | 1.0 (Reference) | 1.0 (Reference) | 1.0 (Reference) | < 0.001 |
|  | Overweight | 17711 (9.0) | 0.79 (0.75, 0.82) * | 0.73 (0.70, 0.75) * | 0.68 (0.64, 0.72) * |  |
|  | Normal weight | 8543 (5.5) | 0.60 (0.57, 0.63) * | 0.55 (0.53, 0.57) * | 0.53 (0.50, 0.58) * |  |
|  | Per 1‑point increase |  | 0.78 (0.76, 0.80) * | 0.74 (0.73, 0.76) * | 0.73 (0.70, 0.75) * | < 0.001 |
|  | *P* for trend |  | < 0.001 | < 0.001 | < 0.001 |  |
|  | **BMI-metabolic status** |  |  |  |  |  |
|  | MUO | 8463 (14.4) | 1.0 (Reference) | 1.0 (Reference) | 1.0 (Reference) | 0.006 |
|  | MUOW | 6434 (14.4) | 0.91 (0.86, 0.97) * | 0.84 (0.81, 0.88) * | 0.82 (0.75, 0.90) * |  |
|  | MUN | 1347 (14.5) | 0.84 (0.76, 0.94) * | 0.79 (0.73, 0.85) * | 0.77 (0.65, 0.91) * |  |
|  | MHO | 3122 (8.4) | 0.75 (0.69, 0.81) * | 0.72 (0.68, 0.76) * | 0.76 (0.68, 0.84) * |  |
|  | MHOW | 9073 (7.1) | 0.63 (0.59, 0.66) * | 0.56 (0.54, 0.59) * | 0.55 (0.51, 0.60) * |  |
|  | MHN | 6058 (4.9) | 0.50 (0.47, 0.53) * | 0.45 (0.43, 0.47) * | 0.46 (0.42, 0.50) * |  |
|  | Per 1‑point increase |  | 0.877 (0.867, 0.886) | 0.860 (0.854, 0.866) * | 0.862 (0.849, 0.876) * | < 0.001 |
|  | *P* for trend |  | < 0.001 | < 0.001 | < 0.001 |  |
| **Coronary disease mortality** | **Metabolic status** |  |  |  |  |  |
|  | MU | 2341 (2.1) | 1.0 (Reference) | 1.0 (Reference) | 1.0 (Reference) | 0.826 |
|  | MH | 1992 (0.7) | 0.57 (0.51, 0.64) * | 0.58 (0.53, 0.62) * | 0.56 (0.47, 0.67) * |  |
|  | *P* for trend |  | < 0.001 | < 0.001 | < 0.001 |  |
|  | **BMI status** |  |  |  |  |  |
|  | Obesity | 1781 (1.6) | 1.0 (Reference) | 1.0 (Reference) | 1.0 (Reference) | 0.556 |
|  | Overweight | 2087 (1.1) | 0.69 (0.61, 0.77) * | 0.67 (0.62, 0.73) | 0.59 (0.49, 0.71) |  |
|  | Normal weight | 1024 (0.7) | 0.58 (0.50, 0.67) * | 0.58 (0.52, 0.64) | 0.59 (0.47, 0.73) |  |
|  | Per 1‑point increase |  | 0.75 (0.70, 0.81) * | 0.75 (0.71, 0.79) | 0.74 (0.66, 0.83) | 0.984 |
|  | *P* for trend |  | < 0.001 | < 0.001 | < 0.001 |  |
|  | **BMI-metabolic status** |  |  |  |  |  |
|  | MUO | 1239 (2.1) | 1.0 (Reference) | 1.0 (Reference) | 1.0 (Reference) | 0.791 |
|  | MUOW | 878 (2) | 0.80 (0.69, 0.94) * | 0.77 (0.69, 0.87) * | 0.65 (0.50, 0.85) * |  |
|  | MUN | 208 (2.2) | 0.81 (0.62, 1.06) | 0.76 (0.63, 0.93) * | 0.90 (0.60, 1.36) |  |
|  | MHO | 321 (0.9) | 0.60 (0.47, 0.76) * | 0.64 (0.54, 0.75) * | 0.65 (0.47, 0.91) * |  |
|  | MHOW | 964 (0.8) | 0.52 (0.45, 0.61) * | 0.50 (0.45, 0.56) * | 0.43 (0.33, 0.55) * |  |
|  | MHN | 667 (0.5) | 0.44 (0.37, 0.53) * | 0.46 (0.40, 0.52) * | 0.44 (0.34, 0.57) * |  |
|  | Per 1‑point increase |  | 0.85 (0.83, 0.88) * | 0.86 (0.84, 0.87) * | 0.85 (0.81, 0.89) * | 0.785 |
|  | *P* for trend |  | < 0.001 | < 0.001 | < 0.001 |  |
| **Myocardial infarction** | **Metabolic status** |  |  |  |  |  |
|  | MU | 9166 (7.6) | 1.0 (Reference) | 1.0 (Reference) | 1.0 (Reference) | 0.639 |
|  | MH | 7837 (2.7) | 0.53 (0.50, 0.56) * | 0.52 (0.50, 0.54) * | 0.54 (0.50, 0.58) * |  |
|  | *P* for trend |  | < 0.001 | < 0.001 | < 0.001 |  |
|  | **BMI status** |  |  |  |  |  |
|  | Obesity | 6693 (5.8) | 1.0 (Reference) | 1.0 (Reference) | 1.0 (Reference) | 0.151 |
|  | Overweight | 8728 (4.3) | 0.78 (0.74, 0.83) * | 0.74 (0.70, 0.77) * | 0.69 (0.64, 0.75) * |  |
|  | Normal weight | 3895 (2.5) | 0.58 (0.54, 0.63) * | 0.56 (0.53, 0.59) * | 0.54 (0.49, 0.60) * |  |
|  | Per 1‑point increase |  | 0.766 (0.738, 0.796) * | 0.766 (0.725, 0.764) * | 0.73 (0.69, 0.77) * | 0.184 |
|  | *P* for trend |  | < 0.001 | < 0.001 | < 0.001 |  |
|  | **BMI-metabolic status** |  |  |  |  |  |
|  | MUO | 4603 (7.3) | 1.0 (Reference) | 1.0 (Reference) | 1.0 (Reference) | 0.290 |
|  | MUOW | 3734 (7.8) | 0.96 (0.88, 1.04) * | 0.90 (0.85, 0.95) * | 0.83 (0.74, 0.94) * |  |
|  | MUN | 785 (7.7) | 0.87 (0.75, 1.00) | 0.80 (0.72, 0.88) * | 0.88 (0.72, 1.08) |  |
|  | MHO | 1243 (3.3) | 0.60 (0.53, 0.69) * | 0.58 (0.53, 0.63) * | 0.69 (0.59, 0.80) * |  |
|  | MHOW | 3922 (3.0) | 0.54 (0.50, 0.59) * | 0.51 (0.48, 0.54) * | 0.49 (0.44, 0.55) * |  |
|  | MHN | 2596 (2.1) | 0.44 (0.40, 0.48) * | 0.43 (0.40, 0.46) * | 0.42 (0.37, 0.48) * |  |
|  | Per 1‑point increase |  | 0.845 (0.832, 0.859) * | 0.841 (0.832, 0.850) * | 0.842 (0.824, 0.860) * | 0.603 |
|  | *P* for trend |  | < 0.001 | < 0.001 | < 0.001 |  |
| **Myocardial infarction mortality** | **Metabolic status** |  |  |  |  |  |
|  | MU | 952 (0.8) | 1.0 (Reference) | 1.0 (Reference) | 1.0 (Reference) | 0.440 |
|  | MH | 761 (0.3) | 0.63 (0.51, 0.77) * | 0.55 (0.48, 0.62) * | 0.55 (0.41, 0.70) * |  |
|  | *P* for trend |  | < 0.001 | < 0.001 | < 0.001 |  |
|  | **BMI status** |  |  |  |  |  |
|  | Obesity | 710 (0.6) | 1.0 (Reference) | 1.0 (Reference) | 1.0 (Reference) | 0.290 |
|  | Overweight | 812 (0.4) | 0.57 (0.46, 0.70) * | 0.72 (0.63, 0.82) * | 0.60 (0.45, 0.78) * |  |
|  | Normal weight | 394 (0.3) | 0.51 (0.40, 0.66) * | 0.60 (0.51, 0.71) * | 0.58 (0.42, 0.80) * |  |
|  | Per 1‑point increase |  | 0.69 (0.61, 0.79) * | 0.77 (0.71, 0.83) * | 0.74 (0.62, 0.87) * | 0.353 |
|  | *P* for trend |  | < 0.001 | < 0.001 | < 0.001 |  |
|  | **BMI-metabolic status** |  |  |  |  |  |
|  | MUO | 497 (0.8) | 1.0 (Reference) | 1.0 (Reference) | 1.0 (Reference) | 0.366 |
|  | MUOW | 365 (0.8) | 0.64 (0.48, 0.85) * | 0.90 (0.76, 1.06) | 0.62 (0.42, 0.90) * |  |
|  | MUN | 83 (0.8) | 0.82 (0.52, 1.29) | 0.77 (0.57, 1.04) | 0.71 (0.39, 1.32) |  |
|  | MHO | 129 (0.3) | 0.83 (0.57, 1.19) | 0.64 (0.49, 0.82) * | 0.47 (0.27, 0.82) * |  |
|  | MHOW | 361 (0.3) | 0.50 (0.38, 0.66) * | 0.50 (0.42, 0.59) * | 0.44 (0.31, 0.63) * |  |
|  | MHN | 258 (0.2) | 0.43 (0.31, 0.59) * | 0.49 (0.40, 0.59) * | 0.38 (0.25, 0.58) * |  |
|  | Per 1‑point increase |  | 0.86 (0.82, 0.91) * | 0.85 (0.82, 0.88) * | 0.83 (0.78, 0.89) * | 0.858 |
|  | *P* for trend |  | < 0.001 | < 0.001 | < 0.001 |  |
| **Stroke** | **metabolic status** |  |  |  |  |  |
|  | MU | 4820 (3.9) | 1.0 (Reference) | 1.0 (Reference) | 1.0 (Reference) | 0.309 |
|  | MH | 6053 (2.0) | 0.76 (0.70, 0.83) * | 0.72 (0.69, 0.76) * | 0.78 (0.71, 0.85) * |  |
|  | *P* for trend |  | < 0.001 | < 0.001 | < 0.001 |  |
|  | **BMI status** |  |  |  |  |  |
|  | Obesity | 3640 (3.1) | 1.0 (Reference) | 1.0 (Reference) | 1.0 (Reference) | 0.031 |
|  | Overweight | 5420 (2.6) | 0.86 (0.78, 0.94) * | 0.81 (0.77, 0.86) * | 0.97 (0.88, 1.07) |  |
|  | Normal weight | 3252 (2.1) | 0.83 (0.75, 0.92) * | 0.81 (0.76, 0.86) * | 0.84 (0.75, 0.94) * |  |
|  | Per 1‑point increase |  | 0.91 (0.86, 0.96) * | 0.90 (0.87, 0.93) * | 0.92 (0.87, 0.97) * | 0.545 |
|  | *P* for trend |  | < 0.001 | < 0.001 | < 0.001 |  |
|  | **BMI-metabolic status** |  |  |  |  |  |
|  | MUO | 2369 (3.7) | 1.0 (Reference) | 1.0 (Reference) | 1.0 (Reference) | 0.395 |
|  | MUOW | 1954 (4) | 0.92 (0.81, 1.05) | 0.88 (0.81, 0.95) * | 1.04 (0.90, 1.19) |  |
|  | MUN | 465 (4.4) | 0.99 (0.80, 1.21) | 0.94 (0.83, 1.07) | 1.03 (0.80, 1.31) |  |
|  | MHO | 828 (2.2) | 0.79 (0.67, 0.94) * | 0.72 (0.65, 0.80) * | 0.77 (0.64, 0.93) * |  |
|  | MHOW | 2788 (2.2) | 0.71 (0.63, 0.81) * | 0.67 (0.62, 0.72) * | 0.83 (0.73, 0.95) * |  |
|  | MHN | 2365 (1.9) | 0.73 (0.64, 0.83) * | 0.69 (0.64, 0.74) * | 0.76 (0.66, 0.87) * |  |
|  | Per 1‑point increase |  | 0.93 (0.91, 0.95) * | 0.92 (0.91, 0.93) * | 0.94 (0.92, 0.96) * | 0.224 |
|  | *P* for trend |  | < 0.001 | < 0.001 | < 0.001 |  |
| **Stroke mortality** | **metabolic status** |  |  |  |  |  |
|  | MU | 1077 (0.9) | 1.0 (Reference) | 1.0 (Reference) | 1.0 (Reference) | 0.379 |
|  | MH | 1192 (0.4) | 0.68 (0.56, 0.82) * | 0.68 (0.62, 0.76) * | 0.79 (0.64, 0.96) * |  |
|  | *P* for trend |  | < 0.001 | < 0.001 | < 0.001 |  |
|  | **BMI status** |  |  |  |  |  |
|  | Obesity | 744 (0.6) | 1.0 (Reference) | 1.0 (Reference) | 1.0 (Reference) | 0.051 |
|  | Overweight | 1077 (0.5) | 0.93 (0.76, 1.14) | 0.76 (0.67, 0.86) * | 1.07 (0.86, 1.35) |  |
|  | Normal weight | 748 (0.5) | 0.90 (0.72, 1.13) | 0.95 (0.83, 1.09) | 1.07 (0.83, 1.38) |  |
|  | Per 1‑point increase |  | 0.95 (0.85, 1.06) | 0.97 (0.91, 1.04) | 1.03 (0.91, 1.17) | 0.596 |
|  | *P* for trend |  | 0.362 | 0.469 | 0.600 |  |
|  | **BMI-metabolic status** |  |  |  |  |  |
|  | MUO | 497 (0.8) | 1.0 (Reference) | 1.0 (Reference) | 1.0 (Reference) | 0.439 |
|  | MUOW | 433 (0.9) | 0.96 (0.72, 1.27) | 0.90 (0.76, 1.07) | 1.18 (0.86, 1.62) |  |
|  | MUN | 141 (1.3) | 1.28 (0.86, 1.91) | 1.38 (1.09, 1.75) | 1.40 (0.85, 2.29) |  |
|  | MHO | 158 (0.4) | 0.58 (0.38, 0.87) * | 0.72 (0.57, 0.90) * | 0.87 (0.57, 1.33) |  |
|  | MHOW | 504 (0.4) | 0.73 (0.56, 0.95) * | 0.58 (0.50, 0.69) * | 0.80 (0.59, 1.08) |  |
|  | MHN | 503 (0.4) | 0.67 (0.50, 0.89) * | 0.76 (0.64, 0.89) * | 0.93 (0.68, 1.27) |  |
|  | Per 1‑point increase |  | 0.917 (0.873, 0.962) * | 0.918 (0.892, 0.945) * | 0.953 (0.903, 1.005) | 0.410 |
|  | *P* for trend |  | < 0.001 | < 0.001 | 0.004 |  |
| **Heart failure** | **metabolic status** |  |  |  |  |  |
|  | MU | 8273 (6.7) | 1.0 (Reference) | 1.0 (Reference) | 1.0 (Reference) | 0.272 |
|  | MH | 6684 (2.3) | 0.50 (0.47, 0.54) * | 0.53 (0.51, 0.56) * | 0.52 (0.48, 0.56) * |  |
|  | *P* for trend |  | < 0.001 | < 0.001 | < 0.001 |  |
|  | **BMI status** |  |  |  |  |  |
|  | Obesity | 7132 (6.1) | 1.0 (Reference) | 1.0 (Reference) | 1.0 (Reference) | 0.782 |
|  | Overweight | 6637 (3.2) | 0.54 (0.50, 0.58) * | 0.53 (0.51, 0.55) * | 0.52 (0.48, 0.57) * |  |
|  | Normal weight | 3218 (2.0) | 0.42 (0.39, 0.46) * | 0.44 (0.42, 0.46) * | 0.43 (0.39, 0.47) * |  |
|  | Per 1‑point increase |  | 0.63 (0.60, 0.66) * | 0.64 (0.62, 0.66) * | 0.63 (0.60, 0.66) * | 0.858 |
|  | *P* for trend |  | < 0.001 | < 0.001 | < 0.001 |  |
|  | **BMI-metabolic status** |  |  |  |  |  |
|  | MUO | 4770 (7.5) | 1.0 (Reference) | 1.0 (Reference) | 1.0 (Reference) | 0.427 |
|  | MUOW | 2841 (5.7) | 0.68 (0.62, 0.75) * | 0.62 (0.58, 0.66) * | 0.63 (0.57, 0.71) * |  |
|  | MUN | 607 (5.7) | 0.55 (0.46, 0.66) * | 0.61 (0.54, 0.67) * | 0.57 (0.47, 0.70) * |  |
|  | MHO | 1458 (3.8) | 0.68 (0.60, 0.77) * | 0.70 (0.64, 0.75) * | 0.72 (0.63, 0.83) * |  |
|  | MHOW | 3012 (2.3) | 0.39 (0.35, 0.43) * | 0.41 (0.39, 0.44) * | 0.38 (0.34, 0.42) * |  |
|  | MHN | 2130 (1.7) | 0.35 (0.31, 0.39) * | 0.34 (0.32, 0.37) * | 0.34 (0.30, 0.39) * |  |
|  | Per 1‑point increase |  | 0.813 (0.793, 0.828) * | 0.822 (0.813, 0.821) * | 0.815 (0.798, 0.831) * | 0.477 |
|  | *P* for trend |  | < 0.001 | < 0.001 | < 0.001 |  |
| **Heart failure mortality** | **Metabolic status** |  |  |  |  |  |
|  | MU | 1316 (1.1) | 1.0 (Reference) | 1.0 (Reference) | 1.0 (Reference) | 0.768 |
|  | MH | 822 (0.3) | 0.44 (0.36, 0.53) * | 0.47 (0.42, 0.53) * | 0.45 (0.36, 0.56) * |  |
|  | *P* for trend |  | < 0.001 | < 0.001 | < 0.001 |  |
|  | **BMI status** |  |  |  |  |  |
|  | Obesity | 1091 (0.9) | 1.0 (Reference) | 1.0 (Reference) | 1.0 (Reference) | 0.407 |
|  | Overweight | 861 (0.4) | 0.50 (0.42, 0.61) * | 0.48 (0.43, 0.54) * | 0.39 (0.31, 0.49) * |  |
|  | Normal weight | 462 (0.3) | 0.50 (0.40, 0.63) * | 0.43 (0.37, 0.50) * | 0.44 (0.35, 0.57) * |  |
|  | Per 1‑point increase |  | 0.67 (0.59, 0.75) * | 0.62 (0.57, 0.66) * | 0.61 (0.53, 0.70) * | 0.551 |
|  | *P* for trend |  | < 0.001 | < 0.001 | < 0.001 |  |
|  | **BMI-metabolic status** |  |  |  |  |  |
|  | MUO | 773 (1.2) | 1.0 (Reference) | 1.0 (Reference) | 1.0 (Reference) | 0.655 |
|  | MUOW | 417 (0.8) | 0.64 (0.50, 0.81) * | 0.56 (0.48, 0.66) * | 0.52 (0.39, 0.70) * |  |
|  | MUN | 110 (1.0) | 0.72 (0.48, 1.08) | 0.59 (0.45, 0.77) * | 0.74 (0.47, 1.15) |  |
|  | MHO | 179 (0.5) | 0.52 (0.36, 0.76) * | 0.59 (0.48, 0.73) * | 0.68 (0.48, 0.98) * |  |
|  | MHOW | 335 (0.3) | 0.32 (0.24, 0.42) * | 0.33 (0.28, 0.38) * | 0.26 (0.19, 0.35) * |  |
|  | MHN | 282 (0.2) | 0.36 (0.27, 0.48) * | 0.31 (0.26, 0.38) * | 0.34 (0.25, 0.47) * |  |
|  | Per 1‑point increase |  | 0.80 (0.76, 0.84) * | 0.793 (0.768, 0.818) * | 0.792 (0.750, 0.838) * | 0.872 |
|  | *P* for trend |  | < 0.001 | < 0.001 | < 0.001 |  |
| **Atrial fibrillation** | **metabolic status** |  |  |  |  |  |
|  | MU | 13 359 (10.9) | 1.0 (Reference) | 1.0 (Reference) | 1.0 (Reference) | < 0.001 |
|  | MH | 16 128 (5.5) | 0.75 (0.72, 0.78) * | 0.72 (0.69, 0.74) * | 0.68 (0.63, 0.73) * |  |
|  | *P* for trend |  | < 0.001 | < 0.001 | < 0.001 |  |
|  | **BMI status** |  |  |  |  |  |
|  | Obesity | 11941 (10.3) | 1.0 (Reference) | 1.0 (Reference) | 1.0 (Reference) | 0.002 |
|  | Overweight | 14007 (6.9) | 0.66 (0.63, 0.69) * | 0.61 (0.59, 0.63) * | 0.62 (0.57, 0.67) * |  |
|  | Normal weight | 7565 (4.8) | 0.56 (0.54, 0.59) * | 0.55 (0.53, 0.57) * | 0.50 (0.46, 0.55) * |  |
|  | Per 1‑point increase |  | 0.74 (0.72, 0.76) * | 0.73 (0.71, 0.74) * | 0.70 (0.67, 0.73) * | 0.011 |
|  | *P* for trend |  | < 0.001 | < 0.001 | < 0.001 |  |
|  | **BMI-metabolic status** |  |  |  |  |  |
|  | MUO | 7413 (11.8) | 1.0 (Reference) | 1.0 (Reference) | 1.0 (Reference) | < 0.001 |
|  | MUOW | 4925 (10.1) | 0.73 (0.69, 0.78) * | 0.66 (0.63, 0.70) * | 0.68 (0.61, 0.75) * |  |
|  | MUN | 962 (9.2) | 0.56 (0.49, 0.63) * | 0.61 (0.56, 0.67) * | 0.45 (0.36, 0.57) * |  |
|  | MHO | 3031 (8.0) | 0.97 (0.90, 1.05) | 0.89 (0.84, 0.94) * | 0.82 (0.72, 0.94) * |  |
|  | MHOW | 7381 (5.7) | 0.61 (0.57, 0.64) * | 0.56 (0.53, 0.58) * | 0.53 (0.48, 0.59) * |  |
|  | MHN | 5580 (4.5) | 0.54 (0.51, 0.58) * | 0.51 (0.49, 0.54) * | 0.47 (0.42, 0.53) * |  |
|  | Per 1‑point increase |  | 0.900 (0.891, 0.910) * | 0.891 (0.884, 0.898) * | 0.88 (0.86, 0.89) * | < 0.001 |
|  | *P* for trend |  | < 0.001 | < 0.001 | < 0.001 |  |
| **Atrial fibrillation mortality** | **Metabolic status** |  |  |  |  |  |
|  | MU | 677 (0.6) | 1.0 (Reference) | 1.0 (Reference) | 1.0 (Reference) | 0.888 |
|  | MH | 562 (0.2) | 0.58 (0.48, 0.72) * | 0.60 (0.51, 0.70) * | 0.56 (0.39, 0.82) * |  |
|  | *P* for trend |  | < 0.001 | < 0.001 | < 0.001 |  |
|  | **BMI status** |  |  |  |  |  |
|  | Obesity | 592 (0.5) | 1.0 (Reference) | 1.0 (Reference) | 1.0 (Reference) | < 0.001 |
|  | Overweight | 532 (0.3) | 0.52 (0.42, 0.64) * | 0.49 (0.41, 0.57) * | 0.56 (0.39, 0.80) * |  |
|  | Normal weight | 293 (0.2) | 0.55 (0.43, 0.70) * | 0.52 (0.43, 0.63) * | 0.18 (0.10, 0.34) * |  |
|  | Per 1‑point increase |  | 0.70 (0.62, 0.80) * | 0.68 (0.62, 0.76) * | 0.47 (0.37, 0.60) * | 0.017 |
|  | *P* for trend |  | < 0.001 | < 0.001 | < 0.001 |  |
|  | **BMI-metabolic status** |  |  |  |  |  |
|  | MUO | 386 (0.6) | 1.0 (Reference) | 1.0 (Reference) | 1.0 (Reference) | 0.112 |
|  | MUOW | 232 (0.5) | 0.69 (0.52, 0.91) * | 0.56 (0.44, 0.69) * | 0.63 (0.38, 1.04) |  |
|  | MUN | 56 (0.5) | 0.63 (0.38, 1.06) | 0.69 (0.48, 0.98) * | 0.31 (0.09, 1.01) |  |
|  | MHO | 130 (0.3) | 0.86 (0.61, 1.21) | 0.78 (0.59, 1.03) | 1.16 (0.65, 2.07) |  |
|  | MHOW | 225 (0.2) | 0.38 (0.29, 0.51) * | 0.38 (0.30, 0.47) * | 0.43 (0.26, 0.71) * |  |
|  | MHN | 197 (0.2) | 0.48 (0.35, 0.64) * | 0.44 (0.35, 0.56) * | 0.18 (0.09, 0.38) * |  |
|  | Per 1‑point increase |  | 0.849 (0.805, 0.894) * | 0.848 (0.814, 0.883) * | 0.79 (0.72, 0.88) * | 0.395 |
|  | *P* for trend |  | < 0.001 | < 0.001 | < 0.001 |  |

Note, BMI, body mass index; MH status was defined as < 3 abnormal components; MH, metabolically healthy; MU, metabolically unhealthy; MHN, metabolically healthy normal weight; MHOW, metabolically healthy overweight; MHO, metabolically healthy obesity; MUN, metabolically unhealthy normal weight; MUOW, metabolically unhealthy overweight; MUO, metabolically unhealthy obesity; PRS, polygenic risk score; the PRSs presented are specifically used based on corresponding outcomes; each model was adjusted for age, sex, race, Townsend Deprivation Index, annual household income, education attainment, 22 assessment centers, the first 5 principal components of ancestry, family history of diabetes, family history of high blood pressure, and lifestyle factors including sleep duration, healthy diet, physical activity, smoking status, and alcohol intake frequency; **^a^**, likelihood tests was applied to test the significance of interaction term by comparing the model with and without the interaction term; *, *P* < 0.05

**Table S5 Combined effects of metabolic status (MH status:** **< 3 abnormal components) and PRSs on the risk of** **the specific cardiovascular outcomes**

| **Outcomes** | **Case (%)** | **Model 1** | | | | **Model 2** | | | |
| --- | --- | --- | --- | --- | --- | --- | --- | --- | --- |
|  |  | **High PRS** | **Moderate PRS** | **Low PRS** | ***P* for interaction ^a^** | **High PRS** | **Moderate PRS** | **Low PRS** | ***P* for interaction ^a^** |
| **Coronary disease** | |  |  |  | 0.002 |  |  |  | 0.003 |
| MU | 16 307 (14.4) | 1.0 (Reference) | 0.68 (0.66, 0.70) * | 0.45 (0.42, 0.47) * |  | 1.0 (Reference) | 0.68 (0.65, 0.70) * | 0.45 (0.42, 0.47) * |  |
| MH | 18 417 (6.3) | 0.61 (0.59, 0.64) * | 0.38 (0.37, 0.39) * | 0.25 (0.24, 0.27) * |  | 0.64 (0.61, 0.67) * | 0.40 (0.38, 0.41) * | 0.27 (0.25, 0.28) * |  |
| **Coronary disease mortality** | | |  |  | 0.735 |  |  |  | 0.826 |
| MU | 2341 (2.1) | 1.0 (Reference) | 0.65 (0.59, 0.71) * | 0.42 (0.36, 0.48) * |  | 1.0 (Reference) | 0.64 (0.59, 0.71) * | 0.41 (0.36, 0.48) * |  |
| MH | 1992 (0.7) | 0.55 (0.49, 0.62) * | 0.35 (0.32, 0.39) * | 0.21 (0.19, 0.25) * |  | 0.58 (0.52, 0.65) * | 0.37 (0.34, 0.41) * | 0.23 (0.20, 0.26) * |  |
| **Myocardial infarction** | |  |  |  | 0.647 |  |  |  | 0.633 |
| MU | 9166 (7.6) | 1.0 (Reference) | 0.73 (0.70, 0.77) * | 0.54 (0.51, 0.58) * |  | 1.0 (Reference) | 0.73 (0.70, 0.77) * | 0.54 (0.51, 0.58) * |  |
| MH | 7837 (2.7) | 0.51 (0.48, 0.54) * | 0.36 (0.34, 0.38) * | 0.28 (0.26, 0.30) * |  | 0.54 (0.50, 0.57) * | 0.38 (0.36, 0.40) * | 0.29 (0.27, 0.31) * |  |
| **Myocardial infarction mortality** | |  |  |  | 0.432 |  |  |  | 0.432 |
| MU | 952 (0.8) | 1.0 (Reference) | 0.90 (0.78, 1.05) | 0.62 (0.50, 0.76) * |  | 1.0 (Reference) | 0.90 (0.77, 1.05) | 0.61 (0.49, 0.76) * |  |
| MH | 761 (0.3) | 0.59 (0.49, 0.72) * | 0.46 (0.39, 0.54) * | 0.32 (0.25, 0.40) * |  | 0.63 (0.52, 0.77) * | 0.49 (0.42, 0.58) * | 0.34 (0.27, 0.42) * |  |
| **Stroke** |  |  |  |  | 0.278 |  |  |  | 0.331 |
| MU | 4820 (3.9) | 1.0 (Reference) | 0.94 (0.88, 1.01) | 0.85 (0.77, 0.93) * |  | 1.0 (Reference) | 0.94 (0.88, 1.01) | 0.85 (0.77, 0.93) * |  |
| MH | 6053 (2.0) | 0.74 (0.69, 0.81) * | 0.66 (0.62, 0.71) * | 0.63 (0.58, 0.69) * |  | 0.77 (0.71, 0.83) * | 0.68 (0.64, 0.73) * | 0.65 (0.60, 0.71) * |  |
| **Stroke mortality** |  |  |  |  | 0.383 |  |  |  | 0.413 |
| MU | 1077 (0.9) | 1.0 (Reference) | 0.91 (0.78, 1.06) | 0.74 (0.61, 0.89) * |  | 1.0 (Reference) | 0.91 (0.78, 1.05) | 0.74 (0.61, 0.89) * |  |
| MH | 1192 (0.4) | 0.66 (0.56, 0.79) * | 0.59 (0.51, 0.68) * | 0.56 (0.46, 0.67) * |  | 0.69 (0.58, 0.83) * | 0.61 (0.53, 0.71) * | 0.58 (0.48, 0.70) * |  |
| **Heart failure** |  |  |  |  | 0.294 |  |  |  | 0.272 |
| MU | 8273 (6.7) | 1.0 (Reference) | 0.82 (0.78, 0.87) * | 0.74 (0.69, 0.79) * |  | 1.0 (Reference) | 0.82 (0.78, 0.86) | 0.73 (0.69, 0.79) * |  |
| MH | 6684 (2.3) | 0.47 (0.44, 0.50) * | 0.41 (0.39, 0.43) * | 0.36 (0.34, 0.39) * |  | 0.50 (0.46, 0.53) * | 0.43 (0.41, 0.46) * | 0.39 (0.36, 0.41) * |  |
| **Heart failure mortality** | |  |  |  | 0.766 |  |  |  | 0.747 |
| MU | 1316 (1.1) | 1.0 (Reference) | 0.81 (0.71, 0.92) * | 0.71 (0.60, 0.84) * |  | 1.0 (Reference) | 0.80 (0.71, 0.92) * | 0.70 (0.59, 0.83) * |  |
| MH | 822 (0.3) | 0.40 (0.34, 0.49) * | 0.35 (0.30, 0.40) * | 0.31 (0.26, 0.38) * |  | 0.43 (0.36, 0.52) * | 0.38 (0.33, 0.44) * | 0.33 (0.27, 0.40) * |  |
| **Atrial fibrillation** |  |  |  |  | 0.001 |  |  |  | < 0.001 |
| MU | 13 359 (10.9) | 1.0 (Reference) | 0.54 (0.52, 0.56) * | 0.31 (0.29, 0.33) * |  | 1.0 (Reference) | 0.54 (0.52, 0.56) * | 0.31 (0.30, 0.33) * |  |
| MH | 16 128 (5.5) | 0.75 (0.72, 0.78) * | 0.38 (0.36, 0.39) * | 0.20 (0.19, 0.22) * |  | 0.77 (0.74, 0.80) * | 0.38 (0.37, 0.40) * | 0.21 (0.20, 0.22) * |  |
| **Atrial fibrillation mortality** | |  |  |  | 0.879 |  |  |  | 0.871 |
| MU | 677 (0.6) | 1.0 (Reference) | 0.55 (0.47, 0.65) * | 0.30 (0.23, 0.40) * |  | 1.0 (Reference) | 0.55 (0.47, 0.65) * | 0.30 (0.23, 0.40) * |  |
| MH | 562 (0.2) | 0.58 (0.48, 0.70) * | 0.31 (0.26, 0.37) * | 0.16 (0.12, 0.21) * |  | 0.60 (0.50, 0.74) * | 0.32 (0.27, 0.38) * | 0.17 (0.12, 0.22) * |  |

Note, MH, metabolically healthy; MU, metabolically unhealthy; PRS, polygenic risk score; the PRSs presented are specifically used based on corresponding outcomes; Model 1 was adjusted for age, sex, race, Townsend Deprivation Index, annual household income, education attainment, 22 assessment centers, and the first 5 principal components of ancestry. Model 2 was further adjusted for family history of diabetes, family history of high blood pressure, and lifestyle factors including sleep duration, healthy diet, physical activity, smoking status, and alcohol intake frequency, based on Model 1; **^a^** likelihood tests was applied to test the significance of interaction term by comparing the model with and without the interaction term; **P* < 0.05.

**Table S6** **Combined effects of BMI status and PRSs on the risk of the cardiovascular outcomes and all-cause mortality**

| **Outcomes** | **Case (%)** | **Model 1** | | | | **Model 2** | | | |
| --- | --- | --- | --- | --- | --- | --- | --- | --- | --- |
|  |  | **High PRS** | **Moderate PRS** | **Low PRS** | ***P* for interaction ^a^** | **High PRS** | **Moderate PRS** | **Low PRS** | ***P* for interaction ^a^** |
| **All-cause mortality** |  |  |  |  | 0.038 |  |  |  | 0.025 |
| Obesity | 9573 (9.2) | 1.0 (Reference) | 0.96 (0.91, 1.01) | 0.87 (0.82, 0.93) * |  | 1.0 (Reference) | 0.96 (0.91, 1.01) | 0.87 (0.81, 0.93) * |  |
| Overweight | 13439 (7.2) | 0.78 (0.74, 0.83) * | 0.73 (0.70, 0.77) * | 0.66 (0.63, 0.70) * |  | 0.80 (0.75, 0.84) * | 0.74 (0.71, 0.78) * | 0.68 (0.64, 0.72) * |  |
| Normal weight | 8871 (6.0) | 0.82 (0.77, 0.88) * | 0.74 (0.70, 0.78) * | 0.74 (0.69, 0.79) * |  | 0.83 (0.78, 0.89) * | 0.75 (0.72, 0.79) * | 0.76 (0.71, 0.81) * |  |
| **CVD morbidity** |  |  |  |  | < 0.001 |  |  |  | < 0.001 |
| Obesity | 21785 (20.2) | 1.0 (Reference) * | 0.78 (0.75, 0.80) * | 0.62 (0.60, 0.65) * |  | 1.0 (Reference) | 0.78 (0.75, 0.80) | 0.63 (0.60, 0.65) * |  |
| Overweight | 29452 (15.1) | 0.74 (0.71, 0.76) * | 0.53 (0.52, 0.55) * | 0.40 (0.39, 0.42) * |  | 0.75 (0.73, 0.78) * | 0.55 (0.53, 0.57) * | 0.42 (0.40, 0.43) * |  |
| Normal weight | 16023 (10.5) | 0.60 (0.57, 0.62) * | 0.44 (0.42, 0.45) * | 0.35 (0.34, 0.37) * |  | 0.61 (0.59, 0.64) * | 0.45 (0.44, 0.47) * | 0.37 (0.35, 0.39) * |  |
| **CVD mortality** |  |  |  |  | 0.299 |  |  |  | 0.352 |
| Obesity | 2836 (2.6) | 1.0 (Reference) | 0.70 (0.65, 0.77) * | 0.55 (0.49, 0.62) * |  | 1.0 (Reference) | 0.70 (0.65, 0.77) * | 0.55 (0.49, 0.62) * |  |
| Overweight | 3368 (1.7) | 0.65 (0.59, 0.72) * | 0.46 (0.42, 0.50) * | 0.33 (0.30, 0.37) * |  | 0.67 (0.61, 0.74) * | 0.48 (0.44, 0.52) * | 0.34 (0.31, 0.38) * |  |
| Normal weight | 1929 (1.3) | 0.67 (0.60, 0.75) * | 0.43 (0.39, 0.47) * | 0.35 (0.30, 0.40) * |  | 0.69 (0.61, 0.77) * | 0.44 (0.40, 0.48) * | 0.36 (0.32, 0.41) * |  |
| **Coronary disease** |  |  |  |  | < 0.001 |  |  |  | < 0.001 |
| Obesity | 13204 (12) | 1.0 (Reference) | 0.68 (0.65, 0.71) * | 0.46 (0.44, 0.49) * |  | 1.0 (Reference) | 0.68 (0.66, 0.71) * | 0.47 (0.44, 0.49) * |  |
| Overweight | 17711 (9) | 0.77 (0.74, 0.80) * | 0.48 (0.46, 0.49) * | 0.30 (0.28, 0.32) * |  | 0.80 (0.76, 0.83) * | 0.49 (0.48, 0.51) * | 0.31 (0.30, 0.33) * |  |
| Normal weight | 8543 (5.5) | 0.58 (0.55, 0.61) * | 0.36 (0.34, 0.37) * | 0.23 (0.22, 0.25) * |  | 0.61 (0.58, 0.64) * | 0.37 (0.36, 0.39) * | 0.25 (0.23, 0.26) * |  |
| **Coronary disease mortality** |  |  |  |  | 0.532 |  |  |  | 0.556 |
| Obesity | 1781 (1.6) | 1.0 (Reference) | 0.62 (0.56, 0.69) * | 0.40 (0.35, 0.47) * |  | 1.0 (Reference) | 0.62 (0.56, 0.69) * | 0.40 (0.34, 0.47) * |  |
| Overweight | 2087 (1.1) | 0.66 (0.59, 0.75) * | 0.41 (0.37, 0.45) * | 0.23 (0.19, 0.26) * |  | 0.68 (0.61, 0.77) * | 0.42 (0.38, 0.47) * | 0.24 (0.20, 0.27) * |  |
| Normal weight | 1024 (0.7) | 0.56 (0.49, 0.65) * | 0.35 (0.31, 0.40) * | 0.23 (0.19, 0.27) * |  | 0.58 (0.50, 0.67) * | 0.36 (0.32, 0.41) * | 0.24 (0.19, 0.28) * |  |
| **Myocardial infarction** |  |  |  |  | 0.209 |  |  |  | 0.151 |
| Obesity | 6693 (5.8) | 1.0 (Reference) | 0.74 (0.70, 0.79) * | 0.56 (0.51, 0.60) * |  | 1.0 (Reference) | 0.74 (0.70, 0.79) * | 0.56 (0.52, 0.60) * |  |
| Overweight | 8728 (4.3) | 0.75 (0.71, 0.80) * | 0.52 (0.50, 0.55) * | 0.37 (0.34, 0.40) * |  | 0.79 (0.74, 0.84) * | 0.55 (0.52, 0.58) * | 0.38 (0.36, 0.41) * |  |
| Normal weight | 3895 (2.5) | 0.56 (0.52, 0.60) * | 0.39 (0.37, 0.42) * | 0.29 (0.26, 0.32) * |  | 0.59 (0.55, 0.64) * | 0.41 (0.39, 0.44) * | 0.30 (0.27, 0.33) * |  |
| **Myocardial infarction mortality** |  |  |  |  | 0.300 |  |  |  | 0.290 |
| Obesity | 710 (0.6) | 1.0 (Reference) | 0.72 (0.60, 0.85) * | 0.52 (0.41, 0.66) * |  | 1.0 (Reference) | 0.71 (0.60, 0.85) * | 0.52 (0.41, 0.67) * |  |
| Overweight | 812 (0.4) | 0.54 (0.44, 0.66) * | 0.49 (0.42, 0.58) * | 0.31 (0.24, 0.39) * |  | 0.56 (0.46, 0.69) * | 0.51 (0.43, 0.61) * | 0.32 (0.25, 0.40) * |  |
| Normal weight | 394 (0.3) | 0.48 (0.38, 0.62) * | 0.41 (0.34, 0.49) * | 0.30 (0.22, 0.40) * |  | 0.51 (0.40, 0.65) * | 0.43 (0.35, 0.52) * | 0.32 (0.23, 0.42) * |  |
| **Stroke** |  |  |  |  | 0.032 |  |  |  | 0.031 |
| Obesity | 3640 (3.1) | 1.0 (Reference) | 0.95 (0.88, 1.03) | 0.82 (0.74, 0.91) * |  | 1.0 (Reference) | 0.95 (0.88, 1.04) | 0.82 (0.74, 0.91) * |  |
| Overweight | 5420 (2.6) | 0.86 (0.79, 0.94) * | 0.76 (0.70, 0.82) * | 0.77 (0.70, 0.85) * |  | 0.88 (0.80, 0.97) * | 0.77 (0.71, 0.84) * | 0.79 (0.72, 0.86) * |  |
| Normal weight | 3252 (2.1) | 0.83 (0.75, 0.92) * | 0.74 (0.68, 0.81) * | 0.67 (0.60, 0.74) * |  | 0.85 (0.77, 0.94) * | 0.77 (0.70, 0.83) * | 0.69 (0.61, 0.76) * |  |
| **Stroke mortality** |  |  |  |  | 0.055 |  |  |  | 0.051 |
| Obesity | 744 (0.6) | 1.0 (Reference) | 0.94 (0.79, 1.13) | 0.72 (0.57, 0.92) * |  | 1.0 (Reference) | 0.94 (0.79, 1.13) | 0.72 (0.57, 0.91) * |  |
| Overweight | 1077 (0.5) | 0.90 (0.74, 1.10) | 0.70 (0.59, 0.83) * | 0.73 (0.60, 0.90) * |  | 0.93 (0.76, 1.13) | 0.72 (0.60, 0.86) * | 0.76 (0.61, 0.93) * |  |
| Normal weight | 748 (0.5) | 0.87 (0.70, 1.09) | 0.86 (0.72, 1.03) | 0.74 (0.59, 0.94) * |  | 0.91 (0.72, 1.13) | 0.89 (0.74, 1.07) | 0.77 (0.61, 0.97) * |  |
| **Heart failure** |  |  |  |  | 0.793 |  |  |  | 0.782 |
| Obesity | 7132 (6.1) | 1.0 (Reference) | 0.85 (0.80, 0.90) * | 0.75 (0.70, 0.81) * |  | 1.0 (Reference) | 0.85 (0.80, 0.89) * | 0.75 (0.70, 0.81) * |  |
| Overweight | 6637 (3.2) | 0.52 (0.49, 0.56) * | 0.43 (0.41, 0.46) * | 0.38 (0.35, 0.41) * |  | 0.54 (0.51, 0.58) * | 0.45 (0.42, 0.47) * | 0.39 (0.37, 0.42) * |  |
| Normal weight | 3218 (2.0) | 0.40 (0.37, 0.44) * | 0.35 (0.33, 0.38) * | 0.31 (0.28, 0.34) * |  | 0.42 (0.39, 0.46) * | 0.37 (0.35, 0.40) * | 0.33 (0.30, 0.36) * |  |
| **Heart failure mortality** |  |  |  |  | 0.385 |  |  |  | 0.407 |
| Obesity | 1091 (0.9) | 1.0 (Reference) | 0.87 (0.75, 1.01) | 0.78 (0.65, 0.94) * |  | 1.0 (Reference) | 0.87 (0.75, 1.00) | 0.78 (0.64, 0.94) * |  |
| Overweight | 861 (0.4) | 0.48 (0.40, 0.58) * | 0.39 (0.34, 0.46) * | 0.30 (0.24, 0.37) * |  | 0.50 (0.41, 0.60) * | 0.41 (0.35, 0.48) * | 0.31 (0.25, 0.39) * |  |
| Normal weight | 462 (0.3) | 0.47 (0.37, 0.58) * | 0.35 (0.29, 0.42) * | 0.34 (0.27, 0.43) * |  | 0.49 (0.39, 0.62) * | 0.37 (0.31, 0.44) * | 0.36 (0.28, 0.46) * |  |
| **Atrial fibrillation** |  |  |  |  | 0.002 |  |  |  | 0.002 |
| Obesity | 11941 (10.3) | 1.0 (Reference) | 0.55 (0.53, 0.57) * | 0.31 (0.29, 0.33) * |  | 1.0 (Reference) | 0.55 (0.53, 0.57) * | 0.31 (0.29, 0.33) * |  |
| Overweight | 14007 (6.9) | 0.66 (0.63, 0.69) * | 0.33 (0.32, 0.34) * | 0.19 (0.18, 0.20) * |  | 0.66 (0.64, 0.69) * | 0.34 (0.32, 0.35) * | 0.19 (0.18, 0.20) * |  |
| Normal weight | 7565 (4.8) | 0.56 (0.54, 0.59) * | 0.29 (0.28, 0.31) * | 0.15 (0.14, 0.16) * |  | 0.57 (0.55, 0.60) * | 0.30 (0.29, 0.31) * | 0.15 (0.14, 0.16) * |  |
| **Atrial fibrillation mortality** |  |  |  |  | < 0.001 |  |  |  | < 0.001 |
| Obesity | 592 (0.5) | 1.0 (Reference) | 0.55 (0.46, 0.65) * | 0.31 (0.23, 0.42) * |  | 1.0 (Reference) | 0.55 (0.46, 0.66) * | 0.32 (0.24, 0.42) * |  |
| Overweight | 532 (0.3) | 0.50 (0.41, 0.61) * | 0.26 (0.22, 0.31) * | 0.18 (0.13, 0.23) * |  | 0.51 (0.42, 0.63) * | 0.27 (0.22, 0.32) * | 0.18 (0.14, 0.24) * |  |
| Normal weight | 293 (0.2) | 0.52 (0.41, 0.66) * | 0.27 (0.22, 0.34) * | 0.05 (0.03, 0.10) * |  | 0.54 (0.43, 0.69) * | 0.29 (0.24, 0.35) * | 0.06 (0.03, 0.10) * |  |

Note, BMI, body mass index; PRS, polygenic risk score; the PRSs presented are specifically used based on corresponding outcomes; Model 1 is adjusted for age, sex, race, Townsend Deprivation Index, annual household income, education attainment, 22 assessment centers, and the first 5 principal components of ancestry. Model 2 is further adjusted for family history of diabetes, family history of high blood pressure, and lifestyle factors including sleep duration, healthy diet, physical activity, smoking status, and alcohol intake frequency, based on Model 1; **^a^** likelihood tests was applied to test the significance of interaction term by comparing the model with and without the interaction term; **P* < 0.05.

**Table S7 Combined effects of metabolic status (MH status: < 2 abnormal components) and PRSs on the risk of cardiovascular outcomes and all-cause mortality**

| **Outcomes** | **Case (%)** | **Model 1** | | | | **Model 2** | | | |
| --- | --- | --- | --- | --- | --- | --- | --- | --- | --- |
|  |  | **High PRS** | **Moderate PRS** | **Low PRS** | ***P* for interaction ^a^** | **High PRS** | **Moderate PRS** | **Low PRS** | ***P* for interaction ^a^** |
| **All-cause mortality** | |  |  |  | 0.508 |  |  |  | 0.495 |
| MU | 18 661 (9.3) | 1.0 (Reference) | 0.95 (0.92, 0.98) * | 0.89 (0.85, 0.93) * |  | 1.0 (Reference) | 0.95 (0.91, 0.98) | 0.89 (0.85, 0.93) |  |
| MH | 9686 (5.2) | 0.80 (0.76, 0.85) * | 0.73 (0.70, 0.76) * | 0.70 (0.67, 0.74) * |  | 0.83 (0.79, 0.88) | 0.76 (0.73, 0.79) | 0.73 (0.69, 0.77) |  |
| **Cardiovascular events** | |  |  |  | 0.160 |  |  |  | 0.124 |
| MU | 40 205 (19.3) | 1.0 (Reference) | 0.74 (0.72, 0.76) * | 0.58 (0.56, 0.60) * |  | 1.0 (Reference) | 0.74 (0.72, 0.76) | 0.58 (0.57, 0.60) |  |
| MH | 19 024 (10.0) | 0.65 (0.62, 0.67) * | 0.49 (0.48, 0.51) * | 0.40 (0.38, 0.41) * |  | 0.67 (0.64, 0.69) | 0.51 (0.50, 0.52) | 0.41 (0.40, 0.43) |  |
| **Cardiovascular mortality** | |  |  |  | 0.277 |  |  |  | 0.309 |
| MU | 5267 (2.5) | 1.0 (Reference) | 0.72 (0.67, 0.76) * | 0.57 (0.52, 0.62) * |  | 1.0 (Reference) | 0.71 (0.67, 0.76) | 0.57 (0.52, 0.62) |  |
| MH | 1953 (1.0) | 0.68 (0.61, 0.75) * | 0.44 (0.41, 0.48) * | 0.34 (0.30, 0.39) * |  | 0.71 (0.64, 0.79) | 0.46 (0.43, 0.50) | 0.37 (0.32, 0.41) |  |
| **Coronary disease** | |  |  |  | 0.044 |  |  |  | 0.045 |
| MU | 25 034 (11.8) | 1.0 (Reference) | 0.65 (0.63, 0.67) * | 0.42 (0.40, 0.44) * |  | 1.0 (Reference) | 0.65 (0.63, 0.67) | 0.42 (0.40, 0.44) |  |
| MH | 9690 (5.0) | 0.57 (0.55, 0.60) * | 0.36 (0.35, 0.37) * | 0.25 (0.24, 0.27) * |  | 0.60 (0.57, 0.62) | 0.38 (0.36, 0.39) | 0.26 (0.25, 0.28) |  |
| **Coronary disease mortality** | |  |  |  | 0.754 |  |  |  | 0.640 |
| MU | 3362 (1.6) | 1.0 (Reference) | 0.64 (0.60, 0.69) * | 0.39 (0.35, 0.44) * |  | 1.0 (Reference) | 0.64 (0.59, 0.69) | 0.39 (0.35, 0.44) |  |
| MH | 971 (0.5) | 0.53 (0.46, 0.61) * | 0.33 (0.30, 0.37) * | 0.22 (0.18, 0.26) * |  | 0.55 (0.48, 0.64) | 0.35 (0.31, 0.38) | 0.23 (0.19, 0.27) |  |
| **Myocardial infarction** | |  |  |  | 0.847 |  |  |  | 0.809 |
| MU | 13 048 (5.9) | 1.0 (Reference) | 0.72 (0.70, 0.75) * | 0.54 (0.51, 0.57) * |  | 1.0 (Reference) | 0.72 (0.69, 0.75) | 0.54 (0.51, 0.57) |  |
| MH | 3955 (2.0) | 0.50 (0.46, 0.53) * | 0.35 (0.34, 0.37) * | 0.27 (0.25, 0.29) * |  | 0.53 (0.49, 0.57) | 0.37 (0.35, 0.39) | 0.28 (0.26, 0.31) |  |
| **Myocardial infarction mortality** | |  |  |  | 0.042 |  |  |  | 0.038 |
| MU | 1358 (0.6) | 1.0 (Reference) | 0.87 (0.76, 0.99) * | 0.54 (0.45, 0.64) * |  | 1.0 (Reference) | 0.87 (0.76, 0.99) | 0.53 (0.44, 0.64) |  |
| MH | 355 (0.2) | 0.50 (0.39, 0.64) * | 0.38 (0.31, 0.45) * | 0.35 (0.27, 0.46) * |  | 0.54 (0.42, 0.68) | 0.40 (0.33, 0.48) | 0.37 (0.29, 0.49) |  |
| **Stroke** |  |  |  |  | 0.594 |  |  |  | 0.617 |
| MU | 7353 (3.3) | 1.0 (Reference) | 0.92 (0.87, 0.97) * | 0.84 (0.78, 0.90) * |  | 1.0 (Reference) | 0.92 (0.87, 0.97) | 0.84 (0.78, 0.90) |  |
| MH | 3520 (1.8) | 0.77 (0.70, 0.84) * | 0.69 (0.64, 0.73) * | 0.66 (0.60, 0.73) * |  | 0.79 (0.73, 0.87) | 0.71 (0.67, 0.76) | 0.68 (0.62, 0.75) |  |
| **Stroke mortality** |  |  |  |  | 0.786 |  |  |  | 0.804 |
| MU | 1541 (0.7) | 1.0 (Reference) | 0.91 (0.80, 1.03) | 0.78 (0.66, 0.92) * |  | 1.0 (Reference) | 0.91 (0.80, 1.03) | 0.78 (0.66, 0.92) |  |
| MH | 728 (0.4) | 0.82 (0.68, 0.99) * | 0.71 (0.62, 0.82) * | 0.66 (0.54, 0.81) * |  | 0.86 (0.71, 1.04) | 0.75 (0.65, 0.87) | 0.69 (0.57, 0.85) |  |
| **Heart failure** |  |  |  |  | 0.302 |  |  |  | 0.283 |
| MU | 11 520 (5.1) | 1.0 (Reference) | 0.83 (0.79, 0.86) * | 0.74 (0.70, 0.78) * |  | 1.0 (Reference) | 0.82 (0.79, 0.86) | 0.74 (0.70, 0.78) |  |
| MH | 3437 (1.8) | 0.48 (0.45, 0.53) * | 0.43 (0.41, 0.46) * | 0.37 (0.34, 0.40) * |  | 0.51 (0.47, 0.56) | 0.46 (0.43, 0.48) | 0.39 (0.36, 0.43) |  |
| **Heart failure mortality** | |  |  |  | 0.990 |  |  |  | 0.992 |
| MU | 1736 (0.8) | 1.0 (Reference) | 0.82 (0.73, 0.92) * | 0.72 (0.62, 0.83) * |  | 1.0 (Reference) | 0.82 (0.73, 0.92) | 0.72 (0.62, 0.83) |  |
| MH | 402 (0.2) | 0.43 (0.34, 0.54) * | 0.36 (0.31, 0.43) * | 0.32 (0.25, 0.41) * |  | 0.47 (0.37, 0.59) | 0.39 (0.33, 0.46) | 0.34 (0.27, 0.44) |  |
| **Atrial fibrillation** | |  |  |  | 0.006 |  |  |  | 0.005 |
| MU | 20 280 (9.1) | 1.0 (Reference) | 0.53 (0.52, 0.55) * | 0.30 (0.29, 0.31) * |  | 1.0 (Reference) | 0.53 (0.52, 0.55) | 0.30 (0.29, 0.32) |  |
| MH | 9207 (4.8) | 0.78 (0.75, 0.81) * | 0.38 (0.37, 0.40) * | 0.21 (0.20, 0.22) * |  | 0.80 (0.76, 0.83) | 0.39 (0.38, 0.41) | 0.21 (0.20, 0.23) |  |
| **Atrial fibrillation mortality** | |  |  |  | 0.111 |  |  |  | 0.106 |
| MU | 943 (0.4) | 1.0 (Reference) | 0.55 (0.48, 0.63) * | 0.32 (0.26, 0.40) * |  | 1.0 (Reference) | 0.55 (0.48, 0.63) | 0.32 (0.26, 0.40) |  |
| MH | 296 (0.2) | 0.65 (0.52, 0.81) * | 0.33 (0.28, 0.40) * | 0.12 (0.08, 0.19) * |  | 0.68 (0.55, 0.85) | 0.35 (0.29, 0.42) | 0.13 (0.08, 0.20) |  |

Note, MH, metabolically healthy; MU, metabolically unhealthy; PRS, polygenic risk score; the PRSs presented are specifically used based on corresponding outcomes; Model 1 was adjusted for age, sex, race, Townsend Deprivation Index, annual household income, education attainment, 22 assessment centers, and the first 5 principal components of ancestry. Model 2 was further adjusted for family history of diabetes, family history of high blood pressure, and lifestyle factors including sleep duration, healthy diet, physical activity, smoking status, and alcohol intake frequency, based on Model 1; **^a^** likelihood tests was applied to test the significance of interaction term by comparing the model with and without the interaction term; **P* < 0.05.

**Table S8 Combined effects of metabolic status (MH status: < 1 abnormal components) and PRSs on the risk of cardiovascular outcomes and all-cause mortality**

| **Outcomes** | **Case (%)** | **Model 1** | | |  | **Model 2** | | | |
| --- | --- | --- | --- | --- | --- | --- | --- | --- | --- |
|  |  | **High PRS** | **Moderate PRS** | **Low PRS** | ***P* for interaction ^a^** | **High PRS** | **Moderate PRS** | **Low PRS** | ***P* for interaction ^a^** |
| **All-cause mortality** | |  |  |  | 0.391 |  |  |  | 0.340 |
| MU | 26 047 (8.1) | 1.0 (Reference) | 0.94 (0.91, 0.97) * | 0.88 (0.85, 0.91) * |  | 1.0 (Reference) | 0.94 (0.91, 0.97) * | 0.88 (0.85, 0.92) * |  |
| MH | 2300 (3.6) | 0.79 (0.71, 0.87) * | 0.70 (0.66, 0.74) * | 0.70 (0.64, 0.76) * |  | 0.80 (0.72, 0.89) * | 0.72 (0.67, 0.76) * | 0.72 (0.66, 0.79) * |  |
| **Cardiovascular events** | |  |  |  | 0.082 |  |  |  | 0.072 |
| MU | 55 025 (16.5) | 1.0 (Reference) | 0.74 (0.73, 0.76) * | 0.58 (0.57, 0.60) * |  | 1.0 (Reference) | 0.74 (0.73, 0.76) * | 0.59 (0.57, 0.60) * |  |
| MH | 4199 (6.3) | 0.56 (0.52, 0.60) * | 0.45 (0.43, 0.47) * | 0.36 (0.34, 0.38) * |  | 0.57 (0.53, 0.62) * | 0.47 (0.45, 0.49) * | 0.37 (0.35, 0.40) * |  |
| **Cardiovascular mortality** | |  |  |  | 0.406 |  |  |  | 0.373 |
| MU | 6870 (2.1) | 1.0 (Reference) | 0.70 (0.66, 0.74) * | 0.54 (0.50, 0.58) * |  | 1.0 (Reference) | 0.70 (0.66, 0.74) * | 0.54 (0.50, 0.59) * |  |
| MH | 350 (0.5) | 0.52 (0.41, 0.66) * | 0.35 (0.31, 0.41) * | 0.33 (0.26, 0.41) * |  | 0.54 (0.43, 0.68) * | 0.37 (0.32, 0.43) * | 0.35 (0.28, 0.44) * |  |
| **Coronary disease** | |  |  |  | 0.637 |  |  |  | 0.628 |
| MU | 32 714 (9.7) | 1.0 (Reference) | 0.64 (0.63, 0.66) * | 0.42 (0.40, 0.43) * |  | 1.0 (Reference) | 0.64 (0.63, 0.66) * | 0.42 (0.41, 0.44) * |  |
| MH | 2010 (3.0) | 0.50 (0.46, 0.55) * | 0.32 (0.30, 0.34) * | 0.22 (0.20, 0.24) * |  | 0.52 (0.47, 0.57) * | 0.33 (0.31, 0.35) * | 0.23 (0.21, 0.26) * |  |
| **Coronary disease mortality** | | |  |  | 0.026 |  |  |  | 0.024 |
| MU | 4167 (1.2) | 1.0 (Reference) | 0.64 (0.60, 0.68) * | 0.38 (0.34, 0.42) * |  | 1.0 (Reference) | 0.64 (0.59, 0.68) * | 0.38 (0.34, 0.42) * |  |
| MH | 166 (0.2) | 0.47 (0.35, 0.64) * | 0.24 (0.19, 0.30) * | 0.25 (0.18, 0.35) * |  | 0.49 (0.36, 0.66) * | 0.25 (0.20, 0.31) * | 0.27 (0.19, 0.37) * |  |
| **Myocardial infarction** | |  |  |  | 0.135 |  |  |  | 0.138 |
| MU | 16 276 (4.7) | 1.0 (Reference) | 0.72 (0.69, 0.74) * | 0.53 (0.50, 0.56) * |  | 1.0 (Reference) | 0.71 (0.69, 0.74) * | 0.53 (0.50, 0.55) * |  |
| MH | 727 (1.1) | 0.37 (0.32, 0.43) * | 0.29 (0.26, 0.32) * | 0.25 (0.21, 0.30) * |  | 0.39 (0.33, 0.45) * | 0.30 (0.27, 0.33) * | 0.26 (0.22, 0.31) * |  |
| **Myocardial infarction mortality** | | |  |  | 0.027 |  |  |  | 0.028 |
| MU | 1664 (0.5) | 1.0 (Reference) | 0.84 (0.75, 0.94) * | 0.55 (0.47, 0.65) * |  | 1.0 (Reference) | 0.84 (0.74, 0.94) * | 0.55 (0.46, 0.64) * |  |
| MH | 49 (0.1) | 0.25 (0.13, 0.49) * | 0.23 (0.15, 0.34) * | 0.37 (0.22, 0.63) * |  | 0.26 (0.14, 0.51) * | 0.24 (0.16, 0.35) * | 0.39 (0.23, 0.66) * |  |
| **Stroke** |  |  |  |  | 0.236 |  |  |  | 0.239 |
| MU | 10 163 (2.9) | 1.0 (Reference) | 0.90 (0.86, 0.95) * | 0.84 (0.79, 0.90) * |  | 1.0 (Reference) | 0.90 (0.86, 0.95) * | 0.84 (0.79, 0.90) * |  |
| MH | 710 (1.1) | 0.54 (0.45, 0.64) * | 0.57 (0.52, 0.63) * | 0.50 (0.42, 0.60) * |  | 0.55 (0.46, 0.66) * | 0.59 (0.53, 0.65) * | 0.51 (0.43, 0.61) * |  |
| **Stroke mortality** | |  |  |  | 0.273 |  |  |  | 0.276 |
| MU | 2133 (0.6) | 1.0 (Reference) | 0.88 (0.79, 0.98) * | 0.78 (0.68, 0.89) * |  | 1.0 (Reference) | 0.88 (0.79, 0.98) * | 0.78 (0.68, 0.89) * |  |
| MH | 136 (0.2) | 0.48 (0.31, 0.74) * | 0.61 (0.49, 0.77) * | 0.48 (0.32, 0.72) * |  | 0.50 (0.32, 0.77) * | 0.64 (0.51, 0.80) * | 0.50 (0.33, 0.75) * |  |
| **Heart failure** |  |  |  |  | 0.363 |  |  |  | 0.367 |
| MU | 14 266 (4) | 1.0 (Reference) | 0.83 (0.80, 0.87) * | 0.73 (0.70, 0.77) * |  | 1.0 (Reference) | 0.83 (0.80, 0.87) * | 0.74 (0.70, 0.77) * |  |
| MH | 691 (1) | 0.44 (0.37, 0.52) * | 0.41 (0.37, 0.45) * | 0.38 (0.32, 0.45) * |  | 0.46 (0.39, 0.54) * | 0.43 (0.38, 0.47) * | 0.40 (0.34, 0.47) * |  |
| **Heart failure mortality** | |  |  |  | 0.498 |  |  |  | 0.487 |
| MU | 2057 (0.6) | 1.0 (Reference) | 0.82 (0.74, 0.91) * | 0.72 (0.63, 0.83) * |  | 1.0 (Reference) | 0.82 (0.74, 0.91) * | 0.72 (0.63, 0.83) * |  |
| MH | 81 (0.1) | 0.51 (0.33, 0.79) * | 0.39 (0.29, 0.53) * | 0.24 (0.13, 0.44) * |  | 0.54 (0.35, 0.84) * | 0.42 (0.31, 0.56) * | 0.26 (0.14, 0.47) * |  |
| **Atrial fibrillation** | |  |  |  | 0.073 |  |  |  | 0.066 |
| MU | 27 402 (7.8) | 1.0 (Reference) | 0.52 (0.51, 0.54) * | 0.29 (0.28, 0.30) * |  | 1.0 (Reference) | 0.52 (0.51, 0.54) * | 0.29 (0.28, 0.30) * |  |
| MH | 2085 (3.1) | 0.77 (0.71, 0.83) * | 0.36 (0.34, 0.38) * | 0.21 (0.18, 0.24) * |  | 0.79 (0.73, 0.85) * | 0.37 (0.35, 0.39) * | 0.21 (0.19, 0.24) * |  |
| **Atrial fibrillation mortality** | |  |  |  | 0.416 |  |  |  | 0.402 |
| MU | 1197 (0.3) | 1.0 (Reference) | 0.55 (0.48, 0.62) * | 0.29 (0.24, 0.36) * |  | 1.0 (Reference) | 0.55 (0.49, 0.62) * | 0.30 (0.24, 0.36) * |  |
| MH | 42 (0.1) | 0.57 (0.35, 0.92) * | 0.22 (0.14, 0.34) * | 0.09 (0.03, 0.27) * |  | 0.60 (0.37, 0.97) * | 0.23 (0.15, 0.35) * | 0.09 (0.03, 0.28) * |  |

Note, MH, metabolically healthy; MU, metabolically unhealthy; PRS, polygenic risk score; the PRSs presented are specifically used based on corresponding outcomes; Model 1 was adjusted for age, sex, race, Townsend Deprivation Index, annual household income, education attainment, 22 assessment centers, and the first 5 principal components of ancestry. Model 2 was further adjusted for family history of diabetes, family history of high blood pressure, and lifestyle factors including sleep duration, healthy diet, physical activity, smoking status, and alcohol intake frequency, based on Model 1; **^a^** likelihood tests was applied to test the significance of interaction term by comparing the model with and without the interaction term; **P* < 0.05.

**Table S9 Transitions of metabolic status (MH status: < 3 abnormal components) from baseline to the second resurvey**

| MH status at baseline | MH status at the second resurvey, number of participants (%) | | |
| --- | --- | --- | --- |
|  | MH | MU | Total |
| MH | 8200 (85.0) | 1448 (15.0) | 9648 (100.0) |
| MU | 931 (26.1) | 2637 (73.9) | 3568 (100.0) |
| Total | 9131 (69.1) | 4085 (30.9) | 13 216 (100.0) |

Note, MH, metabolically healthy; MU, metabolically unhealthy

**Table S10** **Transitions of BMI status from baseline to the second survey**

| BMI status at baseline | BMI status at the second resurvey, number of participants (%) | | | |
| --- | --- | --- | --- | --- |
|  | Normal weight | Overweight | Obesity | Total |
| Normal weight | 6269 (84.7) | 1126 (15.2) | 8 (0.1) | 7403 (100.0) |
| Overweight | 984 (11.6) | 6704 (79.0) | 798 (9.4) | 8486 (100.0) |
| Obesity | 33 (0.8) | 683 (16.9) | 3317 (82.2) | 4033 (100.0) |
| Total | 7286 (36.6) | 8513 (42.7) | 4123 (20.7) | 19 922 (100.0) |

Note, BMI, body mass index

**Table S11 Combined effects of transitions in metabolic status (MH status: < 3 abnormal components) and PRSs on the risk of specific cardiovascular outcomes**

| **Outcomes** | **Case (%)** | **Model 1** | | | | **Model 2** | | | |
| --- | --- | --- | --- | --- | --- | --- | --- | --- | --- |
|  |  | **High PRS** | **Moderate PRS** | **Low PRS** | ***P* for interaction ^a^** | **High PRS** | **Moderate PRS** | **Low PRS** | ***P* for interaction ^a^** |
| **Coronary disease** | |  |  |  | 0.915 |  |  |  | 0.922 |
| MU throughout | 245 (11.2) | 1.0 (Reference) | 0.67 (0.50, 0.89) * | 0.44 (0.29, 0.68) * |  | 1.0 (Reference) | 0.67 (0.50, 0.89) * | 0.44 (0.28, 0.67) * |  |
| MH to MU | 123 (9.2) | 0.83 (0.54, 1.29) | 0.64 (0.46, 0.88) * | 0.47 (0.28, 0.78) * |  | 0.84 (0.54, 1.30) | 0.64 (0.46, 0.88) * | 0.47 (0.28, 0.78) * |  |
| MH throughout | 337 (4.2) | 0.53 (0.39, 0.73) * | 0.35 (0.27, 0.46) * | 0.21 (0.15, 0.31) * |  | 0.54 (0.39, 0.75) * | 0.36 (0.27, 0.47) * | 0.22 (0.15, 0.31) * |  |
| **Coronary disease mortality** | | |  |  | 0.278 |  |  |  | 0.262 |
| MU throughout | 43 (1.8) | 1.0 (Reference) | 0.90 (0.44, 1.84) | 0.70 (0.26, 1.91) |  | 1.0 (Reference) | 0.89 (0.44, 1.81) | 0.67 (0.25, 1.83) |  |
| MH to MU | 10 (0.7) | 0.72 (0.20, 2.61) | 0.47 (0.18, 1.21) | - |  | 0.75 (0.21, 2.71) | 0.46 (0.18, 1.20) | - |  |
| MH throughout | 32 (0.4) | 0.51 (0.20, 1.28) | 0.27 (0.13, 0.60) * | 0.36 (0.14, 0.91) * |  | 0.61 (0.24, 1.56) | 0.31 (0.14, 0.69) * | 0.43 (0.17, 1.09) |  |
| **Myocardial infarction** |  |  |  |  | 0.545 |  |  |  | 0.527 |
| MU throughout | 179 (7.2) | 1.0 (Reference) | 0.90 (0.63, 1.28) | 0.50 (0.29, 0.86) * |  | 1.0 (Reference) | 0.89 (0.62, 1.26) | 0.48 (0.28, 0.84) * |  |
| MH to MU | 64 (4.5) | 0.88 (0.51, 1.51) | 0.62 (0.40, 0.97) * | 0.36 (0.16, 0.80) * |  | 0.90 (0.52, 1.55) | 0.62 (0.40, 0.97) * | 0.35 (0.16, 0.79) * |  |
| MH throughout | 143 (1.7) | 0.38 (0.24, 0.60) * | 0.31 (0.21, 0.45) * | 0.28 (0.17, 0.46) * |  | 0.39 (0.24, 0.62) * | 0.32 (0.22, 0.46) * | 0.29 (0.17, 0.47) * |  |
| **Myocardial infarction mortality** | |  |  |  | 0.383 |  |  |  | 0.784 |
| MU throughout | 24 (0.9) | 1.0 (Reference) | 1.27 (0.41, 3.88) | 1.60 (0.42, 6.07) |  | 1.0 (Reference) | 1.25 (0.40, 3.85) | 1.52 (0.40, 5.78) |  |
| MH to MU | 1 (0.1) | - | 0.18 (0.02, 1.65) | - |  | - | 0.19 (0.02, 1.73) | - |  |
| MH throughout | 16 (0.2) | 0.50 (0.12, 2.08) | 0.34 (0.10, 1.16) | 0.34 (0.07, 1.59) |  | 0.57 (0.14, 2.38) | 0.40 (0.12, 1.37) | 0.41 (0.09, 1.89) |  |
| **Stroke** |  |  |  |  | 0.955 |  |  |  | 0.938 |
| MU throughout | 95 (3.6) | 1.0 (Reference) | 0.92 (0.55, 1.55) | 0.96 (0.51, 1.79) |  | 1.0 (Reference) | 0.90 (0.53, 1.51) | 0.95 (0.51, 1.79) |  |
| MH to MU | 31 (2.2) | 0.57 (0.21, 1.53) | 0.66 (0.35, 1.24) | 0.63 (0.25, 1.59) |  | 0.56 (0.21, 1.51) | 0.68 (0.36, 1.27) | 0.63 (0.25, 1.61) |  |
| MH throughout | 122 (1.5) | 0.66 (0.36, 1.20) | 0.56 (0.34, 0.94) * | 0.49 (0.26, 0.92) * |  | 0.69 (0.38, 1.26) | 0.59 (0.35, 0.99) * | 0.51 (0.27, 0.96) * |  |
| **Stroke mortality** |  |  |  |  | 0.410 |  |  |  | 0.414 |
| MU throughout | 17 (0.7) | 1.0 (Reference) | 0.35 (0.13, 0.97) | 0.25 (0.05, 1.23) |  | 1.0 (Reference) | 0.34 (0.12, 0.95) | 0.25 (0.05, 1.22) |  |
| MH to MU | 6 (0.4) | - | 0.40 (0.13, 1.29) | 0.29 (0.04, 2.41) |  | - | 0.41 (0.13, 1.33) | 0.30 (0.04, 2.44) |  |
| MH throughout | 26 (0.3) | 0.42 (0.14, 1.23) | 0.26 (0.10, 0.66) * | 0.29 (0.09, 0.93) * |  | 0.44 (0.15, 1.32) | 0.27 (0.10, 0.70) * | 0.30 (0.09, 0.98) * |  |
| **Heart failure** |  |  |  |  | 0.394 |  |  |  | 0.407 |
| MU throughout | 149 (5.7) | 1.0 (Reference) | 0.65 (0.44, 0.97) * | 0.93 (0.58, 1.49) |  | 1.0 (Reference) | 0.65 (0.44, 0.96) * | 0.91 (0.57, 1.45) |  |
| MH to MU | 48 (3.3) | 0.49 (0.24, 1.02) | 0.51 (0.31, 0.84) * | 0.66 (0.34, 1.27) |  | 0.50 (0.24, 1.04) | 0.52 (0.31, 0.85) * | 0.67 (0.35, 1.28) |  |
| MH throughout | 137 (1.7) | 0.33 (0.20, 0.56) * | 0.36 (0.24, 0.54) * | 0.35 (0.21, 0.58) * |  | 0.35 (0.21, 0.60) * | 0.38 (0.26, 0.57) * | 0.37 (0.23, 0.61) * |  |
| **Heart failure mortality** | |  |  |  | 0.014 |  |  |  | 0.004 |
| MU throughout | 27 (1.0) | 1.0 (Reference) | 0.82 (0.34, 1.97) | 0.30 (0.06, 1.45) |  | 1.0 (Reference) | 0.82 (0.34, 1.98) | 0.30 (0.06, 1.47) |  |
| MH to MU | 9 (0.6) | - | 0.53 (0.17, 1.69) | 1.27 (0.37, 4.37) |  | - | 0.55 (0.17, 1.75) | 1.31 (0.38, 4.52) |  |
| MH throughout | 13 (0.2) | 0.17 (0.04, 0.86) * | 0.20 (0.07, 0.57) * | 0.22 (0.06, 0.86) * |  | 0.18 (0.04, 0.91) * | 0.21 (0.07, 0.59) * | 0.23 (0.06, 0.90) * |  |
| **Atrial fibrillation** |  |  |  |  | 0.445 |  |  |  | 0.431 |
| MU throughout | 258 (10.3) | 1.0 (Reference) | 0.54 (0.41, 0.71) | 0.34 (0.23, 0.51) * |  | 1.0 (Reference) | 0.54 (0.41, 0.71) * | 0.34 (0.23, 0.51) * |  |
| MH to MU | 87 (6.2) | 0.74 (0.48, 1.15) | 0.39 (0.27, 0.55) * | 0.18 (0.09, 0.37) * |  | 0.75 (0.48, 1.16) | 0.39 (0.27, 0.56) * | 0.19 (0.09, 0.37) * |  |
| MH throughout | 355 (4.4) | 0.71 (0.53, 0.95) * | 0.36 (0.27, 0.47) * | 0.14 (0.09, 0.21) * |  | 0.73 (0.54, 0.97) * | 0.37 (0.28, 0.48) * | 0.14 (0.09, 0.22) * |  |
| **Atrial fibrillation mortality** | |  |  |  | 0.013 |  |  |  | 0.003 |
| MU throughout | 12 (0.5) | 1.0 (Reference) | 1.37 (0.28, 6.80) | 1.65 (0.27, 10.19) |  | 1.0 (Reference) | 1.33 (0.26, 6.66) | 1.64 (0.26, 10.21) |  |
| MH to MU | 4 (0.3) | - | 1.94 (0.34, 11.02) | - |  | - | 2.04 (0.35, 11.79) | - |  |
| MH throughout | 10 (0.1) | 1.54 (0.27, 8.84) | 0.75 (0.14, 3.91) | - |  | 1.77 (0.30, 10.33) | 0.84 (0.16, 4.44) | - |  |

Note, MH, metabolically healthy; MU, metabolically unhealthy; PRS, polygenic risk score; the PRSs presented are specifically used based on corresponding outcomes; Model 1 was adjusted for age, sex, race, Townsend Deprivation Index, annual household income, education attainment, 22 assessment centers, and the first 5 principal components of ancestry. Model 2 was further adjusted for family history of diabetes, family history of high blood pressure, and lifestyle factors including sleep duration, healthy diet, physical activity, smoking status, and alcohol intake frequency, based on Model 1; **^a^** likelihood tests was applied to test the significance of interaction term by comparing the model with and without the interaction term; **P* < 0.05.

**Table S12** **Combined effects of transitions in BMI status and PRSs on the risk of cardiovascular outcomes and all-cause mortality**

| **Outcomes** | **Case (%)** | **Model 1** | | | | **Model 2** | | | |
| --- | --- | --- | --- | --- | --- | --- | --- | --- | --- |
|  |  | **High PRS** | **Moderate PRS** | **Low PRS** | ***P* for interaction ^a^** | **High PRS** | **Moderate PRS** | **Low PRS** | ***P* for interaction ^a^** |
| **All-cause mortality** |  |  |  |  | 0.709 |  |  |  | 0.711 |
| Obesity throughout | 210 (7.1) | 1.0 (Reference) | 1.16 (0.80, 1.68) | 1.09 (0.69, 1.73) |  | 1.0 (Reference) | 1.16 (0.80, 1.69) | 1.10 (0.69, 1.74) |  |
| Overweight to obesity | 35 (4.9) | 0.98 (0.45, 2.11) | 0.84 (0.50, 1.41) | 0.35 (0.11, 1.13) |  | 0.92 (0.42, 1.98) | 0.83 (0.50, 1.41) | 0.34 (0.10, 1.10) |  |
| Overweight throughout | 315 (5.1) | 0.84 (0.55, 1.27) | 0.77 (0.54, 1.11) | 0.72 (0.47, 1.09) |  | 0.85 (0.56, 1.29) | 0.78 (0.54, 1.12) | 0.73 (0.48, 1.10) |  |
| Normal weight to overweight | 41 (3.9) | 0.80 (0.37, 1.72) | 0.63 (0.38, 1.05) | 0.60 (0.28, 1.29) |  | 0.79 (0.37, 1.71) | 0.63 (0.37, 1.05) | 0.61 (0.28, 1.31) |  |
| Normal weight throughout | 233 (3.9) | 0.63 (0.39, 1.01) | 0.73 (0.50, 1.06) | 0.77 (0.50, 1.18) |  | 0.65 (0.41, 1.04) | 0.75 (0.51, 1.08) | 0.79 (0.52, 1.22) |  |
| **CVD morbidity** |  |  |  |  | 0.086 |  |  |  | 0.087 |
| Obesity throughout | 446 (15.6) | 1.0 (Reference) | 0.77 (0.61, 0.97) * | 0.69 (0.51, 0.93) * |  | 1.0 (Reference) | 0.77 (0.61, 0.96) * | 0.71 (0.52, 0.96) * |  |
| Overweight to obesity | 100 (14.3) | 1.16 (0.76, 1.77) | 0.73 (0.53, 1.00) | 0.36 (0.19, 0.69) * |  | 1.13 (0.75, 1.73) | 0.74 (0.54, 1.02) | 0.36 (0.19, 0.70) * |  |
| Overweight throughout | 748 (12.3) | 0.80 (0.62, 1.02) | 0.55 (0.44, 0.69) * | 0.42 (0.32, 0.55) * |  | 0.82 (0.64, 1.05) | 0.57 (0.46, 0.71) * | 0.43 (0.33, 0.56) * |  |
| Normal weight to overweight | 102 (9.8) | 0.61 (0.37, 1.00) | 0.52 (0.38, 0.71) * | 0.38 (0.23, 0.64) * |  | 0.63 (0.38, 1.02) | 0.54 (0.39, 0.73) * | 0.39 (0.24, 0.66) * |  |
| Normal weight throughout | 525 (8.8) | 0.53 (0.40, 0.70) * | 0.53 (0.43, 0.66) * | 0.31 (0.23, 0.42) * |  | 0.55 (0.42, 0.73) * | 0.55 (0.44, 0.69) * | 0.33 (0.24, 0.44) * |  |
| **CVD mortality** |  |  |  |  | 0.864 |  |  |  | 0.862 |
| Obesity throughout | 60 (2) | 1.0 (Reference) | 0.90 (0.48, 1.69) | 0.62 (0.26, 1.50) |  | 1.0 (Reference) | 0.90 (0.48, 1.69) | 0.63 (0.26, 1.52) |  |
| Overweight to obesity | 10 (1.3) | 0.62 (0.14, 2.75) | 0.69 (0.28, 1.74) | 0.32 (0.04, 2.46) |  | 0.56 (0.13, 2.51) | 0.70 (0.28, 1.77) | 0.30 (0.04, 2.33) |  |
| Overweight throughout | 84 (1.3) | 0.83 (0.42, 1.65) | 0.55 (0.30, 1.02) | 0.40 (0.19, 0.87) * |  | 0.88 (0.44, 1.75) | 0.58 (0.31, 1.08) | 0.42 (0.19, 0.91) |  |
| Normal weight to overweight | 10 (0.9) | 0.84 (0.24, 2.95) | 0.42 (0.16, 1.12) | 0.22 (0.03, 1.69) |  | 0.83 (0.24, 2.92) | 0.43 (0.16, 1.14) | 0.23 (0.03, 1.73) |  |
| Normal weight throughout | 54 (0.9) | 0.37 (0.15, 0.93) * | 0.54 (0.28, 1.02) | 0.39 (0.17, 0.90) * |  | 0.40 (0.16, 1.00) | 0.57 (0.30, 1.08) | 0.42 (0.18, 0.97) * |  |
| **Coronary disease** | |  |  |  | 0.777 |  |  |  | 0.830 |
| Obesity throughout | 243 (8.2) | 1.0 (Reference) | 0.61 (0.45, 0.81) * | 0.41 (0.28, 0.62) * |  | 1.0 (Reference) | 0.61 (0.45, 0.81) * | 0.41 (0.27, 0.61) * |  |
| Overweight to obesity | 57 (7.8) | 1.12 (0.66, 1.88) | 0.61 (0.41, 0.93) * | 0.21 (0.08, 0.58) * |  | 1.08 (0.64, 1.81) | 0.62 (0.41, 0.93) * | 0.22 (0.08, 0.60) * |  |
| Overweight throughout | 415 (6.6) | 0.70 (0.51, 0.95) * | 0.49 (0.37, 0.64) * | 0.28 (0.20, 0.41) * |  | 0.71 (0.52, 0.96) * | 0.50 (0.38, 0.65) * | 0.29 (0.20, 0.41) * |  |
| Normal weight to overweight | 58 (5.5) | 0.68 (0.39, 1.17) | 0.43 (0.28, 0.65) * | 0.32 (0.16, 0.65) * |  | 0.68 (0.39, 1.17) | 0.44 (0.29, 0.67) * | 0.33 (0.16, 0.66) * |  |
| Normal weight throughout | 278 (4.6) | 0.53 (0.37, 0.74) * | 0.42 (0.32, 0.56) * | 0.25 (0.17, 0.37) * |  | 0.54 (0.39, 0.77) * | 0.43 (0.33, 0.58) * | 0.26 (0.17, 0.38) * |  |
| **Coronary disease mortality** | | |  |  | 0.465 |  |  |  | 0.476 |
| Obesity throughout | 35 (1.1) | 1.0 (Reference) | 0.94 (0.42, 2.11) | 0.57 (0.19, 1.75) |  | 1.0 (Reference) | 0.96 (0.43, 2.16) | 0.56 (0.18, 1.72) |  |
| Overweight to obesity | 7 (0.9) | 1.64 (0.43, 6.24) | 0.70 (0.21, 2.32) | - |  | 1.53 (0.40, 5.83) | 0.66 (0.20, 2.21) | - |  |
| Overweight throughout | 45 (0.7) | 1.11 (0.48, 2.58) | 0.44 (0.20, 1.00) | 0.35 (0.12, 1.00) |  | 1.20 (0.51, 2.80) | 0.47 (0.21, 1.07) | 0.37 (0.13, 1.07) |  |
| Normal weight to overweight | 6 (0.6) | 0.46 (0.06, 3.69) | 0.51 (0.15, 1.70) | 0.36 (0.05, 2.92) |  | 0.48 (0.06, 3.89) | 0.51 (0.15, 1.70) | 0.38 (0.05, 3.05) |  |
| Normal weight throughout | 25 (0.4) | 0.35 (0.10, 1.18) | 0.48 (0.21, 1.13) | 0.32 (0.09, 1.06) |  | 0.39 (0.11, 1.31) | 0.53 (0.23, 1.25) | 0.34 (0.10, 1.13) |  |
| **Myocardial infarction** |  |  |  |  | 0.549 |  |  |  | 0.541 |
| Obesity throughout | 156 (4.9) | 1.0 (Reference) | 0.62 (0.43, 0.90) * | 0.51 (0.31, 0.84) * |  | 1.0 (Reference) | 0.62 (0.43, 0.89) * | 0.51 (0.31, 0.84) * |  |
| Overweight to obesity | 34 (4.4) | 0.71 (0.35, 1.46) | 0.67 (0.40, 1.12) | 0.28 (0.09, 0.89) * |  | 0.72 (0.35, 1.47) | 0.65 (0.39, 1.10) | 0.28 (0.09, 0.89) * |  |
| Overweight throughout | 207 (3.2) | 0.43 (0.29, 0.66) * | 0.41 (0.29, 0.58) * | 0.33 (0.21, 0.52) * |  | 0.44 (0.29, 0.67) * | 0.42 (0.29, 0.59) * | 0.34 (0.21, 0.53) * |  |
| Normal weight to overweight | 30 (2.7) | 0.57 (0.27, 1.22) | 0.45 (0.27, 0.77) | 0.13 (0.03, 0.52) * |  | 0.58 (0.27, 1.23) | 0.46 (0.27, 0.78) * | 0.13 (0.03, 0.52) * |  |
| Normal weight throughout | 128 (2.1) | 0.40 (0.25, 0.63) * | 0.35 (0.24, 0.51) * | 0.24 (0.14, 0.42) * |  | 0.41 (0.26, 0.65) * | 0.36 (0.25, 0.53) * | 0.24 (0.14, 0.43) * |  |
| **Myocardial infarction mortality** |  |  |  |  | 0.604 |  |  |  | 0.498 |
| Obesity throughout | 16 (0.5) | 1.0 (Reference) | 0.50 (0.16, 1.55) | 0.59 (0.14, 2.46) |  | 1.0 (Reference) | 0.50 (0.16, 1.53) | 0.58 (0.14, 2.46) |  |
| Overweight to obesity | 4 (0.5) | 0.75 (0.09, 6.50) | 0.82 (0.20, 3.48) | - |  | 0.76 (0.09, 6.61) | 0.73 (0.17, 3.11) | - |  |
| Overweight throughout | 19 (0.3) | 0.46 (0.13, 1.61) | 0.27 (0.09, 0.80) * | 0.47 (0.13, 1.65) |  | 0.47 (0.14, 1.64) | 0.28 (0.09, 0.84) * | 0.49 (0.14, 1.72) |  |
| Normal weight to overweight | 6 (0.5) | 0.67 (0.08, 5.78) | 1.05 (0.30, 3.65) | - |  | 0.64 (0.07, 5.49) | 1.06 (0.30, 3.70) | - |  |
| Normal weight throughout | 9 (0.1) | 0.37 (0.09, 1.57) | 0.20 (0.06, 0.70) * | 0.13 (0.02, 1.12) |  | 0.41 (0.10, 1.73) | 0.21 (0.06, 0.75) * | 0.13 (0.02, 1.15) |  |
| **Stroke** |  |  |  |  | 0.855 |  |  |  | 0.861 |
| Obesity throughout | 83 (2.5) | 1.0 (Reference) | 0.74 (0.43, 1.26) | 0.98 (0.52, 1.84) |  | 1.0 (Reference) | 0.74 (0.43, 1.27) | 0.97 (0.51, 1.82) |  |
| Overweight to obesity | 24 (3) | 0.66 (0.19, 2.24) | 1.16 (0.60, 2.26) | 1.03 (0.38, 2.76) |  | 0.67 (0.20, 2.28) | 1.18 (0.60, 2.29) | 1.06 (0.39, 2.84) |  |
| Overweight throughout | 143 (2.2) | 0.72 (0.40, 1.29) | 0.66 (0.40, 1.09) | 0.66 (0.37, 1.18) |  | 0.75 (0.42, 1.34) | 0.69 (0.42, 1.13) | 0.69 (0.38, 1.24) |  |
| Normal weight to overweight | 17 (1.5) | 0.86 (0.32, 2.32) | 0.50 (0.23, 1.10) | 0.39 (0.12, 1.32) |  | 0.92 (0.34, 2.46) | 0.52 (0.24, 1.16) | 0.41 (0.12, 1.39) |  |
| Normal weight throughout | 96 (1.5) | 0.63 (0.34, 1.19) | 0.60 (0.36, 1.01) | 0.50 (0.26, 0.97) |  | 0.67 (0.36, 1.27) | 0.64 (0.38, 1.08) | 0.53 (0.27, 1.04) |  |
| **Stroke mortality** |  |  |  |  | 0.195 |  |  |  | 0.475 |
| Obesity throughout | 15 (0.5) | 1.0 (Reference) | 0.33 (0.11, 1.00) | 0.31 (0.06, 1.54) |  | 1.0 (Reference) | 0.33 (0.11, 1.00) | 0.31 (0.06, 1.52) |  |
| Overweight to obesity | 0 (0.0) | - | - | - |  | - | - | - |  |
| Overweight throughout | 29 (0.4) | 0.53 (0.18, 1.59) | 0.40 (0.16, 1.02) | 0.35 (0.11, 1.16) |  | 0.54 (0.18, 1.61) | 0.41 (0.16, 1.04) | 0.35 (0.11, 1.17) |  |
| Normal weight to overweight | 4 (0.4) | 0.51 (0.06, 4.22) | 0.50 (0.13, 2.01) | - |  | 0.49 (0.06, 4.09) | 0.51 (0.13, 2.03) | - |  |
| Normal weight throughout | 26 (0.4) | 0.19 (0.04, 0.92) * | 0.57 (0.23, 1.42) | 0.35 (0.10, 1.25) |  | 0.19 (0.04, 0.96) * | 0.58 (0.23, 1.46) | 0.36 (0.10, 1.28) |  |
| **Heart failure** |  |  |  |  | 0.725 |  |  |  | 0.866 |
| Obesity throughout | 168 (5.1) | 1.0 (Reference) | 0.87 (0.58, 1.31) | 1.35 (0.86, 2.12) |  | 1.0 (Reference) | 0.86 (0.57, 1.29) | 1.29 (0.82, 2.02) |  |
| Overweight to obesity | 26 (3.3) | 0.83 (0.35, 1.99) | 0.58 (0.31, 1.06) | 0.64 (0.25, 1.65) |  | 0.82 (0.34, 1.96) | 0.56 (0.30, 1.03) | 0.62 (0.24, 1.60) |  |
| Overweight throughout | 172 (2.6) | 0.60 (0.38, 0.95) * | 0.39 (0.26, 0.58) * | 0.54 (0.34, 0.86) * |  | 0.60 (0.38, 0.96) * | 0.39 (0.26, 0.58) * | 0.54 (0.34, 0.85) * |  |
| Normal weight to overweight | 23 (2.1) | 0.51 (0.21, 1.22) | 0.42 (0.22, 0.79) | 0.31 (0.09, 1.00) |  | 0.51 (0.21, 1.22) | 0.41 (0.22, 0.78) * | 0.30 (0.09, 0.99) * |  |
| Normal weight throughout | 113 (1.8) | 0.55 (0.33, 0.91) * | 0.35 (0.23, 0.55) * | 0.42 (0.25, 0.71) * |  | 0.56 (0.34, 0.93) * | 0.36 (0.23, 0.55) * | 0.43 (0.25, 0.73) * |  |
| **Heart failure mortality** | |  |  |  | 0.563 |  |  |  | 0.671 |
| Obesity throughout | 29 (0.9) | 1.0 (Reference) | 1.42 (0.48, 4.17) | 1.20 (0.34, 4.29) |  | 1.0 (Reference) | 1.44 (0.49, 4.24) | 1.16 (0.32, 4.18) |  |
| Overweight to obesity | 2 (0.3) | - | 0.30 (0.03, 2.66) | 0.86 (0.09, 7.90) |  | - | 0.30 (0.03, 2.71) | 0.90 (0.10, 8.21) |  |
| Overweight throughout | 23 (0.3) | 0.80 (0.23, 2.73) | 0.42 (0.13, 1.30) | 0.42 (0.11, 1.70) |  | 0.82 (0.24, 2.81) | 0.43 (0.14, 1.35) | 0.44 (0.11, 1.77) |  |
| Normal weight to overweight | 4 (0.4) | 1.37 (0.25, 7.52) | 0.46 (0.08, 2.53) | - |  | 1.37 (0.25, 7.52) | 0.47 (0.09, 2.57) | - |  |
| Normal weight throughout | 17 (0.3) | 0.64 (0.16, 2.58) | 0.45 (0.14, 1.46) | 0.55 (0.14, 2.22) |  | 0.66 (0.16, 2.65) | 0.47 (0.14, 1.53) | 0.57 (0.14, 2.29) |  |
| **Atrial fibrillation** |  |  |  |  | 0.490 |  |  |  | 0.481 |
| Obesity throughout | 287 (9.0) | 1.0 (Reference) | 0.51 (0.40, 0.67) * | 0.36 (0.25, 0.53) * |  | 1.0 (Reference) | 0.50 (0.39, 0.65) * | 0.36 (0.25, 0.52) * |  |
| Overweight to obesity | 41 (5.4) | 0.85 (0.51, 1.40) | 0.27 (0.17, 0.44) * | 0.11 (0.03, 0.33) * |  | 0.83 (0.50, 1.37) | 0.27 (0.16, 0.43) * | 0.10 (0.03, 0.33) * |  |
| Overweight throughout | 398 (6.1) | 0.59 (0.45, 0.77) * | 0.32 (0.25, 0.41) * | 0.17 (0.12, 0.24) * |  | 0.58 (0.44, 0.76) * | 0.32 (0.25, 0.41) * | 0.17 (0.12, 0.24) * |  |
| Normal weight to overweight | 45 (4.1) | 0.52 (0.31, 0.86) | 0.20 (0.12, 0.33) * | 0.19 (0.09, 0.40) * |  | 0.52 (0.31, 0.86) * | 0.20 (0.12, 0.32) * | 0.18 (0.08, 0.39) * |  |
| Normal weight throughout | 257 (4.2) | 0.50 (0.37, 0.67) * | 0.29 (0.22, 0.37) * | 0.14 (0.09, 0.21) * |  | 0.49 (0.36, 0.66) * | 0.28 (0.21, 0.36) * | 0.14 (0.09, 0.21) * |  |
| **Atrial fibrillation mortality** | |  |  |  | 0.103 |  |  |  | 0.075 |
| Obesity throughout | 15 (0.5) | 1.0 (Reference) | 0.59 (0.19, 1.81) | 0.40 (0.08, 2.07) |  | 1.0 (Reference) | 0.60 (0.19, 1.85) | 0.41 (0.08, 2.15) |  |
| Overweight to obesity | 3 (0.4) | - | 0.68 (0.13, 3.56) | 0.90 (0.10, 7.98) |  | - | 0.62 (0.12, 3.28) | 0.85 (0.09, 7.63) |  |
| Overweight throughout | 13 (0.2) | 0.09 (0.01, 0.82) * | 0.28 (0.09, 0.86) * | 0.26 (0.06, 1.08) |  | 0.10 (0.01, 0.87) * | 0.28 (0.09, 0.86) * | 0.26 (0.06, 1.11) |  |
| Normal weight to overweight | 2 (0.2) | 1.22 (0.23, 6.35) | - | - |  | 1.12 (0.21, 5.90) | - | - |  |
| Normal weight throughout | 7 (0.1) | 0.28 (0.05, 1.50) | 0.23 (0.07, 0.81) | - |  | 0.28 (0.05, 1.49) | 0.22 (0.06, 0.79) * | - |  |

Note, BMI, body mass index; PRS, polygenic risk score; the PRSs presented are specifically used based on corresponding outcomes; Model 1 was adjusted for age, sex, race, Townsend Deprivation Index, annual household income, education attainment, 22 assessment centers, and the first 5 principal components of ancestry. Model 2 was further adjusted for family history of diabetes, family history of high blood pressure, and lifestyle factors including sleep duration, healthy diet, physical activity, smoking status, and alcohol intake frequency, based on Model 1; **^a^** likelihood tests was applied to test the significance of interaction term by comparing the model with and without the interaction term; **P* < 0.05.

**Table S13** **Combined effects of BMI-metabolic status (MH status: < 3 abnormal components) and PRSs on the risk of the specific cardiovascular outcomes**

| **Outcomes** | **Case (%)** | **Model 1** | | | | **Model 2** | | | |
| --- | --- | --- | --- | --- | --- | --- | --- | --- | --- |
|  |  | **High PRS** | **Moderate PRS** | **Low PRS** | ***P* for interaction ^a^** | **High PRS** | **Moderate PRS** | **Low PRS** | ***P* for interaction ^a^** |
| **Coronary disease** | |  |  |  | 0.005 |  |  |  | 0.006 |
| MUO | 8463 (14.4) | 1.0 (Reference) | 0.70 (0.67, 0.73) * | 0.46 (0.43, 0.50) * |  | 1.0 (Reference) | 0.70 (0.67, 0.73) * | 0.46 (0.43, 0.50) * |  |
| MUOW | 6434 (14.4) | 0.89 (0.84, 0.95) * | 0.58 (0.55, 0.61) * | 0.37 (0.34, 0.41) * |  | 0.91 (0.85, 0.96) * | 0.59 (0.56, 0.62) * | 0.38 (0.35, 0.41) * |  |
| MUN | 1347 (14.5) | 0.81 (0.73, 0.90) * | 0.55 (0.51, 0.60) * | 0.35 (0.30, 0.42) * |  | 0.84 (0.75, 0.93) * | 0.56 (0.51, 0.60) * | 0.36 (0.30, 0.42) * |  |
| MHO | 3122 (8.4) | 0.73 (0.67, 0.79) * | 0.49 (0.46, 0.52) * | 0.34 (0.31, 0.38) * |  | 0.75 (0.70, 0.82) * | 0.50 (0.47, 0.53) * | 0.35 (0.32, 0.39) * |  |
| MHOW | 9073 (7.1) | 0.60 (0.57, 0.64) * | 0.37 (0.35, 0.39) * | 0.24 (0.22, 0.25) * |  | 0.64 (0.60, 0.67) * | 0.39 (0.37, 0.41) * | 0.25 (0.23, 0.27) * |  |
| MHN | 6058 (4.9) | 0.48 (0.45, 0.51) * | 0.29 (0.28, 0.31) * | 0.20 (0.18, 0.21) * |  | 0.51 (0.48, 0.54) * | 0.31 (0.29, 0.33) * | 0.21 (0.20, 0.23) * |  |
| **Coronary disease mortality** |  |  |  |  | 0.722 |  |  |  | 0.791 |
| MUO | 1239 (2.1) | 1.0 (Reference) | 0.64 (0.57, 0.73) * | 0.44 (0.36, 0.53) * |  | 1.0 (Reference) | 0.64 (0.56, 0.72) * | 0.43 (0.36, 0.52) * |  |
| MUOW | 878 (2) | 0.78 (0.66, 0.91) * | 0.50 (0.44, 0.57) * | 0.28 (0.22, 0.35) * |  | 0.78 (0.67, 0.91) * | 0.50 (0.44, 0.57) * | 0.28 (0.22, 0.35) * |  |
| MUN | 208 (2.2) | 0.75 (0.57, 0.98) * | 0.51 (0.41, 0.63) * | 0.42 (0.28, 0.62) * |  | 0.76 (0.58, 0.99) * | 0.50 (0.40, 0.61) * | 0.40 (0.27, 0.60) * |  |
| MHO | 321 (0.9) | 0.57 (0.45, 0.72) * | 0.39 (0.33, 0.47) * | 0.27 (0.20, 0.36) * |  | 0.60 (0.47, 0.76) * | 0.41 (0.34, 0.48) * | 0.28 (0.21, 0.38) * |  |
| MHOW | 964 (0.8) | 0.49 (0.42, 0.58) * | 0.31 (0.27, 0.35) * | 0.17 (0.14, 0.21) * |  | 0.52 (0.45, 0.61) * | 0.32 (0.28, 0.37) * | 0.18 (0.15, 0.22) * |  |
| MHN | 667 (0.5) | 0.42 (0.35, 0.51) * | 0.28 (0.24, 0.32) * | 0.18 (0.14, 0.22) * |  | 0.44 (0.37, 0.53) * | 0.29 (0.25, 0.34) * | 0.19 (0.15, 0.23) * |  |
| **Myocardial infarction** |  |  |  |  | 0.355 |  |  |  | 0.290 |
| MUO | 4603 (7.3) | 1.0 (Reference) | 0.75 (0.70, 0.80) * | 0.56 (0.51, 0.62) * |  | 1.0 (Reference) | 0.75 (0.70, 0.81) * | 0.56 (0.51, 0.62) * |  |
| MUOW | 3734 (7.8) | 0.93 (0.85, 1.01) | 0.66 (0.62, 0.71) * | 0.47 (0.42, 0.52) * |  | 0.95 (0.87, 1.03) | 0.68 (0.63, 0.73) * | 0.47 (0.43, 0.53) * |  |
| MUN | 785 (7.7) | 0.83 (0.72, 0.96) * | 0.60 (0.54, 0.67) * | 0.50 (0.41, 0.61) * |  | 0.85 (0.74, 0.99) * | 0.61 (0.54, 0.68) * | 0.50 (0.42, 0.61) * |  |
| MHO | 1243 (3.3) | 0.58 (0.51, 0.66) * | 0.42 (0.38, 0.46) * | 0.37 (0.32, 0.43) * |  | 0.61 (0.54, 0.69) * | 0.44 (0.40, 0.48) * | 0.39 (0.34, 0.45) * |  |
| MHOW | 3922 (3.0) | 0.51 (0.47, 0.56) * | 0.36 (0.33, 0.38) * | 0.26 (0.24, 0.29) * |  | 0.55 (0.50, 0.60) * | 0.38 (0.36, 0.41) * | 0.28 (0.25, 0.31) * |  |
| MHN | 2596 (2.1) | 0.41 (0.38, 0.45) * | 0.30 (0.28, 0.32) * | 0.22 (0.20, 0.25) * |  | 0.44 (0.40, 0.49) * | 0.32 (0.30, 0.35) * | 0.24 (0.21, 0.26) * |  |
| **Myocardial infarction mortality** | |  |  |  | 0.382 |  |  |  | 0.366 |
| MUO | 497 (0.8) | 1.0 (Reference) | 0.79 (0.64, 0.98) * | 0.59 (0.44, 0.78) * |  | 1.0 (Reference) | 0.79 (0.64, 0.98) * | 0.59 (0.45, 0.79) * |  |
| MUOW | 365 (0.8) | 0.61 (0.46, 0.81) * | 0.69 (0.56, 0.86) * | 0.38 (0.27, 0.54) * |  | 0.62 (0.47, 0.83) * | 0.71 (0.57, 0.88) * | 0.39 (0.27, 0.55) * |  |
| MUN | 83 (0.8) | 0.75 (0.48, 1.19) | 0.60 (0.43, 0.84) * | 0.48 (0.27, 0.87) * |  | 0.78 (0.49, 1.23) | 0.61 (0.44, 0.85) * | 0.48 (0.26, 0.86) * |  |
| MHO | 129 (0.3) | 0.77 (0.54, 1.11) | 0.47 (0.36, 0.63) * | 0.28 (0.17, 0.47) * |  | 0.82 (0.57, 1.18) | 0.50 (0.38, 0.67) * | 0.30 (0.18, 0.50) * |  |
| MHOW | 361 (0.3) | 0.46 (0.35, 0.60) * | 0.36 (0.29, 0.45) * | 0.26 (0.19, 0.35) * |  | 0.49 (0.37, 0.65) * | 0.39 (0.31, 0.49) * | 0.27 (0.20, 0.38) * |  |
| MHN | 258 (0.2) | 0.39 (0.28, 0.53) * | 0.35 (0.28, 0.45) * | 0.23 (0.16, 0.34) * |  | 0.42 (0.31, 0.57) * | 0.38 (0.30, 0.48) * | 0.25 (0.17, 0.36) * |  |
| **Stroke** |  |  |  |  | 0.378 |  |  |  | 0.395 |
| MUO | 2369 (3.7) | 1.0 (Reference) | 0.98 (0.88, 1.08) | 0.81 (0.71, 0.93) * |  | 1.0 (Reference) | 0.98 (0.88, 1.08) | 0.81 (0.71, 0.93) * |  |
| MUOW | 1954 (4) | 0.94 (0.82, 1.07) | 0.85 (0.77, 0.95) * | 0.83 (0.73, 0.95) * |  | 0.95 (0.83, 1.08) | 0.86 (0.77, 0.95) * | 0.84 (0.73, 0.96) * |  |
| MUN | 465 (4.4) | 1.01 (0.82, 1.25) | 0.91 (0.79, 1.06) | 0.82 (0.64, 1.04) |  | 1.01 (0.82, 1.25) | 0.92 (0.79, 1.06) | 0.83 (0.65, 1.05) |  |
| MHO | 828 (2.2) | 0.78 (0.66, 0.92) * | 0.69 (0.61, 0.78) * | 0.61 (0.51, 0.73) * |  | 0.79 (0.67, 0.94) * | 0.71 (0.62, 0.80) * | 0.62 (0.52, 0.75) * |  |
| MHOW | 2788 (2.2) | 0.71 (0.63, 0.80) * | 0.63 (0.57, 0.70) * | 0.65 (0.57, 0.73) * |  | 0.73 (0.65, 0.83) * | 0.65 (0.59, 0.72) * | 0.67 (0.59, 0.75) * |  |
| MHN | 2365 (1.9) | 0.72 (0.64, 0.82) * | 0.65 (0.58, 0.72) * | 0.59 (0.52, 0.67) * |  | 0.75 (0.66, 0.85) * | 0.67 (0.60, 0.74) * | 0.61 (0.53, 0.69) * |  |
| **Stroke mortality** |  |  |  |  | 0.442 |  |  |  | 0.439 |
| MUO | 497 (0.8) | 1.0 (Reference) | 0.93 (0.74, 1.15) | 0.68 (0.51, 0.91) |  | 1.0 (Reference) | 0.92 (0.74, 1.15) | 0.68 (0.51, 0.91) |  |
| MUOW | 433 (0.9) | 0.95 (0.72, 1.25) | 0.83 (0.67, 1.04) | 0.77 (0.58, 1.03) |  | 0.96 (0.73, 1.26) | 0.84 (0.67, 1.05) | 0.78 (0.58, 1.04) |  |
| MUN | 141 (1.3) | 1.29 (0.87, 1.92) | 1.27 (0.96, 1.68) | 0.91 (0.57, 1.47) |  | 1.28 (0.86, 1.90) | 1.27 (0.96, 1.69) | 0.93 (0.58, 1.50) |  |
| MHO | 158 (0.4) | 0.57 (0.38, 0.86) * | 0.63 (0.48, 0.83) * | 0.59 (0.40, 0.88) * |  | 0.59 (0.39, 0.89) * | 0.65 (0.49, 0.86) * | 0.61 (0.41, 0.91) * |  |
| MHOW | 504 (0.4) | 0.71 (0.55, 0.92) * | 0.51 (0.41, 0.64) * | 0.51 (0.38, 0.67) * |  | 0.75 (0.58, 0.97) * | 0.54 (0.43, 0.67) * | 0.53 (0.40, 0.70) * |  |
| MHN | 503 (0.4) | 0.65 (0.49, 0.85) * | 0.66 (0.53, 0.82) * | 0.60 (0.45, 0.79) * |  | 0.69 (0.52, 0.90) * | 0.69 (0.56, 0.87) * | 0.63 (0.48, 0.83) * |  |
| **Heart failure** |  |  |  |  | 0.417 |  |  |  | 0.427 |
| MUO | 4770 (7.5) | 1.0 (Reference) | 0.85 (0.79, 0.91) * | 0.76 (0.69, 0.83) * |  | 1.0 (Reference) | 0.84 (0.79, 0.90) * | 0.75 (0.69, 0.82) * |  |
| MUOW | 2841 (5.7) | 0.67 (0.61, 0.74) * | 0.51 (0.47, 0.55) * | 0.47 (0.42, 0.52) * |  | 0.69 (0.62, 0.75) * | 0.52 (0.48, 0.56) * | 0.48 (0.43, 0.53) * |  |
| MUN | 607 (5.7) | 0.54 (0.45, 0.65) * | 0.51 (0.45, 0.57) * | 0.42 (0.35, 0.52) * |  | 0.55 (0.46, 0.66) * | 0.51 (0.45, 0.57) * | 0.43 (0.35, 0.53) * |  |
| MHO | 1458 (3.8) | 0.65 (0.57, 0.73) * | 0.56 (0.52, 0.62) * | 0.53 (0.47, 0.60) * |  | 0.67 (0.59, 0.76) * | 0.59 (0.54, 0.64) * | 0.55 (0.48, 0.63) * |  |
| MHOW | 3012 (2.3) | 0.36 (0.33, 0.40) * | 0.33 (0.30, 0.35) * | 0.27 (0.25, 0.30) * |  | 0.39 (0.35, 0.43) * | 0.34 (0.32, 0.37) * | 0.29 (0.26, 0.32) * |  |
| MHN | 2130 (1.7) | 0.32 (0.29, 0.36) * | 0.27 (0.25, 0.29) * | 0.25 (0.22, 0.28) * |  | 0.34 (0.31, 0.38) * | 0.29 (0.27, 0.31) * | 0.26 (0.23, 0.29) * |  |
| **Heart failure mortality** |  |  |  |  | 0.654 |  |  |  | 0.655 |
| MUO | 773 (1.2) | 1.0 (Reference) | 0.86 (0.72, 1.02) | 0.74 (0.59, 0.92) * |  | 1.0 (Reference) | 0.86 (0.72, 1.02) | 0.74 (0.59, 0.92) * |  |
| MUOW | 417 (0.8) | 0.62 (0.49, 0.79) * | 0.47 (0.39, 0.57) * | 0.39 (0.30, 0.52) * |  | 0.64 (0.50, 0.81) * | 0.48 (0.39, 0.58) * | 0.39 (0.30, 0.52) * |  |
| MUN | 110 (1.0) | 0.71 (0.48, 1.06) | 0.50 (0.37, 0.67) * | 0.55 (0.36, 0.86) * |  | 0.72 (0.48, 1.08) | 0.50 (0.37, 0.67) * | 0.56 (0.36, 0.87) * |  |
| MHO | 179 (0.5) | 0.49 (0.34, 0.70) * | 0.47 (0.37, 0.60) * | 0.50 (0.35, 0.70) * |  | 0.52 (0.36, 0.75) * | 0.50 (0.39, 0.64) * | 0.53 (0.37, 0.75) * |  |
| MHOW | 335 (0.3) | 0.28 (0.22, 0.37) * | 0.25 (0.21, 0.31) * | 0.18 (0.14, 0.25) * |  | 0.31 (0.24, 0.41) * | 0.28 (0.23, 0.34) * | 0.20 (0.15, 0.27) * |  |
| MHN | 282 (0.2) | 0.32 (0.24, 0.43) * | 0.24 (0.20, 0.30) * | 0.25 (0.19, 0.34) * |  | 0.35 (0.26, 0.46) * | 0.26 (0.21, 0.33) * | 0.27 (0.20, 0.36) * |  |
| **Atrial fibrillation** |  |  |  |  | < 0.001 |  |  |  | < 0.001 |
| MUO | 7413 (11.8) | 1.0 (Reference) | 0.56 (0.53, 0.59) * | 0.33 (0.30, 0.35) * |  | 1.0 (Reference) | 0.56 (0.53, 0.59) * | 0.33 (0.30, 0.35) * |  |
| MUOW | 4925 (10.1) | 0.73 (0.68, 0.77) * | 0.37 (0.35, 0.39) * | 0.22 (0.20, 0.24) * |  | 0.73 (0.68, 0.78) * | 0.37 (0.35, 0.39) * | 0.22 (0.20, 0.24) * |  |
| MUN | 962 (9.2) | 0.55 (0.49, 0.62) * | 0.34 (0.31, 0.38) * | 0.15 (0.12, 0.18) * |  | 0.55 (0.49, 0.62) * | 0.35 (0.32, 0.38) * | 0.15 (0.12, 0.18) * |  |
| MHO | 3031 (8.0) | 0.98 (0.91, 1.05) | 0.49 (0.46, 0.52) * | 0.26 (0.23, 0.30) * |  | 0.99 (0.92, 1.06) | 0.49 (0.46, 0.53) * | 0.27 (0.24, 0.30) * |  |
| MHOW | 7381 (5.7) | 0.61 (0.57, 0.64) * | 0.30 (0.29, 0.32) * | 0.17 (0.15, 0.18) * |  | 0.62 (0.58, 0.65) * | 0.31 (0.29, 0.32) * | 0.17 (0.16, 0.19) * |  |
| MHN | 5580 (4.5) | 0.55 (0.52, 0.58) * | 0.28 (0.26, 0.29) * | 0.15 (0.13, 0.16) * |  | 0.56 (0.53, 0.59) * | 0.28 (0.27, 0.30) * | 0.15 (0.14, 0.16) * |  |
| **Atrial fibrillation mortality** |  |  |  |  | 0.123 |  |  |  | 0.112 |
| MUO | 386 (0.6) | 1.0 (Reference) | 0.57 (0.46, 0.71) * | 0.31 (0.22, 0.45) * |  | 1.0 (Reference) | 0.57 (0.46, 0.71) * | 0.31 (0.22, 0.45) * |  |
| MUOW | 232 (0.5) | 0.65 (0.49, 0.86) * | 0.32 (0.25, 0.41) * | 0.20 (0.13, 0.31) * |  | 0.66 (0.50, 0.87) * | 0.32 (0.25, 0.41) * | 0.21 (0.14, 0.31) * |  |
| MUN | 56 (0.5) | 0.59 (0.35, 0.98) * | 0.40 (0.27, 0.58) * | 0.10 (0.03, 0.31) * |  | 0.59 (0.36, 0.98) * | 0.40 (0.28, 0.59) * | 0.10 (0.03, 0.31) * |  |
| MHO | 130 (0.3) | 0.87 (0.62, 1.22) | 0.42 (0.32, 0.57) * | 0.34 (0.20, 0.56) * |  | 0.90 (0.64, 1.26) | 0.44 (0.33, 0.59) * | 0.35 (0.21, 0.58) * |  |
| MHOW | 225 (0.2) | 0.37 (0.28, 0.49) * | 0.20 (0.16, 0.26) * | 0.13 (0.09, 0.20) * |  | 0.39 (0.29, 0.52) * | 0.21 (0.17, 0.28) * | 0.14 (0.09, 0.21) * |  |
| MHN | 197 (0.2) | 0.46 (0.34, 0.61) * | 0.24 (0.18, 0.31) * | 0.05 (0.03, 0.11) * |  | 0.48 (0.36, 0.64) * | 0.25 (0.19, 0.32) * | 0.06 (0.03, 0.11) * |  |

Note, BMI, body mass index; MH status was defined as < 3 abnormal components; MH, metabolically healthy; MHN, metabolically healthy normal weight; MHOW, metabolically healthy overweight; MHO, metabolically healthy obesity; MUN, metabolically unhealthy normal weight; MUOW, metabolically unhealthy overweight; MUO, metabolically unhealthy obesity; PRS, polygenic risk score; the PRSs presented are specifically used based on corresponding outcomes; Model 1 was adjusted for age, sex, race, Townsend Deprivation Index, annual household income, education attainment, 22 assessment centers, and the first 5 principal components of ancestry. Model 2 was further adjusted for family history of diabetes, family history of high blood pressure, and lifestyle factors including sleep duration, healthy diet, physical activity, smoking status, and alcohol intake frequency, based on Model 1; **^a^** likelihood tests was applied to test the significance of interaction term by comparing the model with and without the interaction term; **P* < 0.05.

**Table S14 Combined effects of BMI-metabolic status (MH status: < 2 abnormal components) and PRSs on the risk of cardiovascular outcomes and all-cause mortality**

| **Outcomes** | **Case (%)** | **Model 1** | | | | **Model 2** | | | |
| --- | --- | --- | --- | --- | --- | --- | --- | --- | --- |
|  |  | **High PRS** | **Moderate PRS** | **Low PRS** | ***P* for interaction ^a^** | **High PRS** | **Moderate PRS** | **Low PRS** | ***P* for interaction ^a^** |
| **All-cause mortality** |  |  |  |  | 0.015 |  |  |  | 0.007 |
| MUO | 7858 (9.8) | 1.0 (Reference) | 0.97 (0.91, 1.02) | 0.86 (0.80, 0.92) * |  | 1.0 (Reference) | 0.96 (0.91, 1.02) | 0.85 (0.79, 0.91) * |  |
| MUOW | 7897 (8.8) | 0.81 (0.76, 0.87) * | 0.78 (0.73, 0.82) * | 0.72 (0.67, 0.78) * |  | 0.82 (0.76, 0.87) * | 0.78 (0.73, 0.82) * | 0.73 (0.68, 0.78) * |  |
| MUN | 2749 (9.4) | 0.91 (0.83, 1.00) | 0.82 (0.77, 0.88) * | 0.88 (0.80, 0.97) * |  | 0.90 (0.82, 0.99) * | 0.82 (0.76, 0.87) * | 0.88 (0.80, 0.97) * |  |
| MHO | 561 (5.4) | 0.72 (0.59, 0.88) * | 0.71 (0.63, 0.80) * | 0.81 (0.68, 0.96) * |  | 0.74 (0.61, 0.91) * | 0.72 (0.64, 0.82) * | 0.83 (0.70, 1.00) |  |
| MHOW | 3907 (5.3) | 0.69 (0.63, 0.75) * | 0.63 (0.59, 0.67) * | 0.56 (0.51, 0.61) * |  | 0.72 (0.66, 0.79) * | 0.65 (0.61, 0.70) * | 0.58 (0.53, 0.63) * |  |
| MHN | 4944 (5) | 0.74 (0.68, 0.80) * | 0.67 (0.63, 0.71) * | 0.67 (0.62, 0.72) * |  | 0.76 (0.70, 0.83) * | 0.69 (0.65, 0.73) * | 0.69 (0.64, 0.75) * |  |
| **Cardiovascular events** | |  |  |  | 0.003 |  |  |  | 0.003 |
| MUO | 17 819 (21.3) | 1.0 (Reference) | 0.77 (0.75, 0.80) * | 0.62 (0.59, 0.65) * |  | 1.0 (Reference) | 0.78 (0.75, 0.80) * | 0.62 (0.59, 0.65) * |  |
| MUOW | 17 244 (18.5) | 0.79 (0.76, 0.83) * | 0.57 (0.55, 0.59) * | 0.44 (0.42, 0.46) * |  | 0.81 (0.77, 0.84) * | 0.58 (0.56, 0.60) * | 0.44 (0.42, 0.47) * |  |
| MUN | 4970 (16.2) | 0.71 (0.67, 0.76) * | 0.50 (0.47, 0.52) * | 0.41 (0.38, 0.44) * |  | 0.72 (0.68, 0.77) * | 0.51 (0.48, 0.53) * | 0.41 (0.38, 0.45) * |  |
| MHO | 1271 (11.9) | 0.71 (0.63, 0.80) * | 0.54 (0.50, 0.59) * | 0.49 (0.43, 0.55) * |  | 0.72 (0.64, 0.82) * | 0.56 (0.52, 0.60) * | 0.50 (0.44, 0.57) * |  |
| MHOW | 8582 (11.2) | 0.58 (0.55, 0.62) * | 0.45 (0.43, 0.47) * | 0.35 (0.33, 0.37) * |  | 0.61 (0.57, 0.64) * | 0.47 (0.45, 0.48) * | 0.36 (0.34, 0.38) * |  |
| MHN | 8931 (8.8) | 0.51 (0.49, 0.54) * | 0.39 (0.37, 0.41) * | 0.32 (0.30, 0.34) * |  | 0.53 (0.51, 0.56) * | 0.41 (0.39, 0.42) * | 0.34 (0.32, 0.35) * |  |
| **Cardiovascular mortality** | |  |  |  | 0.443 |  |  |  | 0.467 |
| MUO | 2376 (2.8) | 1.0 (Reference) | 0.72 (0.66, 0.79) * | 0.57 (0.50, 0.65) * |  | 1.0 (Reference) | 0.72 (0.65, 0.79) * | 0.57 (0.50, 0.65) * |  |
| MUOW | 2121 (2.3) | 0.70 (0.62, 0.78) * | 0.51 (0.46, 0.56) * | 0.39 (0.34, 0.44) * |  | 0.71 (0.63, 0.79) * | 0.51 (0.47, 0.57) * | 0.39 (0.34, 0.45) * |  |
| MUN | 734 (2.4) | 0.80 (0.68, 0.94) * | 0.54 (0.47, 0.61) * | 0.48 (0.40, 0.59) * |  | 0.79 (0.67, 0.93) * | 0.54 (0.47, 0.61) * | 0.48 (0.40, 0.59) * |  |
| MHO | 116 (1.1) | 0.57 (0.39, 0.84) * | 0.40 (0.31, 0.52) * | 0.44 (0.29, 0.66) * |  | 0.59 (0.40, 0.87) * | 0.41 (0.32, 0.54) * | 0.46 (0.31, 0.69) * |  |
| MHOW | 844 (1.1) | 0.54 (0.46, 0.63) * | 0.36 (0.32, 0.41) * | 0.26 (0.22, 0.32) * |  | 0.57 (0.49, 0.67) * | 0.38 (0.34, 0.43) * | 0.28 (0.23, 0.34) * |  |
| MHN | 931 (0.9) | 0.57 (0.49, 0.66) * | 0.34 (0.31, 0.39) * | 0.29 (0.24, 0.34) * |  | 0.60 (0.52, 0.70) * | 0.36 (0.32, 0.41) * | 0.31 (0.26, 0.36) * |  |
| **Coronary disease** |  |  |  |  | 0.005 |  |  |  | 0.004 |
| MUO | 10 921 (12.8) | 1.0 (Reference) | 0.69 (0.66, 0.72) * | 0.46 (0.43, 0.49) * |  | 1.0 (Reference) | 0.69 (0.66, 0.72) * | 0.46 (0.43, 0.49) * |  |
| MUOW | 10 920 (11.5) | 0.85 (0.81, 0.90) * | 0.53 (0.51, 0.56) * | 0.33 (0.31, 0.35) * |  | 0.87 (0.83, 0.92) * | 0.55 (0.52, 0.57) * | 0.34 (0.32, 0.36) * |  |
| MUN | 3,092 (9.9) | 0.76 (0.70, 0.81) * | 0.47 (0.44, 0.50) * | 0.29 (0.26, 0.32) * |  | 0.78 (0.72, 0.84) * | 0.48 (0.45, 0.51) * | 0.30 (0.27, 0.33) * |  |
| MHO | 664 (6.2) | 0.63 (0.54, 0.74) * | 0.42 (0.38, 0.47) * | 0.32 (0.26, 0.38) * |  | 0.66 (0.56, 0.77) * | 0.44 (0.39, 0.48) * | 0.33 (0.27, 0.40) * |  |
| MHOW | 4587 (5.9) | 0.56 (0.52, 0.60) * | 0.35 (0.34, 0.37) * | 0.24 (0.22, 0.26) * |  | 0.59 (0.55, 0.63) * | 0.37 (0.36, 0.39) * | 0.25 (0.23, 0.27) * |  |
| MHN | 4313 (4.2) | 0.46 (0.43, 0.49) * | 0.28 (0.27, 0.30) * | 0.20 (0.19, 0.22) * |  | 0.48 (0.45, 0.52) * | 0.30 (0.28, 0.32) * | 0.22 (0.20, 0.23) * |  |
| **Coronary disease mortality** | |  |  |  | 0.493 |  |  |  | 0.538 |
| MUO | 1502 (1.8) | 1.0 (Reference) | 0.64 (0.57, 0.72) * | 0.44 (0.37, 0.52) * |  | 1.0 (Reference) | 0.64 (0.57, 0.71) * | 0.44 (0.37, 0.52) * |  |
| MUOW | 1392 (1.5) | 0.74 (0.65, 0.85) * | 0.48 (0.43, 0.54) * | 0.25 (0.21, 0.31) * |  | 0.75 (0.66, 0.86) * | 0.48 (0.43, 0.54) * | 0.26 (0.21, 0.31) * |  |
| MUN | 439 (1.4) | 0.74 (0.61, 0.90) * | 0.49 (0.42, 0.57) * | 0.30 (0.22, 0.41) * |  | 0.74 (0.61, 0.90) * | 0.48 (0.41, 0.56) * | 0.30 (0.22, 0.40) * |  |
| MHO | 58 (0.5) | 0.46 (0.27, 0.78) * | 0.35 (0.25, 0.49) * | 0.15 (0.07, 0.33) * |  | 0.48 (0.28, 0.81) * | 0.37 (0.26, 0.51) * | 0.16 (0.07, 0.35) * |  |
| MHOW | 450 (0.6) | 0.49 (0.40, 0.60) * | 0.28 (0.24, 0.32) * | 0.17 (0.13, 0.23) * |  | 0.52 (0.43, 0.64) * | 0.29 (0.25, 0.34) * | 0.18 (0.14, 0.24) * |  |
| MHN | 436 (0.4) | 0.39 (0.32, 0.49) * | 0.26 (0.22, 0.31) * | 0.19 (0.15, 0.24) * |  | 0.41 (0.33, 0.51) * | 0.27 (0.24, 0.32) * | 0.20 (0.15, 0.26) * |  |
| **Myocardial infarction** |  |  |  |  | 0.527 |  |  |  | 0.409 |
| MUO | 5587 (6.2) | 1.0 (Reference) | 0.75 (0.70, 0.80) * | 0.58 (0.53, 0.63) * |  | 1.0 (Reference) | 0.75 (0.70, 0.80) * | 0.58 (0.53, 0.63) * |  |
| MUOW | 5788 (5.8) | 0.88 (0.82, 0.95) * | 0.61 (0.58, 0.65) * | 0.43 (0.39, 0.47) * |  | 0.90 (0.84, 0.97) * | 0.63 (0.59, 0.67) * | 0.44 (0.40, 0.48) * |  |
| MUN | 1607 (4.9) | 0.75 (0.68, 0.84) * | 0.55 (0.50, 0.59) * | 0.41 (0.36, 0.48) * |  | 0.78 (0.70, 0.86) * | 0.56 (0.51, 0.61) * | 0.42 (0.37, 0.49) * |  |
| MHO | 259 (2.4) | 0.59 (0.46, 0.76) * | 0.37 (0.31, 0.44) * | 0.33 (0.25, 0.44) * |  | 0.63 (0.49, 0.80) * | 0.39 (0.33, 0.46) * | 0.34 (0.26, 0.46) * |  |
| MHOW | 1868 (2.4) | 0.48 (0.43, 0.53) * | 0.34 (0.32, 0.37) * | 0.26 (0.23, 0.29) * |  | 0.51 (0.46, 0.57) * | 0.37 (0.34, 0.40) * | 0.27 (0.24, 0.31) * |  |
| MHN | 1774 (1.7) | 0.41 (0.37, 0.46) * | 0.29 (0.27, 0.32) * | 0.22 (0.19, 0.25) * |  | 0.44 (0.40, 0.49) * | 0.32 (0.29, 0.34) * | 0.24 (0.21, 0.27) * |  |
| **Myocardial infarction mortality** | |  |  |  | 0.038 |  |  |  | 0.033 |
| MUO | 603 (0.7) | 1.0 (Reference) | 0.78 (0.64, 0.94) * | 0.55 (0.42, 0.72) * |  | 1.0 (Reference) | 0.78 (0.64, 0.94) * | 0.56 (0.43, 0.72) * |  |
| MUOW | 571 (0.6) | 0.64 (0.51, 0.81) * | 0.64 (0.53, 0.77) * | 0.32 (0.24, 0.43) * |  | 0.66 (0.52, 0.83) * | 0.66 (0.55, 0.80) * | 0.33 (0.24, 0.44) * |  |
| MUN | 171 (0.5) | 0.70 (0.50, 0.98) * | 0.59 (0.46, 0.76) * | 0.35 (0.22, 0.56) * |  | 0.71 (0.51, 1.00) | 0.60 (0.47, 0.77) * | 0.36 (0.23, 0.57) * |  |
| MHO | 23 (0.2) | 0.93 (0.49, 1.77) | 0.28 (0.15, 0.52) * | 0.22 (0.07, 0.69) * |  | 0.99 (0.52, 1.88) | 0.29 (0.15, 0.55) * | 0.23 (0.07, 0.73) * |  |
| MHOW | 155 (0.2) | 0.37 (0.25, 0.53) * | 0.28 (0.21, 0.36) * | 0.31 (0.21, 0.45) * |  | 0.40 (0.28, 0.58) * | 0.30 (0.23, 0.39) * | 0.33 (0.23, 0.48) * |  |
| MHN | 170 (0.2) | 0.35 (0.25, 0.50) * | 0.32 (0.25, 0.41) * | 0.24 (0.16, 0.37) * |  | 0.38 (0.27, 0.55) * | 0.35 (0.27, 0.44) * | 0.26 (0.17, 0.40) * |  |
| **Stroke** |  |  |  |  | 0.418 |  |  |  | 0.423 |
| MUO | 3009 (3.3) | 1.0 (Reference) | 0.96 (0.87, 1.05) | 0.81 (0.72, 0.91) * |  | 1.0 (Reference) | 0.96 (0.88, 1.05) | 0.81 (0.72, 0.91) * |  |
| MUOW | 3258 (3.2) | 0.91 (0.82, 1.01) | 0.81 (0.74, 0.88) * | 0.80 (0.72, 0.90) * |  | 0.92 (0.83, 1.03) | 0.82 (0.75, 0.89) * | 0.81 (0.73, 0.91) * |  |
| MUN | 1040 (3.2) | 0.92 (0.80, 1.07) | 0.84 (0.75, 0.94) * | 0.72 (0.61, 0.85) * |  | 0.93 (0.80, 1.08) | 0.85 (0.76, 0.95) * | 0.73 (0.62, 0.86) * |  |
| MHO | 188 (1.7) | 0.74 (0.54, 1.01) | 0.67 (0.54, 0.81) * | 0.56 (0.39, 0.79) * |  | 0.76 (0.55, 1.03) | 0.69 (0.56, 0.84) * | 0.57 (0.40, 0.81) * |  |
| MHOW | 1484 (1.9) | 0.70 (0.61, 0.80) * | 0.63 (0.57, 0.70) * | 0.65 (0.57, 0.75) * |  | 0.73 (0.63, 0.83) * | 0.66 (0.59, 0.73) * | 0.68 (0.59, 0.78) * |  |
| MHN | 1790 (1.7) | 0.75 (0.66, 0.85) * | 0.66 (0.60, 0.73) * | 0.62 (0.54, 0.70) * |  | 0.78 (0.68, 0.88) * | 0.69 (0.62, 0.76) * | 0.64 (0.56, 0.73) * |  |
| **Stroke mortality** |  |  |  |  | 0.339 |  |  |  | 0.338 |
| MUO | 613 (0.7) | 1.0 (Reference) | 0.95 (0.78, 1.16) | 0.74 (0.57, 0.96) * |  | 1.0 (Reference) | 0.96 (0.78, 1.17) | 0.74 (0.57, 0.96) * |  |
| MUOW | 653 (0.6) | 0.90 (0.71, 1.14) | 0.77 (0.63, 0.94) * | 0.76 (0.59, 0.97) * |  | 0.92 (0.72, 1.17) | 0.79 (0.64, 0.96) * | 0.77 (0.61, 0.99) * |  |
| MUN | 265 (0.8) | 1.11 (0.82, 1.51) | 1.05 (0.83, 1.32) | 0.76 (0.54, 1.09) |  | 1.13 (0.83, 1.53) | 1.06 (0.84, 1.34) | 0.78 (0.55, 1.11) |  |
| MHO | 42 (0.4) | 0.74 (0.36, 1.51) | 0.81 (0.53, 1.23) | 0.71 (0.35, 1.46) |  | 0.77 (0.38, 1.58) | 0.84 (0.55, 1.29) | 0.75 (0.37, 1.52) |  |
| MHOW | 284 (0.4) | 0.89 (0.67, 1.18) | 0.59 (0.47, 0.75) * | 0.54 (0.39, 0.75) * |  | 0.95 (0.72, 1.26) | 0.63 (0.50, 0.79) * | 0.57 (0.41, 0.79) * |  |
| MHN | 379 (0.4) | 0.70 (0.52, 0.94) * | 0.74 (0.60, 0.92) * | 0.71 (0.53, 0.94) * |  | 0.74 (0.56, 1.00) | 0.79 (0.63, 0.98) * | 0.75 (0.56, 1.00) |  |
| **Heart failure** |  |  |  |  | 0.608 |  |  |  | 0.582 |
| MUO | 5950 (6.5) | 1.0 (Reference) | 0.84 (0.79, 0.90) * | 0.76 (0.70, 0.82) * |  | 1.0 (Reference) | 0.84 (0.79, 0.90) * | 0.75 (0.70, 0.82) * |  |
| MUOW | 4289 (4.3) | 0.59 (0.54, 0.64) * | 0.47 (0.44, 0.50) * | 0.42 (0.38, 0.46) * |  | 0.60 (0.55, 0.65) * | 0.48 (0.45, 0.51) * | 0.43 (0.39, 0.47) * |  |
| MUN | 1203 (3.6) | 0.49 (0.43, 0.56) * | 0.42 (0.38, 0.46) * | 0.39 (0.34, 0.45) * |  | 0.50 (0.44, 0.57) * | 0.43 (0.39, 0.47) * | 0.40 (0.35, 0.46) * |  |
| MHO | 278 (2.5) | 0.44 (0.33, 0.58) * | 0.50 (0.43, 0.59) * | 0.49 (0.37, 0.63) * |  | 0.46 (0.34, 0.61) * | 0.52 (0.44, 0.61) * | 0.51 (0.39, 0.67) * |  |
| MHOW | 1564 (2) | 0.38 (0.34, 0.43) * | 0.33 (0.30, 0.36) * | 0.28 (0.24, 0.31) * |  | 0.40 (0.36, 0.45) * | 0.35 (0.32, 0.38) * | 0.29 (0.26, 0.33) * |  |
| MHN | 1534 (1.5) | 0.33 (0.29, 0.37) * | 0.28 (0.26, 0.30) * | 0.24 (0.21, 0.28) * |  | 0.35 (0.31, 0.40) * | 0.30 (0.28, 0.33) * | 0.26 (0.23, 0.29) * |  |
| **Heart failure mortality** | |  |  |  | 0.564 |  |  |  | 0.592 |
| MUO | 924 (1) | 1.0 (Reference) | 0.88 (0.75, 1.03) | 0.78 (0.63, 0.95) * |  | 1.0 (Reference) | 0.88 (0.75, 1.03) | 0.78 (0.63, 0.95) * |  |
| MUOW | 587 (0.6) | 0.58 (0.47, 0.72) * | 0.44 (0.37, 0.52) * | 0.33 (0.26, 0.43) * |  | 0.59 (0.48, 0.73) * | 0.45 (0.38, 0.53) * | 0.34 (0.27, 0.44) * |  |
| MUN | 202 (0.6) | 0.58 (0.42, 0.81) * | 0.45 (0.36, 0.57) * | 0.51 (0.37, 0.70) * |  | 0.59 (0.43, 0.82) * | 0.46 (0.36, 0.58) * | 0.52 (0.37, 0.71) * |  |
| MHO | 28 (0.3) | 0.42 (0.19, 0.96) * | 0.35 (0.21, 0.57) * | 0.37 (0.16, 0.84) * |  | 0.45 (0.20, 1.02) | 0.37 (0.22, 0.61) * | 0.40 (0.18, 0.90) * |  |
| MHOW | 165 (0.2) | 0.25 (0.17, 0.37) * | 0.27 (0.21, 0.34) * | 0.21 (0.15, 0.31) * |  | 0.28 (0.19, 0.41) * | 0.29 (0.23, 0.37) * | 0.23 (0.16, 0.34) * |  |
| MHN | 190 (0.2) | 0.36 (0.26, 0.49) * | 0.24 (0.19, 0.31) * | 0.24 (0.17, 0.34) * |  | 0.39 (0.29, 0.53) * | 0.27 (0.21, 0.34) * | 0.26 (0.18, 0.37) * |  |
| **Atrial fibrillation** |  |  |  |  | 0.015 |  |  |  | 0.014 |
| MUO | 9785 (10.9) | 1.0 (Reference) | 0.55 (0.52, 0.57) * | 0.31 (0.29, 0.34) * |  | 1.0 (Reference) | 0.55 (0.52, 0.57) * | 0.31 (0.29, 0.34) * |  |
| MUOW | 8201 (8.2) | 0.67 (0.63, 0.70) * | 0.34 (0.33, 0.36) * | 0.20 (0.18, 0.21) * |  | 0.67 (0.64, 0.71) * | 0.34 (0.33, 0.36) * | 0.20 (0.18, 0.21) * |  |
| MUN | 2194 (6.7) | 0.54 (0.49, 0.58) * | 0.30 (0.28, 0.32) * | 0.15 (0.13, 0.17) * |  | 0.54 (0.50, 0.58) * | 0.30 (0.28, 0.32) * | 0.15 (0.13, 0.17) * |  |
| MHO | 659 (6) | 0.92 (0.81, 1.05) | 0.41 (0.37, 0.46) * | 0.22 (0.17, 0.29) * |  | 0.93 (0.82, 1.06) | 0.42 (0.37, 0.47) * | 0.23 (0.18, 0.29) * |  |
| MHOW | 4105 (5.3) | 0.62 (0.58, 0.66) * | 0.30 (0.29, 0.32) * | 0.17 (0.15, 0.19) * |  | 0.63 (0.59, 0.67) * | 0.31 (0.29, 0.33) * | 0.17 (0.16, 0.19) * |  |
| MHN | 4348 (4.2) | 0.56 (0.52, 0.59) * | 0.28 (0.27, 0.30) * | 0.15 (0.13, 0.16) * |  | 0.57 (0.53, 0.61) * | 0.29 (0.27, 0.30) * | 0.15 (0.14, 0.17) * |  |
| **Atrial fibrillation mortality** | |  |  |  | 0.118 |  |  |  | 0.111 |
| MUO | 488 (0.5) | 1.0 (Reference) | 0.56 (0.46, 0.68) * | 0.34 (0.25, 0.47) * |  | 1.0 (Reference) | 0.56 (0.46, 0.68) * | 0.34 (0.25, 0.47) * |  |
| MUOW | 346 (0.3) | 0.55 (0.43, 0.70) * | 0.29 (0.24, 0.36) * | 0.19 (0.13, 0.26) * |  | 0.56 (0.44, 0.71) * | 0.30 (0.24, 0.37) * | 0.19 (0.14, 0.27) * |  |
| MUN | 101 (0.3) | 0.52 (0.36, 0.75) * | 0.29 (0.22, 0.39) * | 0.10 (0.05, 0.21) * |  | 0.53 (0.36, 0.76) * | 0.30 (0.22, 0.40) * | 0.10 (0.05, 0.22) * |  |
| MHO | 28 (0.3) | 1.00 (0.55, 1.79) | 0.41 (0.24, 0.71) * | 0.19 (0.05, 0.76) * |  | 1.03 (0.57, 1.86) | 0.43 (0.25, 0.74) * | 0.20 (0.05, 0.80) * |  |
| MHOW | 111 (0.1) | 0.38 (0.26, 0.54) * | 0.19 (0.14, 0.25) * | 0.12 (0.07, 0.22) * |  | 0.40 (0.28, 0.57) * | 0.20 (0.15, 0.27) * | 0.13 (0.07, 0.23) * |  |
| MHN | 152 (0.1) | 0.49 (0.36, 0.66) * | 0.26 (0.20, 0.34) * | 0.04 (0.02, 0.10) * |  | 0.51 (0.38, 0.70) * | 0.28 (0.21, 0.36) * | 0.04 (0.02, 0.11) * |  |

Note, BMI, body mass index; MH status was defined as < 3 abnormal components; MH, metabolically healthy; MHN, metabolically healthy normal weight; MHOW, metabolically healthy overweight; MHO, metabolically healthy obesity; MUN, metabolically unhealthy normal weight; MUOW, metabolically unhealthy overweight; MUO, metabolically unhealthy obesity; PRS, polygenic risk score; the PRSs presented are specifically used based on corresponding outcomes; Model 1 was adjusted for age, sex, race, Townsend Deprivation Index, annual household income, education attainment, 22 assessment centers, and the first 5 principal components of ancestry. Model 2 was further adjusted for family history of diabetes, family history of high blood pressure, and lifestyle factors including sleep duration, healthy diet, physical activity, smoking status, and alcohol intake frequency, based on Model 1; ^a^ likelihood tests was applied to test the significance of interaction term by comparing the model with and without the interaction term; **P* < 0.05.

**Table S15 Combined effects of BMI-metabolic status (MH status: < 1 abnormal component) and PRSs on the risk of cardiovascular outcomes and all-cause mortality**

| **Outcomes** | **Case (%)** | **Model 1** | | | | **Model 2** | | | |
| --- | --- | --- | --- | --- | --- | --- | --- | --- | --- |
|  |  | **High PRS** | **Moderate PRS** | **Low PRS** | ***P* for interaction ^a^** | **High PRS** | **Moderate PRS** | **Low PRS** | ***P* for interaction ^a^** |
| **All-cause mortality** |  |  |  |  | 0.204 |  |  |  | 0.142 |
| MUO | 8381 (9.4) | 1.0 (Reference) | 0.97 (0.92, 1.02) | 0.87 (0.82, 0.94) * |  | 1.0 (Reference) | 0.96 (0.91, 1.01) | 0.87 (0.81, 0.93) * |  |
| MUOW | 11 098 (7.8) | 0.79 (0.75, 0.85) * | 0.75 (0.71, 0.79) * | 0.68 (0.64, 0.73) * |  | 0.81 (0.76, 0.86) * | 0.76 (0.72, 0.80) * | 0.69 (0.65, 0.74) * |  |
| MUN | 6240 (7.3) | 0.84 (0.78, 0.91) * | 0.76 (0.72, 0.81) * | 0.78 (0.72, 0.84) * |  | 0.85 (0.79, 0.92) * | 0.77 (0.73, 0.82) * | 0.80 (0.74, 0.86) * |  |
| MHO | 38 (2.8) | 0.62 (0.30, 1.31) | 0.46 (0.31, 0.70) * | 0.50 (0.25, 0.99) * |  | 0.62 (0.30, 1.31) | 0.49 (0.32, 0.74) * | 0.52 (0.26, 1.04) |  |
| MHOW | 706 (3.5) | 0.63 (0.52, 0.76) * | 0.57 (0.52, 0.64) * | 0.53 (0.45, 0.62) * |  | 0.65 (0.54, 0.79) * | 0.59 (0.53, 0.66) * | 0.55 (0.47, 0.64) * |  |
| MHN | 1453 (3.5) | 0.69 (0.60, 0.79) * | 0.61 (0.56, 0.66) * | 0.63 (0.56, 0.71) * |  | 0.70 (0.61, 0.81) * | 0.63 (0.58, 0.68) * | 0.66 (0.59, 0.74) * |  |
| **Cardiovascular events** |  |  |  |  | 0.014 |  |  |  | 0.013 |
| MUO | 18 977 (20.4) | 1.0 (Reference) | 0.77 (0.75, 0.80) * | 0.62 (0.59, 0.65) * |  | 1.0 (Reference) | 0.77 (0.75, 0.80) * | 0.62 (0.60, 0.65) * |  |
| MUOW | 24 326 (16.3) | 0.75 (0.72, 0.78) * | 0.55 (0.53, 0.56) * | 0.42 (0.40, 0.44) * |  | 0.77 (0.74, 0.80) * | 0.56 (0.54, 0.58) * | 0.43 (0.41, 0.45) * |  |
| MUN | 11 400 (12.8) | 0.64 (0.61, 0.67) * | 0.46 (0.44, 0.48) * | 0.38 (0.36, 0.40) * |  | 0.65 (0.62, 0.69) * | 0.48 (0.46, 0.49) * | 0.39 (0.37, 0.41) * |  |
| MHO | 113 (8.2) | 0.62 (0.41, 0.94) * | 0.47 (0.37, 0.59) * | 0.36 (0.23, 0.56) * |  | 0.64 (0.42, 0.97) * | 0.48 (0.38, 0.61) * | 0.37 (0.23, 0.58) * |  |
| MHOW | 1500 (7.3) | 0.49 (0.43, 0.55) * | 0.39 (0.37, 0.42) * | 0.28 (0.25, 0.32) * |  | 0.50 (0.45, 0.57) * | 0.41 (0.38, 0.44) * | 0.29 (0.26, 0.33) * |  |
| MHN | 2501 (5.8) | 0.41 (0.38, 0.46) * | 0.33 (0.31, 0.35) * | 0.28 (0.25, 0.30) * |  | 0.43 (0.39, 0.47) * | 0.35 (0.33, 0.37) * | 0.29 (0.27, 0.32) * |  |
| **Cardiovascular mortality** | |  |  |  | 0.395 |  |  |  | 0.446 |
| MUO | 2487 (2.7) | 1.0 (Reference) | 0.72 (0.66, 0.79) * | 0.58 (0.51, 0.66) * |  | 1.0 (Reference) | 0.72 (0.65, 0.79) * | 0.58 (0.51, 0.66) * |  |
| MUOW | 2845 (1.9) | 0.68 (0.61, 0.75) * | 0.48 (0.44, 0.53) * | 0.35 (0.31, 0.40) * |  | 0.69 (0.63, 0.77) * | 0.49 (0.45, 0.54) * | 0.36 (0.32, 0.41) * |  |
| MUN | 1459 (1.6) | 0.73 (0.64, 0.83) * | 0.46 (0.42, 0.51) * | 0.39 (0.33, 0.45) * |  | 0.74 (0.65, 0.84) * | 0.48 (0.43, 0.53) * | 0.40 (0.35, 0.47) * |  |
| MHO | 5 (0.4) | 0.56 (0.14, 2.24) | 0.19 (0.06, 0.59) * | - |  | 0.55 (0.14, 2.22) | 0.20 (0.07, 0.63) * | - |  |
| MHOW | 120 (0.6) | 0.39 (0.26, 0.59) * | 0.28 (0.21, 0.35) * | 0.28 (0.19, 0.40) * |  | 0.41 (0.27, 0.62) * | 0.29 (0.23, 0.37) * | 0.29 (0.20, 0.43) * |  |
| MHN | 206 (0.5) | 0.42 (0.31, 0.56) * | 0.26 (0.21, 0.31) * | 0.25 (0.19, 0.34) * |  | 0.43 (0.32, 0.59) * | 0.27 (0.22, 0.33) * | 0.27 (0.20, 0.36) * |  |
| **Coronary disease** |  |  |  |  | 0.021 |  |  |  | 0.022 |
| MUO | 11 523 (12.2) | 1.0 (Reference) | 0.68 (0.65, 0.71) * | 0.46 (0.43, 0.48) * |  | 1.0 (Reference) | 0.68 (0.66, 0.71) * | 0.46 (0.43, 0.49) * |  |
| MUOW | 14 756 (9.7) | 0.79 (0.75, 0.83) * | 0.49 (0.47, 0.51) * | 0.31 (0.29, 0.33) * |  | 0.81 (0.78, 0.85) * | 0.51 (0.49, 0.53) * | 0.32 (0.30, 0.34) * |  |
| MUN | 6252 (6.9) | 0.62 (0.59, 0.66) * | 0.39 (0.37, 0.41) * | 0.26 (0.24, 0.28) * |  | 0.65 (0.61, 0.69) * | 0.40 (0.38, 0.42) * | 0.27 (0.25, 0.29) * |  |
| MHO | 62 (4.5) | 0.47 (0.27, 0.82) * | 0.40 (0.29, 0.55) * | 0.25 (0.14, 0.47) * |  | 0.49 (0.29, 0.85) * | 0.42 (0.31, 0.58) * | 0.27 (0.14, 0.50) * |  |
| MHOW | 751 (3.6) | 0.43 (0.37, 0.50) * | 0.29 (0.26, 0.32) * | 0.19 (0.16, 0.23) * |  | 0.45 (0.39, 0.53) * | 0.31 (0.28, 0.34) * | 0.20 (0.17, 0.24) * |  |
| MHN | 1153 (2.7) | 0.39 (0.35, 0.44) * | 0.23 (0.21, 0.25) * | 0.16 (0.14, 0.18) * |  | 0.41 (0.37, 0.47) * | 0.25 (0.23, 0.27) * | 0.17 (0.15, 0.20) * |  |
| **Coronary disease mortality** | |  |  |  | 0.032 |  |  |  | 0.037 |
| MUO | 1558 (1.6) | 1.0 (Reference) | 0.65 (0.58, 0.72) * | 0.43 (0.37, 0.51) * |  | 1.0 (Reference) | 0.64 (0.57, 0.72) * | 0.43 (0.36, 0.51) * |  |
| MUOW | 1782 (1.2) | 0.71 (0.62, 0.80) * | 0.44 (0.39, 0.49) * | 0.23 (0.20, 0.28) * |  | 0.72 (0.64, 0.82) * | 0.45 (0.40, 0.50) * | 0.24 (0.20, 0.28) * |  |
| MUN | 779 (0.9) | 0.59 (0.50, 0.69) * | 0.40 (0.35, 0.45) * | 0.24 (0.19, 0.30) * |  | 0.60 (0.51, 0.71) * | 0.41 (0.36, 0.46) * | 0.25 (0.20, 0.31) * |  |
| MHO | 2 (0.1) | 0.75 (0.19, 3.00) | - | - |  | 0.76 (0.19, 3.04) | - | - |  |
| MHOW | 60 (0.3) | 0.34 (0.20, 0.57) * | 0.20 (0.14, 0.28) * | 0.20 (0.12, 0.35) * |  | 0.36 (0.21, 0.61) * | 0.21 (0.14, 0.30) * | 0.22 (0.13, 0.37) * |  |
| MHN | 96 (0.2) | 0.35 (0.23, 0.52) * | 0.17 (0.13, 0.23) * | 0.19 (0.13, 0.30) * |  | 0.36 (0.24, 0.54) * | 0.18 (0.14, 0.25) * | 0.20 (0.13, 0.31) * |  |
| **Myocardial infarction** |  |  |  |  | 0.213 |  |  |  | 0.156 |
| MUO | 5828 (5.9) | 1.0 (Reference) | 0.74 (0.70, 0.79) * | 0.57 (0.53, 0.62) * |  | 1.0 (Reference) | 0.74 (0.70, 0.79) * | 0.58 (0.53, 0.62) * |  |
| MUOW | 7387 (4.7) | 0.79 (0.74, 0.84) * | 0.55 (0.52, 0.58) * | 0.39 (0.36, 0.42) * |  | 0.82 (0.77, 0.88) * | 0.57 (0.54, 0.61) * | 0.40 (0.37, 0.43) * |  |
| MUN | 2957 (3.2) | 0.62 (0.57, 0.67) * | 0.44 (0.41, 0.47) * | 0.32 (0.29, 0.36) * |  | 0.65 (0.60, 0.71) * | 0.46 (0.43, 0.49) * | 0.33 (0.30, 0.37) * |  |
| MHO | 18 (1.3) | 0.50 (0.22, 1.12) | 0.26 (0.14, 0.48) * | 0.13 (0.03, 0.52) * |  | 0.53 (0.24, 1.19) * | 0.27 (0.15, 0.51) * | 0.14 (0.03, 0.55) * |  |
| MHOW | 269 (1.3) | 0.31 (0.24, 0.41) * | 0.25 (0.21, 0.30) * | 0.21 (0.16, 0.29) * |  | 0.34 (0.26, 0.44) * | 0.27 (0.23, 0.32) * | 0.23 (0.17, 0.30) * |  |
| MHN | 424 (1) | 0.28 (0.23, 0.35) * | 0.22 (0.19, 0.25) * | 0.19 (0.15, 0.25) * |  | 0.31 (0.25, 0.37) * | 0.24 (0.21, 0.27) * | 0.21 (0.16, 0.26) * |  |
| **Myocardial infarction mortality** | |  |  |  | 0.439 |  |  |  | 0.429 |
| MUO | 626 (0.6) | 1.0 (Reference) | 0.75 (0.62, 0.90) * | 0.53 (0.41, 0.68) * |  | 1.0 (Reference) | 0.75 (0.62, 0.90) * | 0.53 (0.41, 0.69) * |  |
| MUOW | 705 (0.5) | 0.57 (0.46, 0.71) * | 0.54 (0.45, 0.64) * | 0.32 (0.25, 0.41) * |  | 0.59 (0.47, 0.74) * | 0.56 (0.47, 0.67) * | 0.33 (0.25, 0.43) * |  |
| MUN | 315 (0.3) | 0.56 (0.42, 0.73) * | 0.48 (0.39, 0.60) * | 0.30 (0.21, 0.42) * |  | 0.59 (0.45, 0.77) * | 0.51 (0.41, 0.62) * | 0.31 (0.22, 0.44) * |  |
| MHO | 0 (0.0) | - | - | - |  | - | - | - |  |
| MHOW | 21 (0.1) | 0.24 (0.09, 0.65) * | 0.19 (0.10, 0.35) * | 0.30 (0.13, 0.67) * |  | 0.26 (0.10, 0.70) * | 0.20 (0.11, 0.37) * | 0.32 (0.14, 0.72) * |  |
| MHN | 26 (0.1) | 0.15 (0.06, 0.38) * | 0.14 (0.08, 0.24) * | 0.22 (0.10, 0.47) * |  | 0.17 (0.07, 0.40) * | 0.15 (0.09, 0.26) * | 0.24 (0.11, 0.51) * |  |
| **Stroke** |  |  |  |  | 0.108 |  |  |  | 0.110 |
| MUO | 3185 (3.2) | 1.0 (Reference) | 0.95 (0.87, 1.04) | 0.80 (0.72, 0.90) * |  | 1.0 (Reference) | 0.95 (0.87, 1.04) | 0.81 (0.72, 0.90) * |  |
| MUOW | 4489 (2.8) | 0.87 (0.79, 0.96) * | 0.77 (0.71, 0.84) * | 0.78 (0.70, 0.86) * |  | 0.89 (0.81, 0.98) * | 0.78 (0.72, 0.85) * | 0.79 (0.72, 0.88) * |  |
| MUN | 2404 (2.6) | 0.90 (0.81, 1.01) | 0.80 (0.73, 0.87) * | 0.73 (0.64, 0.82) * |  | 0.93 (0.83, 1.04) | 0.82 (0.74, 0.90) * | 0.75 (0.66, 0.84) * |  |
| MHO | 12 (0.9) | 0.16 (0.02, 1.16) | 0.56 (0.30, 1.05) | 0.18 (0.02, 1.26) |  | 0.16 (0.02, 1.17) | 0.59 (0.31, 1.09) | 0.19 (0.03, 1.35) |  |
| MHOW | 253 (1.2) | 0.49 (0.36, 0.66) * | 0.57 (0.48, 0.67) * * | 0.52 (0.39, 0.70) * |  | 0.51 (0.37, 0.69) * | 0.59 (0.49, 0.70) * | 0.54 (0.41, 0.72) * |  |
| MHN | 426 (1.0) | 0.50 (0.40, 0.63) * | 0.50 (0.43, 0.57) | 0.43 (0.34, 0.55) * |  | 0.52 (0.42, 0.66) * | 0.52 (0.45, 0.60) * | 0.45 (0.36, 0.57) * |  |
| **Stroke mortality** |  |  |  |  | 0.166 |  |  |  | 0.154 |
| MUO | 651 (0.6) | 1.0 (Reference) | 0.95 (0.79, 1.16) | 0.75 (0.58, 0.96) * |  | 1.0 (Reference) | 0.96 (0.79, 1.16) | 0.75 (0.58, 0.96) * |  |
| MUOW | 894 (0.6) | 0.92 (0.74, 1.14) | 0.73 (0.61, 0.88) * | 0.72 (0.58, 0.90) * |  | 0.95 (0.77, 1.18) | 0.75 (0.62, 0.91) * | 0.74 (0.59, 0.93) * |  |
| MUN | 563 (0.6) | 0.98 (0.77, 1.26) | 0.93 (0.76, 1.13) | 0.79 (0.61, 1.03) |  | 1.02 (0.80, 1.30) | 0.96 (0.79, 1.17) | 0.82 (0.63, 1.07) |  |
| MHO | 4 (0.3) | - | 1.00 (0.32, 3.13) | 1.03 (0.14, 7.37) |  | - | 1.05 (0.33, 3.30) | 1.16 (0.16, 8.29) |  |
| MHOW | 43 (0.2) | 0.74 (0.41, 1.34) | 0.52 (0.34, 0.79) * | 0.28 (0.12, 0.70) * |  | 0.79 (0.44, 1.43) | 0.55 (0.36, 0.84) * | 0.30 (0.12, 0.73) * |  |
| MHN | 81 (0.2) | 0.31 (0.16, 0.61) * | 0.58 (0.42, 0.79) * | 0.53 (0.32, 0.87) * |  | 0.33 (0.17, 0.65) * | 0.61 (0.45, 0.84) * | 0.56 (0.34, 0.93) * |  |
| **Heart failure** |  |  |  |  | 0.904 |  |  |  | 0.912 |
| MUO | 6205 (6.2) | 1.0 (Reference) | 0.85 (0.80, 0.91) * | 0.77 (0.71, 0.83) * |  | 1.0 (Reference) | 0.85 (0.80, 0.90) * | 0.77 (0.71, 0.83) * |  |
| MUOW | 5611 (3.5) | 0.55 (0.51, 0.60) * | 0.45 (0.42, 0.48) * | 0.39 (0.36, 0.43) * |  | 0.57 (0.53, 0.62) * | 0.46 (0.44, 0.49) * | 0.41 (0.37, 0.44) * |  |
| MUN | 2332 (2.5) | 0.43 (0.39, 0.48) * | 0.37 (0.34, 0.40) * | 0.32 (0.29, 0.36) * |  | 0.45 (0.41, 0.50) * | 0.38 (0.36, 0.41) * | 0.34 (0.30, 0.38) * |  |
| MHO | 23 (1.6) | 0.56 (0.25, 1.24) | 0.44 (0.26, 0.74) * | 0.26 (0.09, 0.82) * |  | 0.58 (0.26, 1.30) | 0.46 (0.27, 0.79) * | 0.29 (0.09, 0.89) * |  |
| MHOW | 242 (1.2) | 0.28 (0.21, 0.38) * | 0.28 (0.24, 0.33) * | 0.25 (0.19, 0.33) * |  | 0.30 (0.23, 0.40) * | 0.30 (0.25, 0.35) * | 0.26 (0.20, 0.35) * |  |
| MHN | 405 (0.9) | 0.27 (0.22, 0.34) * | 0.25 (0.22, 0.28) * | 0.23 (0.19, 0.29) * |  | 0.29 (0.23, 0.36) * | 0.27 (0.23, 0.31) * | 0.25 (0.20, 0.31) * |  |
| **Heart failure mortality** |  |  |  |  | 0.557 |  |  |  | 0.586 |
| MUO | 952 (0.9) | 1.0 (Reference) | 0.88 (0.75, 1.03) | 0.78 (0.64, 0.95) * |  | 1.0 (Reference) | 0.87 (0.75, 1.02) | 0.78 (0.64, 0.95) * |  |
| MUOW | 729 (0.5) | 0.51 (0.42, 0.62) * | 0.40 (0.34, 0.47) * | 0.31 (0.25, 0.39) * |  | 0.53 (0.44, 0.65) * | 0.42 (0.35, 0.49) * | 0.33 (0.26, 0.41) * |  |
| MUN | 340 (0.4) | 0.47 (0.36, 0.61) * | 0.35 (0.29, 0.43) * | 0.39 (0.30, 0.50) * |  | 0.49 (0.38, 0.64) * | 0.37 (0.31, 0.45) * | 0.40 (0.31, 0.53) * |  |
| MHO | 0 (0.0) | - | - | - |  | - | - | - |  |
| MHOW | 23 (0.1) | 0.14 (0.05, 0.45) * | 0.25 (0.15, 0.42) * | 0.12 (0.04, 0.38) * |  | 0.16 (0.05, 0.49) * | 0.28 (0.17, 0.45) * | 0.13 (0.04, 0.41) * |  |
| MHN | 52 (0.1) | 0.40 (0.24, 0.66) * | 0.24 (0.16, 0.35) * | 0.19 (0.09, 0.39) * |  | 0.43 (0.26, 0.72) * | 0.26 (0.18, 0.39) * | 0.21 (0.10, 0.42) * |  |
| **Atrial fibrillation** |  |  |  |  | 0.031 |  |  |  | 0.029 |
| MUO | 10 393 (10.4) | 1.0 (Reference) | 0.54 (0.52, 0.56) * | 0.31 (0.29, 0.33) * |  | 1.0 (Reference) | 0.54 (0.52, 0.56) * | 0.31 (0.29, 0.33) * |  |
| MUOW | 11 567 (7.4) | 0.66 (0.63, 0.69) * | 0.33 (0.32, 0.35) * | 0.19 (0.18, 0.20) * |  | 0.66 (0.63, 0.69) * | 0.34 (0.32, 0.35) * | 0.19 (0.18, 0.20) * |  |
| MUN | 5 278 (5.7) | 0.56 (0.53, 0.59) * | 0.30 (0.28, 0.31) * | 0.15 (0.14, 0.17) * |  | 0.56 (0.53, 0.60) * | 0.30 (0.29, 0.32) * | 0.15 (0.14, 0.17) * |  |
| MHO | 51 (3.7) | 0.88 (0.59, 1.30) | 0.27 (0.18, 0.41) * | 0.14 (0.05, 0.37) * |  | 0.89 (0.60, 1.32) | 0.27 (0.18, 0.41) * | 0.14 (0.05, 0.38) * |  |
| MHOW | 739 (3.6) | 0.59 (0.52, 0.67) * | 0.27 (0.25, 0.30) * | 0.18 (0.15, 0.22) * |  | 0.60 (0.53, 0.69) * | 0.28 (0.25, 0.31) * | 0.18 (0.15, 0.23) * |  |
| MHN | 1 264 (2.9) | 0.54 (0.49, 0.60) * | 0.26 (0.24, 0.28) * | 0.14 (0.11, 0.16) * |  | 0.55 (0.50, 0.61) * | 0.26 (0.24, 0.28) * | 0.14 (0.12, 0.16) * |  |
| **Atrial fibrillation mortality** | |  |  |  | 0.007 |  |  |  | 0.006 |
| MUO | 515 (0.5) | 1.0 (Reference) | 0.55 (0.45, 0.66) * | 0.33 (0.25, 0.45) * |  | 1.0 (Reference) | 0.55 (0.45, 0.66) * | 0.33 (0.25, 0.45) * |  |
| MUOW | 446 (0.3) | 0.50 (0.40, 0.62) * | 0.27 (0.22, 0.33) * | 0.17 (0.12, 0.23) * |  | 0.51 (0.41, 0.64) * | 0.28 (0.23, 0.34) * | 0.17 (0.12, 0.23) * |  |
| MUN | 224 (0.2) | 0.52 (0.40, 0.68) * | 0.29 (0.23, 0.36) * | 0.08 (0.04, 0.14) * |  | 0.54 (0.41, 0.70) * | 0.30 (0.24, 0.38) * | 0.08 (0.04, 0.14) * |  |
| MHO | 1 (0.1) | - | 0.36 (0.05, 2.60) | - |  | - | 0.39 (0.05, 2.75) * | - |  |
| MHOW | 11 (0.1) | 0.39 (0.17, 0.87) * | 0.04 (0.01, 0.15) * | 0.16 (0.05, 0.51) * |  | 0.41 (0.18, 0.94) * | 0.04 (0.01, 0.16) * | 0.17 (0.05, 0.54) * |  |
| MHN | 29 (0.1) | 0.38 (0.20, 0.69) * | 0.19 (0.11, 0.30) * | - |  | 0.40 (0.22, 0.74) * | 0.20 (0.12, 0.32) * | - |  |

Note, BMI, body mass index; MH status was defined as < 3 abnormal components; MH, metabolically healthy; MHN, metabolically healthy normal weight; MHOW, metabolically healthy overweight; MHO, metabolically healthy obesity; MUN, metabolically unhealthy normal weight; MUOW, metabolically unhealthy overweight; MUO, metabolically unhealthy obesity; PRS, polygenic risk score; the PRSs presented are specifically used based on corresponding outcomes; Model 1 was adjusted for age, sex, race, Townsend Deprivation Index, annual household income, education attainment, 22 assessment centers, and the first 5 principal components of ancestry. Model 2 was further adjusted for family history of diabetes, family history of high blood pressure, and lifestyle factors including sleep duration, healthy diet, physical activity, smoking status, and alcohol intake frequency, based on Model 1; **^a^** likelihood tests was applied to test the significance of interaction term by comparing the model with and without the interaction term; **P* < 0.05.

**Table S16 Transitions of BMI-metabolic status (MH status: < 3 abnormal components) from baseline to the second resurvey**

| BMI-metabolic status | BMI-metabolic status at the second resurvey, number of participants (%) | | | | |
| --- | --- | --- | --- | --- | --- |
|  | MHN | MHOO | MUN | MUOO | Total |
| MHN | 3521 (80.4) | 558 (12.7) | 207 (4.7) | 94 (2.2) | 4380 (100.0) |
| MHOO | 498 (9.6) | 3522 (68.2) | 34 (0.7) | 1107 (21.4) | 5161 (100.0) |
| MUN | 105 (28.9) | 24 (6.6) | 170 (46.8) | 64 (17.6) | 363 (100.0) |
| MUOO | 76 (2.4) | 724 (22.7) | 76 (2.4) | 2313 (72.5) | 3189 (100.0) |
| Total | 4200 (32.1) | 4828 (36.9) | 487 (3.7) | 3578 (27.3) | 13 093 (100.0) |

Note, BMI, body mass index; MH, metabolically healthy; MHN, metabolically healthy normal weight; MHOO, metabolically healthy overweight or obesity; MUN, metabolically unhealthy normal weight; MUOO, metabolically unhealthy overweight or obesity

**Table S17 Combined effects of transitions in BMI-metabolic status (MH status: < 3 abnormal components) and PRSs on the risk of specific cardiovascular outcomes**

| **Outcomes** | **Case (%)** | **Model 1** | | | | **Model 2** | | | |
| --- | --- | --- | --- | --- | --- | --- | --- | --- | --- |
|  |  | **High PRS** | **Moderate PRS** | **Low PRS** | ***P* for interaction ^a^** | **High PRS** | **Moderate PRS** | **Low PRS** | ***P* for interaction ^a^** |
| **Coronary disease** |  |  |  |  | 0.635 |  |  |  | 0.630 |
| MUOO throughout | 216 (11.1) | 1.0 (Reference) | 0.60 (0.45, 0.81) * | 0.38 (0.24, 0.61) * |  | 1.0 (Reference) | 0.60 (0.45, 0.81) * | 0.38 (0.24, 0.60) * |  |
| MHOO to MUOO | 97 (9.4) | 0.69 (0.41, 1.15) | 0.60 (0.43, 0.85) * | 0.47 (0.27, 0.81) * |  | 0.70 (0.42, 1.18) | 0.61 (0.43, 0.87) * | 0.48 (0.28, 0.83) * |  |
| MHOO throughout | 152 (4.4) | 0.47 (0.31, 0.71) * | 0.33 (0.24, 0.45) * | 0.19 (0.12, 0.31) * |  | 0.49 (0.32, 0.74) * | 0.34 (0.24, 0.46) * | 0.20 (0.12, 0.32) * |  |
| MHN to MHOO | 24 (4.4) | 0.61 (0.29, 1.27) | 0.25 (0.13, 0.47) * | 0.32 (0.13, 0.80) * |  | 0.63 (0.30, 1.32) | 0.26 (0.13, 0.48) * | 0.34 (0.14, 0.85) * |  |
| MHN throughout | 137 (3.9) | 0.43 (0.28, 0.66) * | 0.33 (0.24, 0.46) * | 0.16 (0.09, 0.28) * |  | 0.46 (0.30, 0.70) * | 0.35 (0.25, 0.49) * | 0.17 (0.10, 0.29) * |  |
| **Coronary disease mortality** | |  |  |  | 0.504 |  |  |  | 0.554 |
| MUOO throughout | 39 (1.9) | 1.0 (Reference) | 0.91 (0.43, 1.93) | 0.61 (0.21, 1.80) |  | 1.0 (Reference) | 0.92 (0.44, 1.95) | 0.60 (0.20, 1.76) |  |
| MHOO to MUOO | 9 (0.8) | 0.64 (0.14, 2.97) | 0.57 (0.21, 1.51) | - |  | 0.70 (0.15, 3.22) | 0.58 (0.22, 1.54) | - |  |
| MHOO throughout | 15 (0.4) | 0.62 (0.21, 1.86) | 0.20 (0.07, 0.55) * | 0.34 (0.11, 1.11) |  | 0.73 (0.24, 2.17) | 0.21 (0.07, 0.60) * | 0.40 (0.12, 1.29) |  |
| MHN to MHOO | 4 (0.7) | 0.75 (0.10, 5.97) | 0.43 (0.09, 2.01) | 0.68 (0.09, 5.39) |  | 0.82 (0.10, 6.46) | 0.51 (0.11, 2.39) | 0.81 (0.10, 6.45) |  |
| MHN throughout | 11 (0.3) | 0.30 (0.06, 1.39) | 0.29 (0.11, 0.79) * | 0.23 (0.05, 1.05) |  | 0.36 (0.08, 1.67) | 0.34 (0.13, 0.92) * | 0.26 (0.06, 1.22) |  |
| **Myocardial infarction** |  |  |  |  | 0.063 |  |  |  | 0.066 |
| MUOO throughout | 157 (7.2) | 1.0 (Reference) | 0.93 (0.64, 1.35) | 0.48 (0.27, 0.88) * |  | 1.0 (Reference) | 0.91 (0.62, 1.33) | 0.47 (0.26, 0.86) * |  |
| MHOO to MUOO | 42 (3.9) | 1.04 (0.58, 1.85) | 0.42 (0.25, 0.73) * | 0.33 (0.13, 0.84) * |  | 1.06 (0.59, 1.88) | 0.43 (0.25, 0.75) * | 0.33 (0.13, 0.85) * |  |
| MHOO throughout | 69 (2.0) | 0.37 (0.20, 0.67) * | 0.29 (0.19, 0.47) * | 0.37 (0.21, 0.67) * |  | 0.38 (0.21, 0.70) * | 0.31 (0.19, 0.49) | 0.39 (0.22, 0.69) * |  |
| MHN to MHOO | 9 (1.6) | 0.14 (0.02, 1.03) | 0.44 (0.20, 0.95) * | - |  | 0.14 (0.02, 1.06) | 0.46 (0.21, 1.00) | - |  |
| MHN throughout | 52 (1.5) | 0.39 (0.21, 0.72) * | 0.27 (0.17, 0.44) * | 0.20 (0.09, 0.46) * |  | 0.40 (0.22, 0.75) * | 0.28 (0.17, 0.46) * | 0.21 (0.09, 0.48) * |  |
| **Myocardial infarction mortality** | |  |  |  | 0.062 |  |  |  | 0.061 |
| MUOO throughout | 20 (0.9) | 1.0 (Reference) | 1.35 (0.38, 4.83) | 2.11 (0.50, 8.89) |  | 1.0 (Reference) | 1.34 (0.37, 4.85) | 2.14 (0.50, 9.10) |  |
| MHOO to MUOO | 1 (0.1) | - | 0.27 (0.03, 2.63) | - |  | - | 0.29 (0.03, 2.84) | - |  |
| MHOO throughout | 9 (0.3) | 0.97 (0.19, 4.92) | 0.28 (0.06, 1.44) | 0.80 (0.16, 4.09) |  | 1.09 (0.21, 5.61) | 0.32 (0.06, 1.65) | 0.90 (0.17, 4.69) |  |
| MHN to MHOO | 4 (0.7) | - | 2.73 (0.59, 12.64) | - |  | - | 3.09 (0.65, 14.59) | - |  |
| MHN throughout | 2 (0.1) | 0.35 (0.04, 3.45) | 0.11 (0.01, 1.06) | - |  | 0.39 (0.04, 3.90) | 0.12 (0.01, 1.22) | - |  |
| **Stroke** |  |  |  |  | 0.922 |  |  |  | 0.902 |
| MUOO throughout | 85 (3.7) | 1.0 (Reference) | 0.89 (0.51, 1.54) | 0.90 (0.46, 1.75) |  | 1.0 (Reference) | 0.87 (0.50, 1.51) | 0.90 (0.46, 1.75) |  |
| MHOO to MUOO | 27 (2.5) | 0.42 (0.12, 1.45) | 0.78 (0.40, 1.50) | 0.63 (0.23, 1.75) |  | 0.42 (0.12, 1.44) | 0.80 (0.42, 1.55) | 0.66 (0.24, 1.84) |  |
| MHOO throughout | 50 (1.4) | 0.59 (0.27, 1.26) | 0.46 (0.25, 0.84) * | 0.49 (0.22, 1.08) |  | 0.62 (0.29, 1.34) | 0.48 (0.26, 0.88) * | 0.52 (0.24, 1.15) |  |
| MHN to MHOO | 9 (1.6) | 1.02 (0.30, 3.49) | 0.44 (0.15, 1.32) | 0.52 (0.12, 2.25) |  | 1.09 (0.32, 3.75) | 0.47 (0.16, 1.41) | 0.55 (0.13, 2.39) |  |
| MHN throughout | 52 (1.5) | 0.54 (0.25, 1.20) | 0.59 (0.33, 1.07) | 0.44 (0.19, 1.03) |  | 0.58 (0.26, 1.29) | 0.64 (0.35, 1.17) | 0.48 (0.20, 1.13) |  |
| **Stroke mortality** |  |  |  |  | 0.318 |  |  |  | 0.410 |
| MUOO throughout | 15 (0.7) | 1.0 (Reference) | 0.25 (0.08, 0.74) * | 0.23 (0.05, 1.13) |  | 1.0 (Reference) | 0.24 (0.08, 0.72) | 0.23 (0.05, 1.12) |  |
| MHOO to MUOO | 4 (0.4) | - | 0.25 (0.06, 0.98) * | 0.31 (0.04, 2.51) |  | - | 0.25 (0.06, 0.99) * | 0.32 (0.04, 2.61) |  |
| MHOO throughout | 9 (0.3) | 0.31 (0.08, 1.25) | 0.16 (0.05, 0.53) * | 0.11 (0.01, 0.93) * |  | 0.33 (0.08, 1.33) | 0.17 (0.05, 0.57) * | 0.12 (0.01, 0.96) * |  |
| MHN to MHOO | 2 (0.4) | 0.67 (0.08, 5.59) | 0.22 (0.03, 1.79) | - |  | 0.69 (0.08, 5.78) | 0.23 (0.03, 1.96) | - |  |
| MHN throughout | 10 (0.3) | 0.11 (0.01, 0.91) * | 0.24 (0.08, 0.70) * | 0.21 (0.04, 1.05) |  | 0.12 (0.01, 0.99) * | 0.25 (0.08, 0.77) * | 0.23 (0.05, 1.18) |  |
| **Heart failure** |  |  |  |  | 0.076 |  |  |  | 0.085 |
| MUOO throughout | 134 (5.9) | 1.0 (Reference) | 0.63 (0.41, 0.96) * | 1.03 (0.63, 1.67) |  | 1.0 (Reference) | 0.63 (0.41, 0.96) * | 0.99 (0.61, 1.62) |  |
| MHOO to MUOO | 36 (3.3) | 0.44 (0.18, 1.05) | 0.52 (0.30, 0.90) * | 0.53 (0.24, 1.15) |  | 0.45 (0.19, 1.07) | 0.54 (0.31, 0.93) * | 0.53 (0.24, 1.15) |  |
| MHOO throughout | 70 (2.0) | 0.35 (0.18, 0.69) * | 0.41 (0.26, 0.65) * | 0.35 (0.18, 0.66) * |  | 0.37 (0.19, 0.72) * | 0.44 (0.27, 0.69) * | 0.36 (0.19, 0.70) * |  |
| MHN to MHOO | 11 (2.0) | - | 0.51 (0.24, 1.07) | 0.45 (0.11, 1.87) |  | - | 0.55 (0.26, 1.16) | 0.47 (0.11, 1.97) |  |
| MHN throughout | 48 (1.4) | 0.32 (0.15, 0.67) * | 0.27 (0.16, 0.46) * | 0.30 (0.15, 0.60) * |  | 0.34 (0.16, 0.72) * | 0.29 (0.17, 0.49) * | 0.32 (0.16, 0.65) * |  |
| **Heart failure mortality** |  |  |  |  | 0.375 |  |  |  | 0.613 |
| MUOO throughout | 25 (1.1) | 1.0 (Reference) | 1.06 (0.39, 2.86) | 0.41 (0.08, 2.11) |  | 1.0 (Reference) | 1.10 (0.40, 2.99) | 0.42 (0.08, 2.18) |  |
| MHOO to MUOO | 8 (0.7) | - | 0.81 (0.23, 2.83) | 1.43 (0.34, 6.02) |  | - | 0.88 (0.25, 3.07) | 1.58 (0.37, 6.70) |  |
| MHOO throughout | 5 (0.1) | 0.21 (0.02, 1.85) | 0.20 (0.05, 0.84) * | 0.19 (0.02, 1.64) |  | 0.24 (0.03, 2.08) | 0.22 (0.05, 0.92) * | 0.20 (0.02, 1.73) |  |
| MHN to MHOO | 1 (0.2) | - | 0.39 (0.05, 3.44) | - |  | - | 0.39 (0.04, 3.47) | - |  |
| MHN throughout | 7 (0.2) | 0.27 (0.03, 2.40) | 0.29 (0.08, 1.11) | 0.42 (0.08, 2.19) |  | 0.32 (0.04, 2.81) | 0.33 (0.09, 1.28) | 0.48 (0.09, 2.53) |  |
| **Atrial fibrillation** |  |  |  |  | 0.832 |  |  |  | 0.822 |
| MUOO throughout | 235 (10.7) | 1.0 (Reference) | 0.48 (0.36, 0.64) * | 0.33 (0.22, 0.49) * |  | 1.0 (Reference) | 0.48 (0.36, 0.64) * | 0.33 (0.22, 0.50) * |  |
| MHOO to MUOO | 67 (6.2) | 0.63 (0.38, 1.03) | 0.35 (0.24, 0.51) * | 0.17 (0.08, 0.36) * |  | 0.63 (0.38, 1.04) | 0.35 (0.24, 0.52) * | 0.17 (0.08, 0.36) * |  |
| MHOO throughout | 180 (5.2) | 0.67 (0.47, 0.94) * | 0.35 (0.26, 0.48) * | 0.15 (0.08, 0.26) * |  | 0.68 (0.48, 0.96) * | 0.36 (0.27, 0.49) * | 0.15 (0.08, 0.26) * |  |
| MHN to MHOO | 25 (4.5) | 0.66 (0.35, 1.25) | 0.28 (0.15, 0.52) * | 0.16 (0.05, 0.51) * |  | 0.68 (0.36, 1.29) | 0.28 (0.15, 0.54) * | 0.16 (0.05, 0.52) * |  |
| MHN throughout | 124 (3.6) | 0.53 (0.36, 0.77) * | 0.26 (0.19, 0.37) * | 0.09 (0.05, 0.18) * |  | 0.54 (0.37, 0.79) * | 0.27 (0.19, 0.37) * | 0.10 (0.05, 0.19) * |  |
| **Atrial fibrillation mortality** | |  |  |  | 0.009 |  |  |  | 0.024 |
| MUOO throughout | 12 (0.5) | 1.0 (Reference) | 1.32 (0.26, 6.64) | 1.54 (0.25, 9.59) |  | 1.0 (Reference) | 1.19 (0.23, 6.10) | 1.46 (0.23, 9.20) |  |
| MHOO to MUOO | 4 (0.4) | - | 2.06 (0.36, 11.77) | - |  | - | 2.06 (0.35, 11.98) | - |  |
| MHOO throughout | 5 (0.1) | 1.45 (0.19, 10.90) | 0.66 (0.11, 4.17) | - |  | 1.57 (0.21, 11.99) | 0.72 (0.11, 4.62) | - |  |
| MHN to MHOO | 1 (0.2) | 3.79 (0.33, 44.03) | - | - |  | 3.95 (0.34, 46.53) | - | - |  |
| MHN throughout | 3 (0.1) | - | 0.76 (0.12, 4.79) | - |  | - | 0.79 (0.12, 5.04) | - |  |

Note, BMI, body mass index; MH status was defined as < 3 abnormal components; MH, metabolically healthy; MHN, metabolically healthy normal weight; MHOW, metabolically healthy overweight; MHO, metabolically healthy obesity; MUN, metabolically unhealthy normal weight; MUOW, metabolically unhealthy overweight; MUO, metabolically unhealthy obesity; PRS, polygenic risk score; the PRSs presented are specifically used based on corresponding outcomes; Model 1 was adjusted for age, sex, race, Townsend Deprivation Index, annual household income, education attainment, 22 assessment centers, and the first 5 principal components of ancestry. Model 2 was further adjusted for family history of diabetes, family history of high blood pressure, and lifestyle factors including sleep duration, healthy diet, physical activity, smoking status, and alcohol intake frequency, based on Model 1; **^a^** likelihood tests was applied to test the significance of interaction term by comparing the model with and without the interaction term; **P* < 0.05.

**Table S18 Associations of** **metabolic status, BMI status, BMI-metabolic status, and PRSs with the risk of all-cause mortality stratified by potential risk factors**

| **Subgroup** | **Exposures** | **No** | **Case (%)** | **High PRS** | **Moderate PRS** | **Low PRS** | ***P* for interaction ^a^** |
| --- | --- | --- | --- | --- | --- | --- | --- |
| **Sex** |  |  |  |  |  |  |  |
|  | **Metabolic status** |  |  |  |  |  | 0.018 |
| **Male** | MU | 60 083 | 7761 (12.9) | 1.0 (Reference) | 0.96 (0.91, 1.01) | 0.86 (0.80, 0.93) * |  |
|  | MH | 113 832 | 8556 (7.5) | 0.82 (0.77, 0.88) * | 0.75 (0.71, 0.79) * | 0.68 (0.64, 0.73) * |  |
| **Female** | MU | 45 339 | 3855 (8.5) | 1.0 (Reference) | 1.02 (0.94, 1.10) | 1.04 (0.94, 1.15) |  |
|  | MH | 165 606 | 8175 (4.9) | 0.75 (0.69, 0.82) * | 0.71 (0.66, 0.76) * | 0.72 (0.66, 0.78) * |  |
| **Male** | **BMI status** |  |  |  |  |  | 0.004 |
|  | Obesity | 47 913 | 5535 (11.6) | 1.0 (Reference) | 0.97 (0.90, 1.03) | 0.81 (0.75, 0.89) * |  |
|  | Overweight | 98 184 | 8445 (8.6) | 0.79 (0.73, 0.85) * | 0.73 (0.69, 0.78) * | 0.66 (0.61, 0.71) * |  |
|  | Normal weight | 50 653 | 4329 (8.5) | 0.86 (0.79, 0.94) * | 0.76 (0.71, 0.82) * | 0.71 (0.65, 0.78) * |  |
| **Female** | Obesity | 55 776 | 4038 (7.2) | 1.0 (Reference) | 0.96 (0.89, 1.04) | 0.98 (0.89, 1.08) |  |
|  | Overweight | 89 537 | 4994 (5.6) | 0.83 (0.76, 0.91) * | 0.80 (0.74, 0.86) * | 0.74 (0.68, 0.82) * |  |
|  | Normal weight | 96 787 | 4542 (4.7) | 0.78 (0.71, 0.86) * | 0.73 (0.68, 0.79) * | 0.80 (0.73, 0.88) * |  |
|  | **BMI-metabolic status** |  |  |  |  |  | 0.054 |
| **Male** | MUO | 28 682 | 3704 (12.9) | 1.0 (Reference) | 0.96 (0.89, 1.04) | 0.82 (0.74, 0.91) * |  |
|  | MUOW | 25 720 | 3196 (12.4) | 0.84 (0.76, 0.93) * | 0.80 (0.74, 0.87) * | 0.76 (0.68, 0.85) * |  |
|  | MUN | 5475 | 804 (14.7) | 0.91 (0.77, 1.07) | 0.91 (0.81, 1.02) | 0.82 (0.68, 0.98) * |  |
|  | MHO | 13 651 | 1180 (8.6) | 0.81 (0.70, 0.95) * | 0.83 (0.76, 0.92) * | 0.71 (0.61, 0.82) * |  |
|  | MHOW | 60 727 | 4160 (6.9) | 0.71 (0.64, 0.78) * | 0.65 (0.60, 0.70) * | 0.58 (0.52, 0.63) * |  |
|  | MHN | 38 874 | 2970 (7.6) | 0.81 (0.72, 0.90) * | 0.68 (0.63, 0.74) * | 0.67 (0.60, 0.75) * |  |
| **Female** | MUO | 26 292 | 2241 (8.5) | 1.0 (Reference) | 0.98 (0.88, 1.08) | 0.99 (0.87, 1.13) |  |
|  | MUOW | 15 812 | 1308 (8.3) | 0.90 (0.78, 1.04) | 0.96 (0.86, 1.07) | 0.87 (0.74, 1.02) |  |
|  | MUN | 3124 | 278 (8.9) | 0.81 (0.62, 1.07) | 0.92 (0.76, 1.10) | 1.47 (1.13, 1.90) |  |
|  | MHO | 21 945 | 1294 (5.9) | 0.80 (0.69, 0.93) * | 0.74 (0.66, 0.83) * | 0.77 (0.66, 0.89) * |  |
|  | MHOW | 61 523 | 3040 (4.9) | 0.70 (0.62, 0.79) * | 0.65 (0.59, 0.72) * | 0.62 (0.55, 0.70) * |  |
|  | MHN | 80 337 | 3641 (4.5) | 0.69 (0.62, 0.77) * | 0.64 (0.58, 0.71) * | 0.69 (0.62, 0.78) * |  |
| **Ethnic** |  |  |  |  |  |  |  |
| **White** | **Metabolic status** |  |  |  |  |  |  |
|  | MU | 98 990 | 11 095 (11.2) | 1.0 (Reference) | 0.97 (0.92, 1.01) | 0.89 (0.84, 0.94) * |  |
|  | MH | 263 779 | 16 125 (6.1) | 0.81 (0.77, 0.86) * | 0.75 (0.72, 0.78) * | 0.72 (0.68, 0.75) * |  |
| **White** | **BMI status** |  |  |  |  |  |  |
|  | Obesity | 96 858 | 9158 (9.5) | 1.0 (Reference) | 0.96 (0.91, 1.01) | 0.87 (0.81, 0.93) * |  |
|  | Overweight | 177 086 | 12 900 (7.3) | 0.80 (0.76, 0.85) * | 0.74 (0.71, 0.78) * | 0.68 (0.64, 0.72) * |  |
|  | Normal weight | 139 761 | 8566 (6.1) | 0.83 (0.78, 0.89) * | 0.76 (0.72, 0.81) * | 0.77 (0.72, 0.82) * |  |
| **White** | **BMI-Metabolic status** |  |  |  |  |  |  |
|  | MUO | 51 975 | 5706 (11.0) | 1.0 (Reference) | 0.95 (0.89, 1.02) | 0.85 (0.78, 0.93) * |  |
|  | MUOW | 38 962 | 4297 (11.0) | 0.84 (0.77, 0.91) * | 0.81 (0.76, 0.87) * | 0.76 (0.69, 0.83) * |  |
|  | MUN | 7813 | 1018 (13.0) | 0.86 (0.74, 0.99) * | 0.89 (0.81, 0.99) * | 0.91 (0.78, 1.06) |  |
|  | MHO | 32 711 | 2352 (7.2) | 0.81 (0.73, 0.91) * | 0.81 (0.75, 0.87) * | 0.75 (0.67, 0.84) * |  |
|  | MHOW | 115 560 | 7021 (6.1) | 0.71 (0.65, 0.76) * | 0.65 (0.61, 0.69) * | 0.59 (0.54, 0.63) * |  |
|  | MHN | 113 343 | 6425 (5.7) | 0.77 (0.71, 0.83) * | 0.68 (0.64, 0.73) * | 0.70 (0.65, 0.76) * |  |
| **Follow up** |  |  |  |  |  |  |  |
| **>2 years** | **Metabolic status** |  |  |  |  |  |  |
|  | MU | 104 942 | 11 136 (10.6) | 1.0 (Reference) | 0.97 (0.92, 1.01) | 0.90 (0.85, 0.96) * |  |
|  | MH | 278 732 | 16 025 (5.7) | 0.80 (0.76, 0.85) * | 0.74 (0.71, 0.78) * | 0.72 (0.68, 0.76) * |  |
| **>2 year** | **BMI status** |  |  |  |  |  |  |
|  | Obesity | 103 314 | 9198 (8.9) | 1.0 (Reference) | 0.96 (0.91, 1.01) | 0.87 (0.81, 0.93) * |  |
|  | Overweight | 187 172 | 12 890 (6.9) | 0.79 (0.75, 0.84) * | 0.74 (0.70, 0.78) * | 0.68 (0.64, 0.72) * |  |
|  | Normal weight | 147 032 | 8463 (5.8) | 0.81 (0.76, 0.87) * | 0.75 (0.71, 0.79) * | 0.76 (0.72, 0.81) * |  |
| **>2 year** | **BMI-metabolic status** |  |  |  |  |  |  |
|  | MUO | 54 749 | 5720 (10.4) | 1.0 (Reference) | 0.95 (0.89, 1.02) | 0.86 (0.79, 0.93) * |  |
|  | MUOW | 41 341 | 4313 (10.4) | 0.83 (0.76, 0.90) * | 0.81 (0.76, 0.87) * | 0.76 (0.69, 0.83) * |  |
|  | MUN | 8543 | 1026 (12.0) | 0.84 (0.73, 0.97) * | 0.86 (0.78, 0.95) * | 0.92 (0.79, 1.08) |  |
|  | MHO | 35 491 | 2369 (6.7) | 0.80 (0.72, 0.89) * | 0.79 (0.74, 0.86) * | 0.74 (0.66, 0.82) * |  |
|  | MHOW | 121 967 | 7017 (5.8) | 0.70 (0.65, 0.76) * | 0.64 (0.60, 0.68) * | 0.59 (0.55, 0.64) * |  |
|  | MHN | 118 915 | 6315 (5.3) | 0.75 (0.69, 0.81) * | 0.67 (0.63, 0.71) * | 0.69 (0.64, 0.75) * |  |

Note, BMI, body mass index; MH status was defined as < 3 abnormal components; MH, metabolically healthy; MU, metabolically unhealthy; MHN, metabolically healthy normal weight; MHOW, metabolically healthy overweight; MHO, metabolically healthy obesity; MUN, metabolically unhealthy normal weight; MUOW, metabolically unhealthy overweight; MUO, metabolically unhealthy obesity; the PRSs presented are specifically used based on corresponding outcomes; each model was adjusted for age, sex, race, Townsend Deprivation Index, annual household income, education attainment, and 22 assessment centers, the first 5 principal components of ancestry, family history of diabetes, family history of high blood pressure, and lifestyle factors including sleep duration, healthy diet, physical activity, smoking status, and alcohol intake frequency; the subgroup analysis of females was additionally adjusted for pregnancy history and menopausal status; **^a^** likelihood tests were applied to test the significance of the interaction term by comparing the model with and without the interaction term; *, *P* < 0.05

**Table S19 Associations of metabolic status, BMI status, BMI-metabolic status, and PRSs with the risk of CVD morbidity stratified by potential risk factors**

| **Subgroup** | **Exposures** | **No** | **Case (%)** | **High PRS** | **Moderate PRS** | **Low PRS** | ***P* for interaction ^a^** |
| --- | --- | --- | --- | --- | --- | --- | --- |
| **Sex** |  |  |  |  |  |  |  |
|  | **Metabolic status** |  |  |  |  |  | < 0.001 |
| **Male** | MU | 62 356 | 16 913 (27.0) | 1.0 (Reference) | 0.75 (0.72, 0.78) * | 0.58 (0.56, 0.61) * |  |
|  | MH | 117 065 | 18 052 (15.0) | 0.70 (0.67, 0.74) * | 0.50 (0.49, 0.52) * | 0.39 (0.37, 0.41) * |  |
| **Female** | MU | 47 795 | 8210 (17.2) | 1.0 (Reference) | 0.79 (0.75, 0.83) * | 0.63 (0.59, 0.68) * |  |
|  | MH | 172 143 | 16 054 (9.3) | 0.70 (0.66, 0.73) * | 0.54 (0.51, 0.56) * | 0.46 (0.43, 0.48) * |  |
|  | **BMI status** |  |  |  |  |  | < 0.001 |
| **Male** | Obesity | 49497 | 12526 (25.3) | 1.0 (Reference) | 0.76 (0.73, 0.79) * | 0.61 (0.57, 0.64) * |  |
|  | Overweight | 101203 | 18952 (18.7) | 0.76 (0.73, 0.80) * | 0.54 (0.52, 0.56) * | 0.40 (0.38, 0.42) * |  |
|  | Normal weight | 52244 | 8014 (15.3) | 0.64 (0.60, 0.68) * | 0.46 (0.44, 0.48) * | 0.34 (0.32, 0.37) * |  |
| **Female** | Obesity | 58441 | 9259 (15.8) | 1.0 (Reference) | 0.80 (0.76, 0.84) * | 0.66 (0.61, 0.70) * |  |
|  | Overweight | 93532 | 10500 (11.2) | 0.74 (0.70, 0.78) * | 0.56 (0.54, 0.59) * | 0.45 (0.42, 0.48) * |  |
|  | Normal weight | 100631 | 8009 (8.0) | 0.61 (0.57, 0.65) * | 0.46 (0.44, 0.48) * | 0.41 (0.38, 0.44) * |  |
|  | **BMI-metabolic status** |  |  |  |  |  | < 0.001 |
| **Male** | MUO | 29 693 | 8352 (28.1) | 1.0 (Reference) | 0.76 (0.72, 0.79) * | 0.61 (0.57, 0.65) * |  |
|  | MUOW | 26 742 | 6997 (26.2) | 0.82 (0.77, 0.87) * | 0.61 (0.58, 0.65) * | 0.45 (0.42, 0.49) * |  |
|  | MUN | 5707 | 1489 (26.1) | 0.78 (0.70, 0.87) * | 0.56 (0.52, 0.61) * | 0.42 (0.37, 0.49) * |  |
|  | MHO | 14 030 | 2715 (19.4) | 0.78 (0.72, 0.86) * | 0.60 (0.57, 0.64) * | 0.49 (0.44, 0.54) * |  |
|  | MHOW | 62 379 | 9712 (15.6) | 0.65 (0.61, 0.68) * | 0.45 (0.43, 0.47) * | 0.34 (0.32, 0.37) * |  |
|  | MHN | 40 049 | 5506 (13.7) | 0.55 (0.52, 0.60) * | 0.40 (0.38, 0.42) * | 0.30 (0.28, 0.33) * |  |
| **Female** | MUO | 27 640 | 4979 (18.0) | 1.0 (Reference) | 0.80 (0.74, 0.85) * | 0.61 (0.56, 0.67) * |  |
|  | MUOW | 16 735 | 2708 (16.2) | 0.81 (0.74, 0.89) * | 0.63 (0.58, 0.67) * | 0.51 (0.45, 0.57) * |  |
|  | MUN | 3300 | 497 (15.1) | 0.73 (0.61, 0.87) * | 0.52 (0.46, 0.59) * | 0.60 (0.48, 0.74) * |  |
|  | MHO | 22 843 | 3044 (13.3) | 0.81 (0.74, 0.89) * | 0.67 (0.62, 0.72) * | 0.58 (0.53, 0.64) * |  |
|  | MHOW | 63 991 | 6409 (10.0) | 0.63 (0.59, 0.68) * | 0.49 (0.46, 0.52) * | 0.40 (0.36, 0.43) * |  |
|  | MHN | 83 422 | 6409 (7.7) | 0.55 (0.51, 0.59) * | 0.42 (0.39, 0.44) * | 0.37 (0.34, 0.40) * |  |
| **Ethnic** |  |  |  |  |  |  |  |
| **White** | **Metabolic status** |  |  |  |  |  |  |
|  | MU | 273 197 | 32 635 (11.9) | 1.0 (Reference) | 0.77 (0.74, 0.79) * | 0.60 (0.57, 0.62) * |  |
|  | MH | 103 538 | 23 759 (22.9) | 0.70 (0.68, 0.73) * | 0.52 (0.50, 0.53) * | 0.42 (0.40, 0.43) * |  |
| **White** | **BMI status** |  |  |  |  |  |  |
|  | Obesity | 100 913 | 20 662 (20.5) | 1.0 (Reference) | 0.78 (0.75, 0.80) * | 0.62 (0.59, 0.65) * |  |
|  | Overweight | 183 857 | 28 065 (15.3) | 0.76 (0.73, 0.78) * | 0.55 (0.53, 0.57) * | 0.41 (0.40, 0.43) * |  |
|  | Normal weight | 145 009 | 15 296 (10.5) | 0.61 (0.59, 0.64) * | 0.45 (0.44, 0.47) * | 0.37 (0.36, 0.39) * |  |
| **White** | **BMI-metabolic status** |  |  |  |  |  |  |
|  | MUO | 54 252 | 12 707 (23.4) | 1.0 (Reference) | 0.77 (0.74, 0.80) * | 0.60 (0.57, 0.64) * |  |
|  | MUOW | 40 833 | 9149 (22.4) | 0.81 (0.77, 0.85) * | 0.61 (0.59, 0.64) * | 0.46 (0.43, 0.49) * |  |
|  | MUN | 8200 | 1821 (22.2) | 0.75 (0.68, 0.82) * | 0.55 (0.52, 0.59) * | 0.47 (0.41, 0.53) * |  |
|  | MHO | 33 910 | 5418 (16.0) | 0.80 (0.75, 0.85) * | 0.63 (0.60, 0.67) * | 0.53 (0.49, 0.57) * |  |
|  | MHOW | 119 549 | 15 468 (12.9) | 0.65 (0.62, 0.68) * | 0.46 (0.45, 0.48) * | 0.36 (0.34, 0.38) * |  |
|  | MHN | 117 465 | 11 452 (9.7) | 0.54 (0.52, 0.57) * | 0.40 (0.39, 0.42) * | 0.33 (0.31, 0.35) * |  |
| **Follow up** |  |  |  |  |  |  |  |
| **> 2 years** | **Metabolic status** |  |  |  |  |  |  |
|  | MU | 106 609 | 22 086 (20.7) | 1.0 (Reference) | 0.77 (0.75, 0.80) * | 0.60 (0.58, 0.63) * |  |
|  | MH | 285 166 | 30 910 (10.8) | 0.71 (0.68, 0.73) * | 0.53 (0.51, 0.54) * | 0.42 (0.41, 0.44) * |  |
| **> 2 years** | **BMI status** |  |  |  |  |  |  |
|  | Obesity | 105 102 | 19 374 (18.4) | 1.0 (Reference) | 0.79 (0.77, 0.82) * | 0.63 (0.60, 0.66) * |  |
|  | Overweight | 191 006 | 26 331 (13.8) | 0.76 (0.73, 0.79) * | 0.55 (0.54, 0.57) * | 0.43 (0.41, 0.44) * * |  |
|  | Normal weight | 150 891 | 14 539 (9.6) | 0.62 (0.60, 0.65) * | 0.46 (0.45, 0.48) * | 0.38 (0.36, 0.40) |  |
| **> 2 years** | **BMI-metabolic status** |  |  |  |  |  |  |
|  | MUO | 55 497 | 11 751 (21.2) | 1.0 (Reference) | 0.78 (0.75, 0.82) * | 0.61 (0.58, 0.65) * |  |
|  | MUOW | 42 100 | 8514 (20.2) | 0.82 (0.77, 0.86) * | 0.62 (0.59, 0.65) * | 0.47 (0.44, 0.50) * |  |
|  | MUN | 8700 | 1737 (20.0) | 0.77 (0.70, 0.85) * | 0.54 (0.51, 0.58) * | 0.48 (0.42, 0.54) * |  |
|  | MHO | 36 193 | 5193 (14.3) | 0.80 (0.75, 0.86) * | 0.65 (0.62, 0.69) * | 0.54 (0.50, 0.58) * |  |
|  | MHOW | 124 493 | 14 584 (11.7) | 0.65 (0.62, 0.69) * | 0.47 (0.45, 0.49) * | 0.37 (0.35, 0.39) * |  |
|  | MHN | 122 054 | 10 865 (8.9) | 0.55 (0.52, 0.58) * | 0.41 (0.39, 0.43) * | 0.34 (0.32, 0.36) * |  |

Note, BMI, body mass index; MH status was defined as < 3 abnormal components; MH, metabolically healthy; MU, metabolically unhealthy; MHN, metabolically healthy normal weight; MHOW, metabolically healthy overweight; MHO, metabolically healthy obesity; MUN, metabolically unhealthy normal weight; MUOW, metabolically unhealthy overweight; MUO, metabolically unhealthy obesity; the PRSs presented are specifically used based on corresponding outcomes; each model was adjusted for age, sex, race, Townsend Deprivation Index, annual household income, education attainment, and 22 assessment centers, the first 5 principal components of ancestry, family history of diabetes, family history of high blood pressure, and lifestyle factors including sleep duration, healthy diet, physical activity, smoking status, and alcohol intake frequency; the subgroup analysis of females was additionally adjusted for pregnancy history and menopausal status; **^a^** likelihood tests were applied to test the significance of the interaction term by comparing the model with and without the interaction term; *, *P* < 0.05

**Table S20 Associations of metabolic status, BMI status, BMI-metabolic status, and PRSs with the risk of CVD mortality stratified by potential risk factors**

| **Subgroup** | **Exposures** | **No** | **Case (%)** | **High PRS** | **Moderate PRS** | **Low PRS** | ***P* for interaction ^a^** |
| --- | --- | --- | --- | --- | --- | --- | --- |
| **Sex** |  |  |  |  |  |  |  |
|  | **Metabolic status** |  |  |  |  |  | 0.156 |
| **Male** | MU | 62 356 | 2615 (4.2) | 1.0 (Reference) | 0.74 (0.68, 0.81) * | 0.57 (0.50, 0.65) * |  |
|  | MH | 117 065 | 2154 (1.8) | 0.70 (0.62, 0.78) * | 0.45 (0.41, 0.50) * | 0.33 (0.29, 0.38) * |  |
| **Female** | MU | 47 795 | 965 (2.0) | 1.0 (Reference) | 0.81 (0.70, 0.94) * | 0.70 (0.57, 0.86) * |  |
|  | MH | 172 143 | 1486 (0.9) | 0.74 (0.63, 0.87) * | 0.48 (0.42, 0.56) * | 0.43 (0.36, 0.51) * |  |
|  | BMI status |  |  |  |  |  |  |
|  | **BMI status** |  |  |  |  |  | 0.313 |
| **Male** | Obesity | 49497 | 1860 (3.8) | 1.0 (Reference) | 0.71 (0.63, 0.78) * | 0.50 (0.43, 0.58) * |  |
|  | Overweight | 101203 | 2400 (2.4) | 0.67 (0.60, 0.76) * | 0.47 (0.42, 0.52) * | 0.34 (0.29, 0.39) * |  |
|  | Normal weight | 52244 | 1109 (2.1) | 0.67 (0.58, 0.78) * | 0.43 (0.38, 0.49) * | 0.34 (0.28, 0.40) * |  |
| **Female** | Obesity | 58441 | 976 (1.7) | 1.0 (Reference) | 0.70 (0.60, 0.81) * | 0.67 (0.55, 0.81) * |  |
|  | Overweight | 93532 | 968 (1.0) | 0.66 (0.56, 0.79) * | 0.49 (0.42, 0.57) * | 0.36 (0.29, 0.45) * |  |
|  | Normal weight | 100631 | 820 (0.8) | 0.72 (0.60, 0.86) * | 0.46 (0.40, 0.54) * | 0.41 (0.33, 0.50) * |  |
|  | **BMI-metabolic status** |  |  |  |  |  | 0.118 |
| **Male** | MUO | 29 693 | 694 (2.3) | 1.0 (Reference) | 0.71 (0.62, 0.80) * | 0.54 (0.46, 0.65) * |  |
|  | MUOW | 26 742 | 1115 (4.2) | 0.68 (0.58, 0.80) * | 0.54 (0.48, 0.62) * | 0.41 (0.33, 0.49) * |  |
|  | MUN | 5707 | 314 (5.5) | 0.82 (0.64, 1.05) | 0.61 (0.50, 0.74) * | 0.50 (0.36, 0.69) * |  |
|  | MHO | 14 030 | 269 (1.9) | 0.63 (0.49, 0.82) * | 0.51 (0.43, 0.61) * | 0.29 (0.21, 0.40) * |  |
|  | MHOW | 62 379 | 1006 (1.6) | 0.58 (0.50, 0.68) * | 0.37 (0.32, 0.42) * | 0.28 (0.23, 0.33) * |  |
|  | MHN | 40 049 | 1324 (3.3) | 0.57 (0.47, 0.68) * | 0.34 (0.30, 0.40) * | 0.28 (0.23, 0.35) * |  |
| **Female** | MUO | 27 640 | 577 (2.1) | 1.0 (Reference) | 0.78 (0.64, 0.94) * | 0.66 (0.51, 0.87) * |  |
|  | MUOW | 16 735 | 316 (1.9) | 0.78 (0.60, 1.02) | 0.64 (0.52, 0.79) * | 0.54 (0.39, 0.75) * |  |
|  | MUN | 3300 | 68 (2.1) | 0.68 (0.40, 1.16) | 0.65 (0.46, 0.92) * | 0.77 (0.44, 1.36) |  |
|  | MHO | 22 843 | 277 (1.2) | 0.82 (0.62, 1.07) | 0.49 (0.39, 0.61) * | 0.63 (0.47, 0.85) * |  |
|  | MHOW | 63 991 | 528 (0.8) | 0.56 (0.44, 0.70) * | 0.39 (0.32, 0.48) * | 0.29 (0.22, 0.38) * |  |
|  | MHN | 83 422 | 634 (0.8) | 0.67 (0.54, 0.84) * | 0.40 (0.33, 0.49) * | 0.37 (0.29, 0.47) * |  |
| **Ethnic** |  |  |  |  |  |  |  |
| **White** | **Metabolic status** |  |  |  |  |  |  |
|  | MU | 103 538 | 3393 (3.3) | 1.0 (Reference) | 0.76 (0.70, 0.82) * | 0.60 (0.53, 0.67) * |  |
|  | MH | 273 197 | 3500 (1.3) | 0.72 (0.65, 0.79) * | 0.46 (0.43, 0.50) * | 0.37 (0.33, 0.41) * |  |
| **White** | **BMI status** |  |  |  |  |  |  |
|  | Obesity | 100 913 | 2705 (2.7) | 1.0 (Reference) | 0.71 (0.65, 0.78) * | 0.55 (0.49, 0.62) * |  |
|  | Overweight | 183 857 | 3206 (1.7) | 0.67 (0.61, 0.74) * | 0.48 (0.44, 0.52) * | 0.34 (0.30, 0.38) * |  |
|  | Normal weight | 145 009 | 1851 (1.3) | 0.70 (0.62, 0.79) * | 0.45 (0.41, 0.49) * | 0.37 (0.32, 0.42) * |  |
| **White** | **BMI-Metabolic status** |  |  |  |  |  |  |
|  | MUO | 54 252 | 1816 (3.3) | 1.0 (Reference) | 0.74 (0.66, 0.82) | 0.57 (0.49, 0.67) |  |
|  | MUOW | 40 833 | 1249 (3.1) | 0.72 (0.62, 0.83) * | 0.57 (0.50, 0.63) * | 0.43 (0.36, 0.52) * |  |
|  | MUN | 8 200 | 313 (3.8) | 0.80 (0.63, 1.01) | 0.64 (0.54, 0.76) * | 0.57 (0.42, 0.76) * |  |
|  | MHO | 33 910 | 565 (1.7) | 0.72 (0.60, 0.87) * | 0.51 (0.44, 0.59) * | 0.44 (0.35, 0.54) * |  |
|  | MHOW | 119 549 | 1573 (1.3) | 0.58 (0.50, 0.66) * | 0.38 (0.34, 0.42) * | 0.28 (0.24, 0.33) * |  |
|  | MHN | 117 465 | 1286 (1.1) | 0.62 (0.54, 0.71) * | 0.37 (0.33, 0.42) * | 0.32 (0.27, 0.38) * |  |
| **Follow up** |  |  |  |  |  |  |  |
| **>2 year** | **Metabolic status** |  |  |  |  |  |  |
|  | MU | 109 521 | 3418 (3.1) | 1.0 (Reference) | 0.76 (0.71, 0.83) * | 0.61 (0.55, 0.68) * |  |
|  | MH | 288 226 | 3478 (1.2) | 0.70 (0.64, 0.77) * | 0.46 (0.43, 0.50) * | 0.37 (0.33, 0.41) * |  |
| **>2 year** | **BMI status** |  |  |  |  |  |  |
|  | Obesity | 107 430 | 2724 (2.5) | 1.0 (Reference) | 0.71 (0.65, 0.77) * | 0.54 (0.48, 0.61) * |  |
|  | Overweight | 194 001 | 3204 (1.7) | 0.66 (0.59, 0.73) * | 0.47 (0.43, 0.51) * | 0.34 (0.30, 0.38) * |  |
|  | Normal weight | 152 299 | 1845 (1.2) | 0.66 (0.59, 0.75) * | 0.44 (0.40, 0.48) * | 0.37 (0.32, 0.42) * |  |
| **>2 year** | **BMI-Metabolic status** |  |  |  |  |  |  |
|  | MUO | 57 023 | 1823 (3.2) | 1.0 (Reference) | 0.74 (0.66, 0.82) * | 0.57 (0.49, 0.67) * |  |
|  | MUOW | 43 240 | 1258 (2.9) | 0.70 (0.60, 0.80) * | 0.56 (0.50, 0.63) * | 0.44 (0.37, 0.53) * |  |
|  | MUN | 8933 | 318 (3.6) | 0.79 (0.63, 1.00) | 0.60 (0.51, 0.72) * | 0.55 (0.41, 0.74) * |  |
|  | MHO | 36 740 | 569 (1.5) | 0.71 (0.59, 0.85) * | 0.50 (0.44, 0.58) * | 0.42 (0.34, 0.52) * |  |
|  | MHOW | 125 971 | 1564 (1.2) | 0.56 (0.49, 0.64) * | 0.37 (0.34, 0.42) * | 0.28 (0.24, 0.32) * |  |
|  | MHN | 123 051 | 1275 (1.0) | 0.58 (0.51, 0.67) * | 0.37 (0.33, 0.41) * | 0.32 (0.27, 0.38) * |  |

Note, BMI, body mass index; MH status was defined as < 3 abnormal components; MH, metabolically healthy; MU, metabolically unhealthy; MHN, metabolically healthy normal weight; MHOW, metabolically healthy overweight; MHO, metabolically healthy obesity; MUN, metabolically unhealthy normal weight; MUOW, metabolically unhealthy overweight; MUO, metabolically unhealthy obesity; the PRSs presented are specifically used based on corresponding outcomes; each model was adjusted for age, sex, race, Townsend Deprivation Index, annual household income, education attainment, and 22 assessment centers, the first 5 principal components of ancestry, family history of diabetes, family history of high blood pressure, and lifestyle factors including sleep duration, healthy diet, physical activity, smoking status, and alcohol intake frequency; the subgroup analysis of females was additionally adjusted for pregnancy history and menopausal status; **^a^** likelihood tests were applied to test the significance of the interaction term by comparing the model with and without the interaction term; *, *P* < 0.05

**Table S21 Associations of baseline exposures (metabolic status, BMI status, BMI-metabolic status), PRSs and CVD morbidity, including participants who participated in the second survey and who died between baseline and the second survey**

|  | **Exposures** | **Model** | **Case (%)** | **High PRS** | **Moderate PRS** | **Low PRS** | ***P* ^a^** |
| --- | --- | --- | --- | --- | --- | --- | --- |
| **CVD morbidity** | **Metabolic status** | **Model A** |  |  |  |  | 0.271 |
|  | MU |  | 408 (19.5) | 1.0 (Reference) | 0.72 (0.58, 0.91) * | 0.48 (0.34, 0.67) * |  |
|  | MH |  | 889 (9.7) | 0.64 (0.50, 0.81) * | 0.49 (0.40, 0.60) * | 0.38 (0.30, 0.49) * |  |
|  | **Metabolic status** | **Model B** |  |  |  |  |  |
|  | MU |  | 900 (22.2) | 1.0 (Reference) | 0.79 (0.68, 0.93) * | 0.66 (0.53, 0.82) * |  |
|  | MH |  | 1418 (11.5) | 0.67 (0.56, 0.80) * | 0.49 (0.42, 0.57) * | 0.41 (0.34, 0.49) * |  |
|  | **BMI status** | **Model A** |  |  |  |  | 0.733 |
|  | Obesity |  | 446 (15.6) | 1.0 (Reference) | 0.77 (0.61, 0.96) * | 0.71 (0.52, 0.96) * |  |
|  | Overweight |  | 848 (12.5) | 0.85 (0.67, 1.08) | 0.59 (0.47, 0.73) * | 0.43 (0.33, 0.55) * |  |
|  | Normal weight |  | 627 (9.0) | 0.56 (0.43, 0.74) * | 0.55 (0.44, 0.68) * | 0.34 (0.26, 0.45) * |  |
|  | **BMI status** | **Model B** |  |  |  |  |  |
|  | Obesity |  | 806 (18.0) | 1.0 (Reference) | 0.75 (0.63, 0.89) * | 0.71 (0.57, 0.89) * |  |
|  | Overweight |  | 1314 (14.3) | 0.84 (0.70, 1.01) | 0.60 (0.51, 0.70) * | 0.47 (0.39, 0.57) * |  |
|  | Normal weight |  | 931 (10.7) | 0.62 (0.51, 0.76) * | 0.55 (0.46, 0.64) * | 0.37 (0.29, 0.45) * |  |
|  | **BMI-metabolic status** | **Model A** |  |  |  |  | 0.615 |
|  | MUO |  | 203 (19.6) | 1.0 (Reference) | 0.69 (0.50, 0.96) * | 0.56 (0.35, 0.89) * |  |
|  | MUOW |  | 161 (19.8) | 0.81 (0.54, 1.21) | 0.66 (0.47, 0.92) * | 0.33 (0.19, 0.57) * |  |
|  | MUN |  | - | - | - | - |  |
|  | MHO |  | 96 (10.3) | 0.68 (0.41, 1.15) | 0.47 (0.33, 0.69) * | 0.41 (0.24, 0.70) * |  |
|  | MHOW |  | 389 (11.3) | 0.61 (0.43, 0.86) * | 0.47 (0.35, 0.64) * | 0.38 (0.27, 0.55) * |  |
|  | MHN |  | 313 (7.9) | 0.48 (0.33, 0.70) * | 0.41 (0.30, 0.56) * | 0.24 (0.16, 0.36) * |  |
|  | **BMI-metabolic status** | **Model B** |  |  |  |  |  |
|  | MUO |  | 439 (21.9) | 1.0 (Reference) | 0.75 (0.60, 0.94) * | 0.71 (0.52, 0.96) * |  |
|  | MUOW |  | 355 (22.4) | 0.89 (0.67, 1.18) | 0.74 (0.59, 0.93) * | 0.51 (0.36, 0.72) * |  |
|  | MUN |  | 55 (27.5) | 1.03 (0.56, 1.88) | 1.43 (0.96, 2.14) | 1.87 (0.97, 3.61) |  |
|  | MHO |  | 177 (13.0) | 0.78 (0.54, 1.12) | 0.49 (0.38, 0.65) * | 0.44 (0.30, 0.65) * |  |
|  | MHOW |  | 610 (12.8) | 0.64 (0.50, 0.83) * | 0.48 (0.39, 0.60) * | 0.43 (0.33, 0.56) * |  |
|  | MHN |  | 524 (9.9) | 0.58 (0.45, 0.76) * | 0.43 (0.34, 0.53) * | 0.30 (0.23, 0.40) * |  |

Note, BMI, body mass index; MH status was defined as < 3 abnormal components; MH, metabolically healthy; MU, metabolically unhealthy; MHN, metabolically healthy normal weight; MHOW, metabolically healthy overweight; MHO, metabolically healthy obesity; MUN, metabolically unhealthy normal weight; MUOW, metabolically unhealthy overweight; MUO, metabolically unhealthy obesity; the PRSs presented are specifically used based on corresponding outcomes; each model was adjusted for age, sex, race, Townsend Deprivation Index, annual household income, education attainment, 22 assessment centers, the first 5 principal components of ancestry, family history of diabetes, family history of high blood pressure, and lifestyle factors including sleep duration, healthy diet, physical activity, smoking status, and alcohol intake frequency; Model A included participants who participated in the second survey, Model B included participants who participated in the second survey and who died between baseline and the second survey; **^a^** Wald tests were applied to test the significance of the coefficients of baseline exposures between Model A and Model B; *, *P* < 0.05

**Table S22 Associations of lifestyles and PRSs with all-cause mortality and cardiovascular outcomes**

| **Outcomes** | **Exposures** | **Case (%)** | **Model 1** | | | | **Model 2** | | | |
| --- | --- | --- | --- | --- | --- | --- | --- | --- | --- | --- |
|  |  |  | **High PRS** | **Moderate PRS** | **Low PRS** | ***P* for interaction ^a^** | **High PRS** | **Moderate PRS** | **Low PRS** | ***P* for interaction ^a^** |
| **All-cause mortality** | **Lifestyle factors** |  |  |  |  | 0.286 |  |  |  | 0.341 |
|  | Poor | 3477 (13.9) | 1.0 (Reference) | 0.96 (0.89, 1.05) | 0.83 (0.75, 0.93) * |  | 1.0 (Reference) | 0.97 (0.89, 1.05) | 0.84 (0.75, 0.94) * |  |
|  | Intermediate | 7881 (9.5) | 0.66 (0.61, 0.72) * | 0.63 (0.59, 0.68) * | 0.58 (0.53, 0.63) * |  | 0.73 (0.66, 0.79) * | 0.69 (0.64, 0.75) * | 0.64 (0.58, 0.70) * |  |
|  | Ideal | 21127 (6.3) | 0.46 (0.43, 0.50) * | 0.43 (0.40, 0.46) | 0.41 (0.37, 0.44) * |  | 0.53 (0.49, 0.58) * | 0.49 (0.45, 0.53) * | 0.46 (0.43, 0.51) * |  |
| **CVD morbidity** | **Lifestyle factors** |  |  |  |  | 0.104 |  |  |  | 0.091 |
|  | Poor | 5556 (21.3) | 1.0 (Reference) | 0.80 (0.75, 0.85) * | 0.63 (0.58, 0.69) * |  | 1.0 (Reference) | 0.80 (0.75, 0.85) * | 0.64 (0.59, 0.70) * |  |
|  | Intermediate | 15457 (17.9) | 0.85 (0.79, 0.90) * | 0.62 (0.58, 0.65) * | 0.49 (0.46, 0.52) * |  | 0.82 (0.77, 0.88) * | 0.61 (0.57, 0.64) * | 0.48 (0.45, 0.52) * |  |
|  | Ideal | 46877 (13.5) | 0.62 (0.59, 0.66) * | 0.46 (0.43, 0.49) * | 0.36 (0.34, 0.38) * |  | 0.58 (0.54, 0.62) * | 0.43 (0.40, 0.46) * | 0.33 (0.31, 0.36) * |  |
| **CVD mortality** | **Lifestyle factors** |  |  |  |  | 0.342 |  |  |  | 0.275 |
|  | Poor | 1006 (3.9) | 1.0 (Reference) | 0.78 (0.68, 0.91) * | 0.56 (0.46, 0.69) * |  | 1.0 (Reference) | 0.79 (0.68, 0.91) * | 0.56 (0.46, 0.69) * |  |
|  | Intermediate | 2213 (2.6) | 0.73 (0.63, 0.85) * | 0.49 (0.43, 0.56) * | 0.39 (0.33, 0.46) * |  | 0.77 (0.66, 0.89) * | 0.52 (0.45, 0.59) * | 0.42 (0.36, 0.50) * |  |
|  | Ideal | 5049 (1.5) | 0.44 (0.39, 0.50) * | 0.30 (0.26, 0.34) * | 0.22 (0.19, 0.26) * |  | 0.47 (0.40, 0.54) * | 0.32 (0.28, 0.37) * | 0.24 (0.20, 0.28) * |  |
| **Coronary disease** | **Lifestyle factors** |  |  |  |  | < 0.001 |  |  |  | < 0.001 |
|  | Poor | 3536 (13.3) | 1.0 (Reference) | 0.72 (0.67, 0.78) * | 0.49 (0.44, 0.55) * |  | 1.0 (Reference) | 0.72 (0.67, 0.78) * | 0.49 (0.44, 0.55) * |  |
|  | Intermediate | 9430 (10.7) | 0.83 (0.77, 0.90) * | 0.56 (0.52, 0.60) * | 0.37 (0.34, 0.41) * |  | 0.83 (0.77, 0.90) * | 0.56 (0.52, 0.60) * | 0.38 (0.35, 0.41) * |  |
|  | Ideal | 26840 (7.6) | 0.65 (0.60, 0.69) * | 0.40 (0.37, 0.42) * | 0.25 (0.24, 0.27) * |  | 0.64 (0.59, 0.69) * | 0.39 (0.36, 0.42) * | 0.25 (0.23, 0.27) * |  |
| **Coronary disease mortality** | **Lifestyle factors** |  |  |  |  | 0.149 |  |  |  | 0.157 |
|  | Poor | 674 (2.5) | 1.0 (Reference) | 0.70 (0.59, 0.84) * | 0.40 (0.31, 0.52) * |  | 1.0 (Reference) | 0.70 (0.58, 0.83) * | 0.40 (0.30, 0.52) * |  |
|  | Intermediate | 1377 (1.6) | 0.68 (0.57, 0.81) * | 0.44 (0.37, 0.51) * | 0.29 (0.24, 0.36) * |  | 0.71 (0.59, 0.85) * | 0.46 (0.39, 0.54) * | 0.31 (0.25, 0.38) * |  |
|  | Ideal | 2921 (0.8) | 0.43 (0.37, 0.51) * | 0.25 (0.22, 0.30) * | 0.15 (0.12, 0.18) * |  | 0.46 (0.38, 0.55) * | 0.27 (0.23, 0.32) * | 0.16 (0.13, 0.19) * |  |
| **Myocardial infarction** | **Lifestyle factors** |  |  |  |  | 0.062 |  |  |  | 0.071 |
|  | Poor | 1968 (7.1) | 1.0 (Reference) | 0.79 (0.71, 0.88) * | 0.56 (0.48, 0.65) * |  | 1.0 (Reference) | 0.79 (0.71, 0.88) * | 0.56 (0.48, 0.64) * |  |
|  | Intermediate | 4927 (5.4) | 0.83 (0.75, 0.92) * | 0.58 (0.52, 0.63) * | 0.45 (0.41, 0.51) * |  | 0.85 (0.76, 0.95) * | 0.59 (0.53, 0.65) * | 0.47 (0.41, 0.52) * |  |
|  | Ideal | 12597 (3.5) | 0.57 (0.52, 0.63) * | 0.41 (0.37, 0.45) * | 0.29 (0.26, 0.32) * |  | 0.59 (0.53, 0.65) * | 0.42 (0.38, 0.46) * | 0.30 (0.26, 0.33) * |  |
| **Myocardial infarction mortality** | **Lifestyle factors** |  |  |  |  | 0.602 |  |  |  | 0.621 |
|  | Poor | 245 (0.9) | 1.0 (Reference) | 0.89 (0.65, 1.21) | 0.66 (0.43, 0.99) * |  | 1.0 (Reference) | 0.89 (0.65, 1.21) | 0.65 (0.43, 0.99) * |  |
|  | Intermediate | 549 (0.6) | 0.87 (0.63, 1.19) | 0.62 (0.46, 0.83) * | 0.43 (0.30, 0.61) * |  | 0.85 (0.62, 1.17) | 0.61 (0.45, 0.82) * | 0.42 (0.30, 0.60) * |  |
|  | Ideal | 1154 (0.3) | 0.45 (0.33, 0.60) * | 0.38 (0.29, 0.50) * | 0.26 (0.19, 0.36) * |  | 0.43 (0.31, 0.59) * | 0.36 (0.26, 0.50) * | 0.25 (0.18, 0.35) * |  |
| **Stroke** | **Lifestyle factors** |  |  |  |  | 0.011 |  |  |  | 0.010 |
|  | Poor | 1060 (3.8) | 1.0 (Reference) | 0.98 (0.84, 1.14) | 0.86 (0.71, 1.04) |  | 1.0 (Reference) | 0.97 (0.83, 1.13) | 0.85 (0.70, 1.03) |  |
|  | Intermediate | 2884 (3.1) | 0.76 (0.65, 0.88) * | 0.78 (0.68, 0.90) * | 0.72 (0.61, 0.84) * |  | 0.76 (0.65, 0.90) * | 0.79 (0.69, 0.92) * | 0.73 (0.62, 0.85) * |  |
|  | Ideal | 8511 (2.3) | 0.66 (0.58, 0.76) * | 0.57 (0.50, 0.65) * | 0.54 (0.47, 0.63) * |  | 0.67 (0.58, 0.78) * | 0.58 (0.50, 0.67) * | 0.55 (0.47, 0.64) * |  |
| **Stroke mortality** | **Lifestyle factors** |  |  |  |  | 0.862 |  |  |  | 0.844 |
|  | Poor | 248 (0.9) | 1.0 (Reference) | 0.86 (0.63, 1.16) | 0.74 (0.50, 1.10) |  | 1.0 (Reference) | 0.84 (0.62, 1.14) | 0.73 (0.49, 1.08) |  |
|  | Intermediate | 646 (0.7) | 0.70 (0.52, 0.96) * | 0.65 (0.49, 0.86) * | 0.54 (0.39, 0.75) * |  | 0.78 (0.57, 1.06) | 0.72 (0.54, 0.96) * | 0.60 (0.43, 0.83) * |  |
|  | Ideal | 1724 (0.5) | 0.50 (0.38, 0.66) * | 0.43 (0.33, 0.56) * | 0.41 (0.31, 0.54) * |  | 0.61 (0.45, 0.83) * | 0.53 (0.39, 0.71) * | 0.50 (0.37, 0.68) * |  |
| **Heart failure** | **Lifestyle factors** |  |  |  |  | 0.398 |  |  |  | 0.400 |
|  | Poor | 1870 (6.6) | 1.0 (Reference) | 0.84 (0.75, 0.94) * | 0.81 (0.70, 0.93) * |  | 1.0 (Reference) | 0.84 (0.75, 0.94) * | 0.80 (0.69, 0.92) * |  |
|  | Intermediate | 4779 (5.2) | 0.74 (0.66, 0.83) * | 0.65 (0.59, 0.72) * | 0.56 (0.50, 0.63) * |  | 0.66 (0.59, 0.74) * | 0.58 (0.52, 0.65) * | 0.51 (0.45, 0.57) * |  |
|  | Ideal | 10570 (2.9) | 0.45 (0.40, 0.50) * | 0.37 (0.34, 0.41) * | 0.33 (0.29, 0.36) * |  | 0.36 (0.32, 0.40) * | 0.30 (0.27, 0.33) * | 0.26 (0.23, 0.30) * |  |
| **Heart failure mortality** | **Lifestyle factors** |  |  |  |  | 0.783 |  |  |  | 0.773 |
|  | Poor | 332 (1.2) | 1.0 (Reference) | 0.82 (0.63, 1.07) | 0.71 (0.50, 0.99) * |  | 1.0 (Reference) | 0.82 (0.63, 1.06) | 0.70 (0.50, 0.98) * |  |
|  | Intermediate | 780 (0.8) | 0.66 (0.50, 0.86) * | 0.60 (0.47, 0.76) * | 0.48 (0.37, 0.64) * |  | 0.61 (0.46, 0.81) * | 0.56 (0.44, 0.72) * | 0.45 (0.34, 0.60) * |  |
|  | Ideal | 1356 (0.4) | 0.34 (0.27, 0.44) * | 0.27 (0.22, 0.35) * | 0.24 (0.18, 0.31) * |  | 0.29 (0.22, 0.39) * | 0.23 (0.18, 0.30) * | 0.20 (0.15, 0.27) * |  |
| **Atrial fibrillation** | **Lifestyle factors** |  |  |  |  | 0.124 |  |  |  | 0.150 |
|  | Poor | 2635 (9.4) | 1.0 (Reference) | 0.58 (0.53, 0.63) * | 0.31 (0.27, 0.35) * |  | 1.0 (Reference) | 0.58 (0.53, 0.63) * | 0.31 (0.27, 0.35) * |  |
|  | Intermediate | 7756 (8.5) | 0.90 (0.84, 0.98) * | 0.46 (0.43, 0.50) * | 0.26 (0.24, 0.29) * |  | 0.81 (0.75, 0.88) * | 0.42 (0.39, 0.45) * | 0.24 (0.21, 0.26) * |  |
|  | Ideal | 23430 (6.5) | 0.67 (0.63, 0.72) * | 0.35 (0.33, 0.38) * | 0.19 (0.17, 0.20) * |  | 0.53 (0.49, 0.58) * | 0.28 (0.26, 0.30) * | 0.15 (0.14, 0.16) * |  |
| **Atrial fibrillation mortality** | **Lifestyle factors** |  |  |  |  | 0.993 |  |  |  | 0.996 |
|  | Poor | 157 (0.6) | 1.0 (Reference) | 0.58 (0.41, 0.81) * | 0.30 (0.17, 0.54) * |  | 1.0 (Reference) | 0.57 (0.41, 0.81) * | 0.30 (0.17, 0.53) * |  |
|  | Intermediate | 395 (0.4) | 0.76 (0.56, 1.05) | 0.40 (0.30, 0.54) * | 0.21 (0.14, 0.31) * |  | 0.69 (0.50, 0.95) * | 0.36 (0.27, 0.49) * | 0.19 (0.12, 0.29) * |  |
|  | Ideal | 889 (0.2) | 0.43 (0.32, 0.58) * | 0.23 (0.17, 0.31) * | 0.12 (0.09, 0.17) * |  | 0.36 (0.25, 0.50) * | 0.19 (0.14, 0.26) * | 0.10 (0.07, 0.15) * |  |

Note, PRS, polygenic risk score; the PRSs presented are specifically used based on corresponding outcomes; Model 1 was adjusted for age, sex, race, Townsend Deprivation Index, annual household income, education attainment, 22 assessment centers, and the first 5 principal components of ancestry. Model 2 was further adjusted for family history of diabetes and family history of high blood pressure, based on Model 1; **^a^** likelihood tests was applied to test the significance of interaction term by comparing the model with and without the interaction term; **P* < 0.05.
